# Research Progresses in Immunological Checkpoint Inhibitors for Breast Cancer Immunotherapy

**DOI:** 10.3389/fonc.2021.582664

**Published:** 2021-09-23

**Authors:** Wenxiang Zhang, Xiangyi Kong, Bolun Ai, Zhongzhao Wang, Xiangyu Wang, Nianchang Wang, Shan Zheng, Yi Fang, Jing Wang

**Affiliations:** ^1^ Department of Breast Surgical Oncology, China National Cancer Center/Cancer Hospital, Chinese Academy of Medical and Peking Union Medical College, Beijing, China; ^2^ Department of Cancer Prevention, China National Cancer Center/Cancer Hospital, Chinese Academy of Medical and Peking Union Medical College, Beijing, China; ^3^ Department of Pathology, China National Cancer Center/Cancer Hospital, Chinese Academy of Medical and Peking Union Medical College, Beijing, China

**Keywords:** breast cancer, immunological checkpoint inhibitor, immunotherapy, cytotoxicity T lymphocyte antigen 4, programmed cell death protein 1/programmed cell death protein-ligand 1

## Abstract

Tumor immune escape refers to the phenomenon in which tumor cells escape the recognition and attack of the body’s immune system through various mechanisms so that they can survive and proliferate *in vivo*. The imbalance of immune checkpoint protein expression is the primary mechanism for breast cancer to achieve immune escape. Cytotoxic T lymphocyte antigen 4 (CTLA4) and programmed cell death protein 1 (PD-1)/programmed cell death protein-ligand 1 (PD-L1) are critical immune checkpoints for breast cancer. Immune checkpoint inhibitors block the checkpoint and relieve its inhibition effect on immune cells, reactivate T-cells and destroy cancer cells and restore the body’s ability to resist tumors. At present, immunological checkpoint inhibitors have made significant progress in breast cancer immunotherapy, and it is expected to become a new treatment for breast cancer.

## Introduction

Despite significant advances in diagnosis, surgery, endocrine therapy, chemotherapy, and molecular-targeted therapy, breast cancer remains the second leading cause of cancer death in women (1). In recent years, tumor immune evasion has been recognized as a hallmark of cancer progression (2), which could modulate innate immune and suppress T-cells (3), leading to tumor growth and progression. Nowadays, immunotherapy has attracted widespread attention in the field of cancer treatment. Unlike traditional surgery, radiotherapy and chemotherapy, immunotherapy is a combination of conventional therapy and immunomodulation, which exploits the body’s immune system to attack tumors. At present, agents used for immunotherapy mainly include tumor antigen vaccine, dendritic cell activator, adjuvant to induce innate immunity, adjuvants that activate innate immunity, and immunological checkpoint inhibitors (4). Among them, immune checkpoint inhibitors play a vital role in maintaining autoimmune tolerance and avoiding the attack of the normal tissues by the immune system. This review summarizes the progress in immunological checkpoint inhibitors for breast cancer immunotherapy.

## Immune Checkpoint and Immune Escape

### Immune Escape

**Figure 1:**
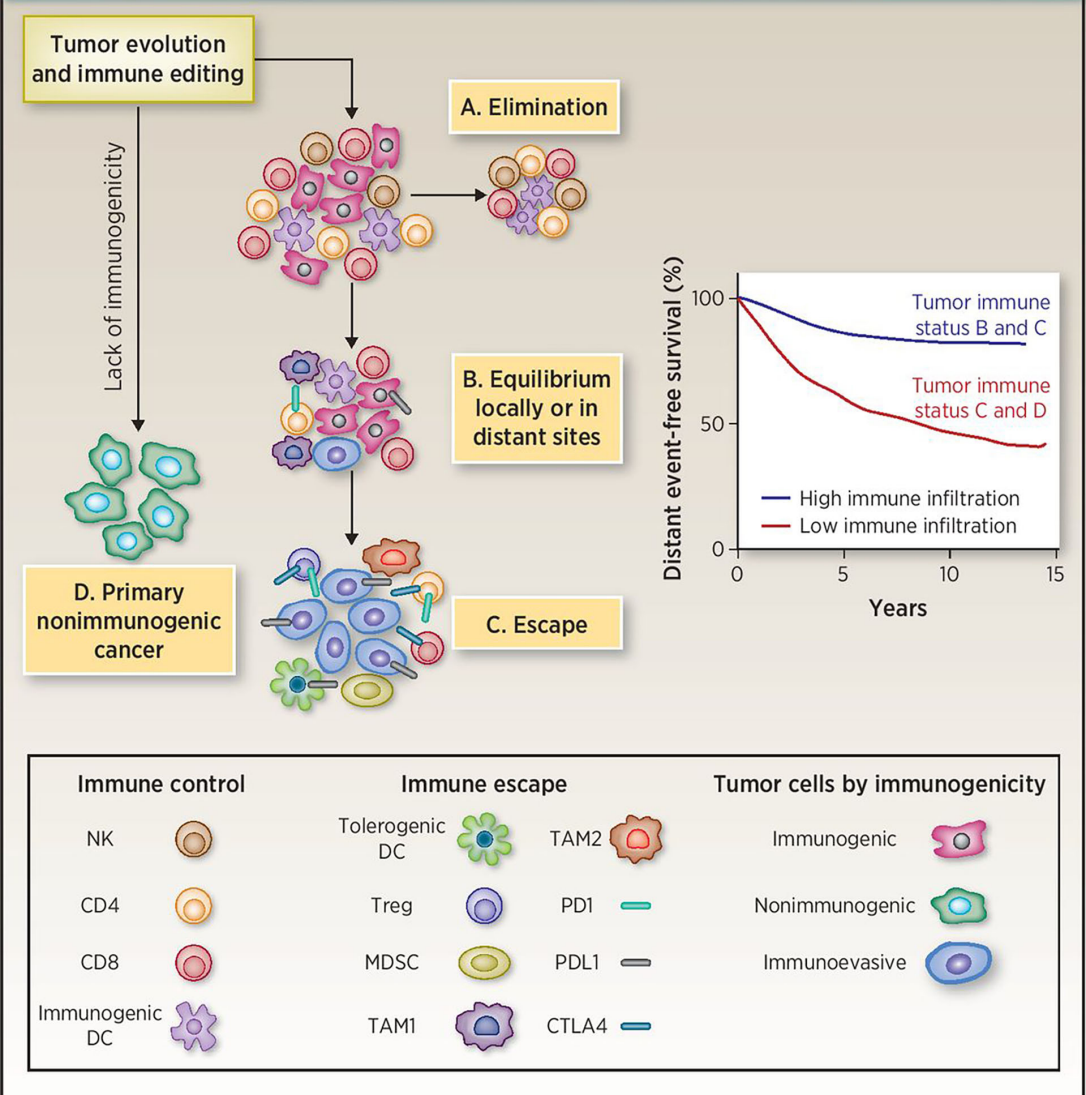
Immunoediting during tumor evolution. **(A)** All clinically apparent early breast cancers are already partially edited or not immunogenic enough since the elimination phase has failed. **(B)** Tumors in the equilibrium phase are likely represented in the high immune infiltration group. Recurrences in this group are at least in part due to subsequent immune escape. **(C, D)** Tumors with low immune infiltration may include cancers with intrinsically low immunogenicity and cancers that have effectively escaped from immune surveillance. DC, dendritic cells; MDSC, myeloid-derived suppressor cells; TAM1, tumor-associated macrophages M1 or classically activated; TAM2, tumor-associated macrophages M2 or alternatively activated. Reprinted with permission from Pusztai L, Karn T, Safonov A, Abu-Khalaf MM and Bianchini G. New Strategies in Breast Cancer: Immunotherapy. Clin Cancer Res 2016; 22: 2105-2110 (5). Copyright ^©^ 2016, American Association for Cancer Research (AACR).

In recent years, the role of the immune system in the recognition and control of breast cancer progression has been the focus of debate in recent years. The immune system carries out immune responses and other immune functions of the body. Under physiological conditions, the immune system constantly patrols the body to recognize and destroy invading pathogens and cancerous cells. However, in many malignant tumors, immune surveillance is often ineffective. Dunn et al. proposed the “cancer immune editing” (**Figure 1**) hypothesis in 2004 to explain the process by which tumor cells evade immune recognition and elimination, which can be divided into three phases (6). (1) Immune elimination: the immune system recognizes and eradicates growing tumor cells through innate and adaptive immune responses. In this phase, cancer cells are either completely eradicated or drug-resistant clonal variants are produced by decreasing their immunogenicity and/or secreting and recruiting immunosuppressive factors(such as IL-10 and tumor growth factor-beta (TGF-beta). (2) Immune equilibrium: at this stage, proliferation of tumor cells and elimination of tumor cells by the immune system reaches an equilibrium. (3) Immune escape: tumor cells that had escaped immune equilibrium gained the ability to evade immune monitoring and elimination, and with local immunity suppressed, the tumor becomes symptomatic. In the immune escape mechanism of breast cancer, the intrinsic resistance factors of tumor cells including MAPK signal, PTEN mutation, WNT-β-catenin signal activation, IFN-γ signal activation, and adaptive resistance factors, such as expression immunity Checkpoint molecules, tumor stromal proliferation, and immune cell infiltration are all involved in this process (7–11). In addition, breast cancer has always considered as a cold tumor, the loss of tumor antigens, expression of Fas ligand (FasL) and programmed cell death 1 ligand, and production of immunosuppressive cytokines such as TGF-beta and IL-10 are some of the mechanisms of immune escape.

As a group of active immune surveillance cells, TILs can attack pathogens, xenobiotics, and mutant tumor cells. Most TILs are T cells. T cell dysfunction in the tumor microenvironment is a key mechanism for tumor cells to escape immune surveillance, In breast cancer patients, signs of immune response exhaustion begin to appear in the early stages of tumor development, and the immunosuppression in the tumor is stronger than that in the blood, which contributes to the immune escape during the development and metastasis of breast cancer (9). long-term continuous exposure of T cells to tumor antigens gradually leads to T cell dysfunction and ultimately T Cell Exhaustion, characterized by loss of T cell proliferation, decreased cytokine secretion, and inability to kill target cells (12). Fortunately, this exhaustion can be partly reversed, mainly by blocking the inhibitory checkpoint pathways PD-1 or PD-L1 (13).

### Immune Checkpoint

Immune checkpoints are the inhibitory signal pathway that exists in the immune system, which regulates the intensity and persistence of immune response in peripheral tissues to prevent tissue damage, and plays a role in maintaining autoimmune tolerance (14). Studies have found that tumor cells can use this property of immune checkpoints to prevent the elimination of immune system by abnormally expressing these molecules and achieve immune escape (15). In a review, Pardoll DM et al. (16) described that the dysregulation of immune checkpoint protein expression is the main mechanism of immune escape in breast cancer. In clinical research, immune checkpoint inhibitors can release the brakes of the immune system by interacting with these immune checkpoints, reactivate immune cells to kill cancer cells and restore the body’s own anti-tumor immune response. The main immune checkpoints for breast cancer include cytotoxic T-lymphocyte-associated protein-4 (CTLA-4), programmed death receptor 1/programmed cell death ligand 1 (PD-1/L1), lymphocyte activation gene 3 (LAG-3), T cell immunoglobulin domain and mucin 3 (TIM-3) and other molecules.

## Immunological Checkpoint Inhibitors Targeting CTLA⁃4

Both CTLA-4 and CD28 are members of the immunoglobulin superfamily, and they bind to the same ligands CD86 (B7-2) and CD80 (B7-1), but CTLA-4 binds with a greater affinity. The key to the immune regulation function of CTLA-4 is to control CD4+FoxP3-, CD8+T cells and regulatory T cells (Treg). CTLA-4 can blockade activated T cell response and mediate the inhibitory of regulatory T cells (Tregs). At present, CTLA-4 inhibits the response of T cells mainly in two ways: one is to reduce the signal of TCR (T cell receptor) and CD28 by binding B7 competitively with CD28 or recruiting phosphatase to the intracellular domain of CTLA-4. The other is to reduce the expression level of CD80 and CD86 on antigen-presenting cells (APC) or remove them from APC by transendocytosis, which reduces their availability for CD28 engagement and costimulation in T cell activation. Ipilimumab and Tremelimumab are the two currently available antibodies targeting CTLA⁃4, which have been widely used in the treatment of melanoma, kidney cancer, prostate cancer, lung cancer, etc. (17). Some ongoing clinical trials of anti-CTLA-4 immunotherapeutic interventions of malignancies including breast cancer are summarized in **Table S1**.

### Ipilimumab

**Figure 2:**
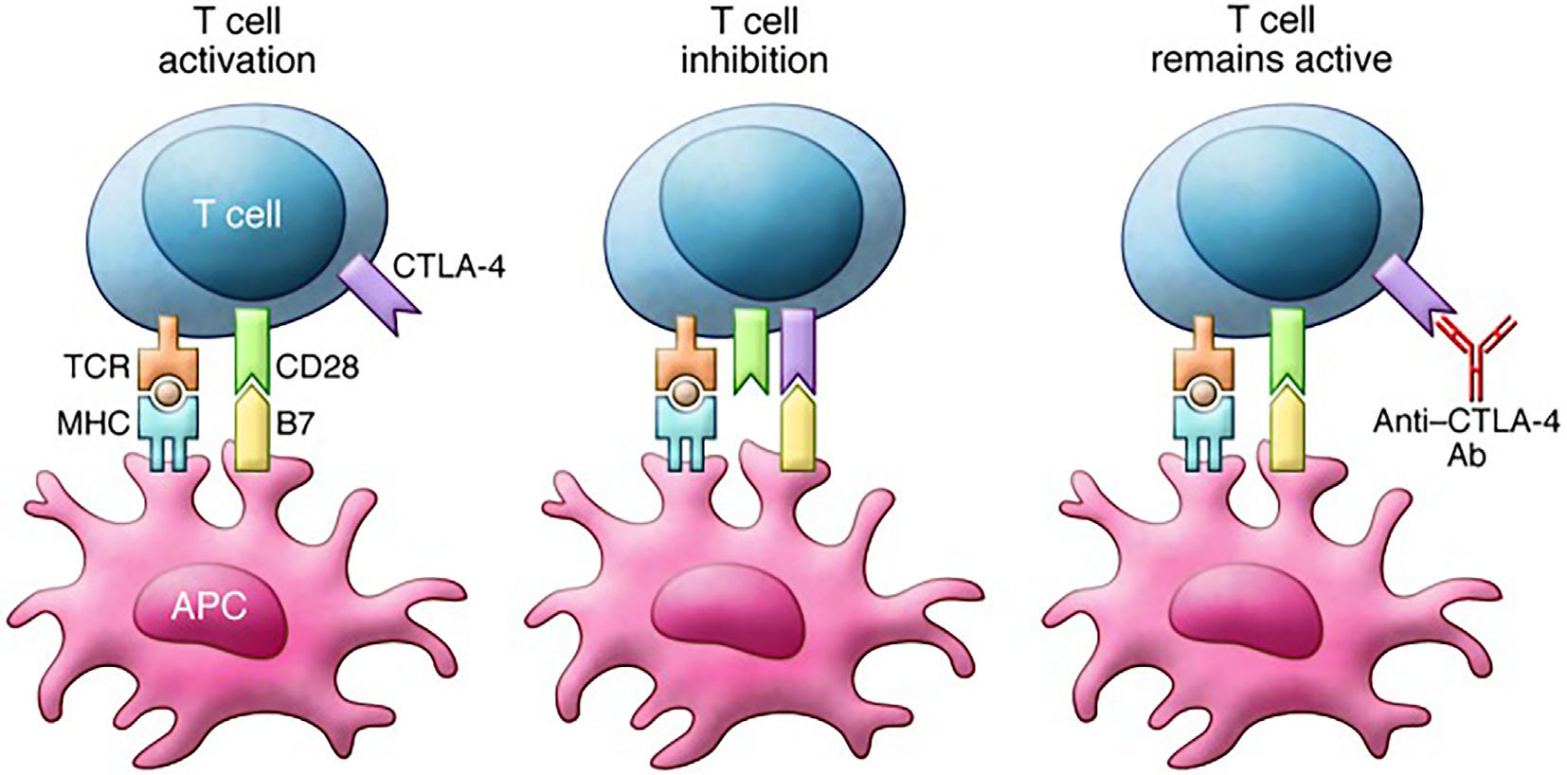
Mechanism of action of ipilimumab. T cell activation requires stimulation through both the TCR and CD28. Binding of B7 family member proteins to CTLA-4 inhibits T cell function. Notably, CTLA-4 expression increases in parallel with TCR stimulation, thereby serving as a break on T cell responses. Anti–CTLA-4 antibodies such as ipilimumab block CTLA-4 binding to B7 proteins and prevent inhibition of T cell function. Reprinted with permission from Buchbinder E and Hodi FS. Cytotoxic T lymphocyte antigen-4 and immune checkpoint blockade. J Clin Invest 2015; 125: 3377-3383 (19). Copyright ^©^ 2015, American Society for Clinical Investigation.

Ipilimumab is the first human CTLA⁃4 antibody that reactivates “silenced” or “depleted” T cells by binding to CTLA⁃4, and helps them attack tumor cells (18). The US Food and Drug Administration has approved Ipilimumab for the treatment of advanced melanoma. The mechanism of action of ipilimumab is shown in **Figure 2**. Recently published studies on the applications of Ipilimumab in various cancers are shown in **Table 1** (20–29). McArthur et al. studied the safety and tolerability of Ipilimumab-mediated immune suppression in breast cancer (29). In this study, 19 with breast cancer were randomized to a preoperative tumor group, a preoperative monotherapy group (Ipilimumab, intravenous 10 mg/kg), and a preoperative tumor cryoablation plus monotherapy group (Ipilimumab, intravenous 10 mg/kg). Early results showed that all three treatment regimens were safe and tolerable, and compared with preoperative tumor cryoablation or preoperative monotherapy, preoperative tumor cryoablation plus monotherapy increased the type 1 anti-tumor immune response, the ratio of effector to regulatory intra-tumoral T cells, and the number of peripheral activated T cells, showing a synergistic anti-tumor potential.

**Table 1:** Recent published studies on the application of Ipilimumab in various Cancer.

Author	Year	Institution	Country	Journal	Cancer Type	Research Type	Rearch Purpose	Key Conclusions
Antonia et al. (20)	2016	Centro Integral Oncológico Clara Campal	USA and Spain	Lancet Oncol	Recurrent small-cell lung cancer	Multicentre, open-label, phase 1/2 trial	To assess safety and activity of nivolumab and nivolumab plus ipilimumab in patients with small-cell lung cancer (SCLC) who progressed after one or more previous regimens.	Nivolumab monotherapy and nivolumab plus ipilimumab showed antitumour activity with durable responses and manageable safety profiles in previously treated patients with SCLC, suggesting a potential new treatment approach for a population of patients with limited treatment options and support the evaluation of nivolumab and nivolumab plus ipilimumab in phase 3 randomised controlled trials in SCLC.
Reck et al. (21)	2016	LungenClinic Grosshansdorf	Germany	J Clin Oncol	Extensive-Stage Small-Cell Lung Cancer	Phase III Randomized Trial	To evaluate the efficacy and safety of ipilimumab or placebo plus etoposide and platinum in patients with newly diagnosed extensive-stage disease SCLC.	1) Addition of ipilimumab to chemotherapy did not prolong OS versus chemotherapy alone in patients with newly diagnosed extensive-stage disease SCLC; 2) No new or unexpected adverse events were observed with chemotherapy plus ipilimumab.
Hellmann et al. (22)	2017	Memorial Sloan Kettering Cancer Center	USA	Lancet Oncol	Advanced non-small-cell lung cancer	Open-label, phase 1, multicohort study	To assess the safety and activity of combination nivolumab plus ipilimumab as first-line therapy for NSCLC.	In NSCLC, first-line nivolumab plus ipilimumab had a tolerable safety profile and showed encouraging clinical activity characterised by a high response rate and durable response.
Beer et al. (23)	2017	Oregon Health and Science University	USA	J Clin Oncol	Metastatic Chemotherapy-Naive Castration-Resistant Prostate Cancer.	Phase III Randomized Trial	To assess Ipilimumab increases antitumor T-cell responses by binding to cytotoxic T-lymphocyte antigen 4	1) Ipilimumab did not improve OS in patients with metastatic castration-resistant prostate cancer. 2) The observed increases in progression-free survival and prostate-specific antigen response rates suggest antitumor activity in a patient subset.
Bang et al. (24)	2017	Seoul National University College of Medicine	Korea	Clin Cancer Res	Unresectable Locally Advanced/Metastatic Gastric or Gastroesophageal Junction Cancer.	Phase II	To evaluate the safety and efficacy of ipilimumab monotherapy versus best supportive care (BSC) among patients with advanced/metastatic gastric or gastroesophageal junction cancer who achieved at least stable disease with first-line chemotherapy.	Although ipilimumab at 10 mg/kg was manageable, it did not improve irPFS versus BSC. However, comparable median OS of approximately 1 year and a favorable safety profile support the investigation of ipilimumab in combination with other therapies for advanced gastric cancer.
Govindan et al. (25)	2017	Washington University School of Medicine	USA	Transl Lung Cancer Res	Advanced Squamous Non-Small-Cell Lung Cancer.	Phase III Randomized Trial	To investigate the efficacy and safety of first-line ipilimumab or placebo plus paclitaxel and carboplatin in advanced squamous NSCLC.	1) The addition of ipilimumab to first-line chemotherapy did not prolong OS compared with chemotherapy alone in patients with advanced squamous NSCLC. 2) The safety profile of chemotherapy plus ipilimumab was consistent with that observed in previous lung and melanoma studies. Ongoing studies are evaluating ipilimumab in combination with nivolumab in this population.
Yi et al. (26)	2017	Duke University Medical Center	USA	Clin Cancer Res	Early-Stage Non-Small Cell Lung Cancer	Phase II	To determine the immunologic effects of neoadjuvant chemotherapy plus ipilimumab in early-stage non-small cell lung cancer (NSCLC) patients	1) This study did not meet the primary endpoint of detecting an increase in blood-based TAA T-cell responses after ipilimumab. Collectively, these results highlight the immune activating properties of ipilimumab in early-stage NSCLC. 2) The immune profiling data for ipilimumab alone can contribute to the interpretation of immunologic data from combined immune checkpoint blockade immunotherapies.
Hellmann et al. (27)	2018	Memorial Sloan Kettering Cancer Center Hospital	USA	N Engl J Med	Lung cancer	Open-label, multipart, phase 3 trial	To examine progression-free survival with nivolumab plus ipilimumab versus chemotherapy among patients with a high tumor mutational burden (≥10 mutations per megabase).	Progression-free survival was significantly longer with first-line nivolumab plus ipilimumab than with chemotherapy among patients with NSCLC and a high tumor mutational burden, irrespective of PD-L1 expression level. The results validate the benefit of nivolumab plus ipilimumab in NSCLC and the role of tumor mutational burden as a biomarker for patient selection.
Kwek et al. (28)	2015	Division of Hematology/Oncology, University of California	USA	Cancer Immunol Res	Prostate Cancer	Phase Ib	To enhance adaptive immune responses without an exogenous vaccine, but the immunologic biomarkers associated with improved clinical outcome in cancer patients are not fully established.	Preexisting expression of immunologic checkpoint marker PD-1 on CD4 Teff cells may help identify patients that may benefit from ipilimumab treatment.

### Tremelimumab (CP⁃675206)

Tremelimumab is a humanized CTLA⁃4 monoclonal antibody that highly selectively blocks the interaction of CTLA⁃4 with B7 and enhances T cell activation (30). Recently published studies on the applications of Tremelimumab in various cancers are shown in **Table 2** (31–34). Vonderheide et al. found that a combination of Tremelimumab and Exemestane resulted in a stable response in 42% (11/26) of the patients for over 12 weeks in an early phase I clinical trial. In addition, This research also shows that most of this therapeutic effect was related to the increase in peripheral CD4+ and CD8+ T cells expressing inducible costimulators (ICOS) and the significant increase in the ratio of ICOS+ T cells to FoxP3+ regulatory T cells (35). Moreover, in a small phase I dose-escalation study of local radiation and tremelimumab in patients with inoperable locally recurrent or metastatic breast cancer, Jiang, DM et al. assessed the safety of tremelimumab and observed the maximum tolerated dose (MTD) of tremelimumab combined with RT and found tremelimumab at 3 mg/kg combined with RT appears to be a tolerable treatment strategy (36). Recently, Santa-Maria et al. designed a single-arm pilot research to determine the overall response-rate (ORR) of durvalumab plus tremelimumab, and assessed the immunogenomic-dynamics in metastatic endocrine receptor (ER) positive or triple negative breast cancer (TNBC) (37). According to the pre-designed, only three patients responded (ORR = 17%), so the study did not enter the second stage, but at the same time, they found that a higher clinical benefit rate was observed in TNBC (ORR = 43%), Compared to non-responders, a higher mutational and neoantigen burden among responders, intimating immunogenomic dynamics may help identify phenotypes most likely to respond to immunotherapy (37).

**Table 2:** Recent published studies on the applications of Tremelimumab in various cancers.

Author	Year	Institution	Country	Journal	Cancer Type	Research Type	Rearch Purpose	Key Conclusions
Xie C et al. (31)	2018	Hematology/Oncology Fellowship Program, National Heart, Lung, and Blood Institute/National Cancer Institute, National Institutes of Health.	USA	Hepatology	Hematology/Oncology Fellowship Program, National Heart, Lung, and Blood Institute/National Cancer Institute, National Institutes of Health.	Phase I, Interventional, Non-Randomized, Sequential Assignment, Open Label	To investigate whether tremelimumab, an anti-CTLA4 inhibitor, could be combined safely with microwave ablation to enhance the effect of anti-CTLA4 treatment in patients with advanced BTC.	1) Tremelimumab in combination with tumor ablation is a potential new treatment strategy for patients with advanced BTC. 2) Increased circulating activated CD8+ T cells and TCR repertoire expansion induced by tremelimumab may contribute to treatment benefit. 3) This article is protected by copyright. All rights reserved.
Fumet et al. (32)	2018	Center Georges Francois Leclerc	France	ESMO Open	Metastatic colorectal cancer (mCRC)	Phase II	To evaluate whether the addition of PD-L1 and CTLA-4 inhibition to oxaliplatin, fluorouracil and leucovorin (FOLFOX) increases treatment efficacy.	1) Immunogenic chemotherapy in combination with immune checkpoint would act synergistically and might be a promising treatment for metastatic CRC. 2) Furthermore, blood, plasma and tumour tissue will be collected and assessed for potential prognostic and predictive biomarkers.
Antonia et al. (33)	2016	H Lee Moffitt Cancer Center	USA	Lancet Oncol	Advanced squamous or non-squamous non-small cell lung cancer (NSCLC)	Multicentre, non-randomised, open-label, phase 1b study	To assess durvalumab plus tremelimumab in patients with advanced squamous or non-squamous non-small cell lung cancer (NSCLC).	Durvalumab 20 mg/kg every 4 weeks plus tremelimumab 1 mg/kg showed a manageable tolerability profile, with antitumour activity irrespective of PD-L1 status, and was selected as the dose for phase 3 studies, which are ongoing.
Aglietta et al. (34)	2014	University of Torino	Italy	Ann Oncol	Metastatic pancreatic cancer.	Phase Ib, multisite, open-label, nonrandomized dose escalation trial	Evaluating the safety, tolerability, and maximum tolerated dose (MTD) of tremelimumab combined with gemcitabine in patients with metastatic pancreatic cancer.	Tremelimumab plus gemcitabine demonstrated a safety and tolerability profile, warranting further study in patients with metastatic pancreatic cancer.

## Immunological Checkpoint Inhibitors Targeting PD⁃1/PD⁃L1

PDL1 are often expressed on the surface of tumor cells and immune cells such as activated T cells, B cells, and natural killer cells. PD⁃1/PD⁃L1 is a pair of immune co-inhibitory molecules – binding of PD⁃L1 on the surface of tumor cells to PD⁃1 on the surface of activated T cells can inhibit activation of T cells, leading to immunosuppression and immune escape (38). PD⁃1/PD⁃L1 also inhibits T cell proliferation and differentiation by inhibiting mitogen-activated extracellular signal-regulated kinase/protein kinase B/RAS signaling pathways (38–40). In the tumor microenvironment, PD⁃L1 also induces TILs exhaustion, impairing immune surveillance (41). Also, PD⁃L1 can also induce Treg cell proliferation, thus indirectly enhance immune suppression (42). Unlike CTLA⁃4 that mediates immunosuppression early at antigen presentation, PD⁃1-mediates immunosuppression mainly by regulating peripheral T cells in late immune response (43). Blocking PD⁃1/PD⁃L1 signaling reverses the immunosuppression in the tumor microenvironment and enhances antitumor activity. Interaction between PD⁃1/PD⁃L1 may be a target for anticancer therapy. Studies have shown that PD⁃1/PD⁃L1 antibody promoted regression of the persistent tumor. The objective response rate varied among different malignant tumors, generally between 20% to 25% (44). Some ongoing clinical trials of anti- PD-L1/PD-L1 immunotherapeutic interventions of malignancies including breast cancer are shown in **Tables S2**, **S3**.

### Pembrolizumab (Lambrolizumab or MK⁃3475)

Pembrolizumab is a potent, highly selective, humanized monoclonal antibody that blocks the interaction between PD-1 and its ligands PD-L1 and PD-L2, thereby activating T lymphocytes, which may affect both tumor cells and healthy cells (40). It has been shown to have antitumor activity and a range of mainly low-grade toxic effects in patients with early TNBC or metastatic TNBC.

In a phase 1b KEYNOTE-173 study of neoadjuvant pembrolizumab plus chemotherapy, with or without carboplatin, for high-risk, early-stage, non-metastatic triple-negative breast cancer (TNBC), The overall pathological complete response (pCR) rate was nearly 60% and the dose-limiting toxicities occurred in 22 patients(36.7%). In addition, the pCR rate showed a positive correlation with tumor PD-L1 expression and sTIL levels in an exploratory analysis (45). Also in the phase 2 I-SPY2 trial (46), Nanda R et al. found that the addition of Pembrolizumab in combined with olaparib and paclitaxel more than doubled the pCR for both HR-positive/HER2-negative and TNBC patients. Currently, the phase III KEYNOTE-522 trial were presented during the 2019 ESMO Scientific Meeting and the latest results have been published in the New England Journal of Medicine (47). It is the first phase III randomized controlled study evaluating pembrolizumab combination therapy for neoadjuvant/adjuvant therapy in early triple-negative breast cancer. The study included 1174 patients who were divided into two groups (in a 2:1 ratio) and the two groups were randomly assigned to receive 4 cycles of pembrolizumab (every three weeks) plus paclitaxel and carboplatin neoadjuvant therapy, or placebo every 3 weeks plus paclitaxel and carboplatin, while both groups received doxorubicin-cyclophosphamide or epirubicin-cyclophosphamide. After definitive surgery, the two groups received 9 cycles of adjuvant pembrolizumab or placebo, the results demonstrated that the pathological complete remission rate of pembrolizumab combined with chemotherapy was significantly higher than that of placebo combined with chemotherapy in patients with early triple-negative breast cancer(64.8% vs 51.2%). On February 9, 2021, the Oncology Drug Advisory Committee (ODAC) held a meeting on the supplementary listing application of Keytruda (pembrolizumab) for preoperative neoadjuvant therapy and then using Keytruda (pembrolizumab) as a single drug for postoperative adjuvant therapy. The ODAC unanimously voted 10-0 against approval of indication, and should wait until the mature clinical data of KEYNOTE-522 before making a decision. However, in May 2021,Merck Announces phase 3 KEYNOTE-522 Trial met another primary endpoint of event-free survival (EFS) in patients with high-risk early-stage TNBC. The results of the study showed that EFS was statistically significantly improved compared to the control group (48). In view of the results of this study and the FDA’s attitude towards this study, we have some doubts. First, can pCR reflect the survival benefits of Keytruda to patients? Secondly, there is a flaw in the design of this study. Is Keytruda used as a preoperative adjuvant therapy, postoperative adjuvant therapy, or a combination of both? Finally, although the current benefits of EFS are worthy of recognition, it is still not clear how the improvement of pCR affects the improvement of EFS, and the relationship between the two still needs further observation and research.

For the monotherapy of Pembrolizumab in the treatment of advanced or metastatic TNBC, Pembrolizumab showed durable antitumor activity and manageable safety in the single-arm KEYNOTE-012 (49) and II KEYNOTE-086 trial (50, 51). Unfortunately, in the randomized phase III KEY-NOTE-119 (NCT02555657) study, pembrolizumab monotherapy did not show an improvement in ORR, PFS, or OS as compared to single-agent chemotherapy in participants with previously treated mTNBC. However, it seems that patients with the highest levels of tumor PD-L1 expression had the greatest benefit regarding an ORR and the median OS with ICI in *post hoc* analyses, although these subgroup analyses should be interpreted with caution (52). Such results suggest the need for further research on the effect of pembrolizumab on selected subgroups of patients, especially PD-L1 enriched tumors, and explore the efficacy of the combined regimen in the treatment of mTNBC patients.

When it comes to combination therapy, the most important phase III randomized study is the KEYNOTE-355 trial, this study evaluated the efficacy and safety of pembrolizumab plus chemotherapy with placebo plus chemotherapy as first-line treatment for patients with advanced TNBC. Pembrolizumab Plus Chemotherapy Significantly Improved PFS Compared to Chemotherapy Alone in Patients with mTNBC Whose Tumors Expressed PD-L1 (CPS ≥10)(9.7 months vs 5.6 months) (53). Based on the recommendation of the DMC, the trial will continue without changes to evaluate the other dual primary endpoint of overall survival (OS). On November 13, 2020, based on the PFS results of KEYNOTE-355, the FDA accelerated the approval of pembrolizumab combined with chemotherapy (albumin paclitaxel/paclitaxel/gemcitabine + carboplatin) for patients with unresectable locally advanced or metastatic triple-negative breast cancer (TNBC) whose tumors express PD-L1(CPS≥10), as determined by an FDA-approved test(Dako PD-L1 (22C3). Although the results of this trial are consistent with the phase 3 IMPASSION130 trial, we have noticed that the two experiments used different PD-L1 detection methods, there was approximately 80% concordance in patients captured by immune cell 1% and above (by SP142 assay) and CPS of 10 or more (54), and both assays identified approximately 40% of the intention-to-treat populations that benefited from immunotherapy plus chemotherapy, these two assays should not be considered as interchangeable (55). Whether antibodies SP142 (IC >= 1%) or the 22C3 (CPS >= 10) is the benchmark, there is no conclusion yet, it is worthy of our exploration.

### Atezolizumab (Tecentriq, MPDL3280A, MEDI4736 or BMS⁃936559)

**Figure 3:**
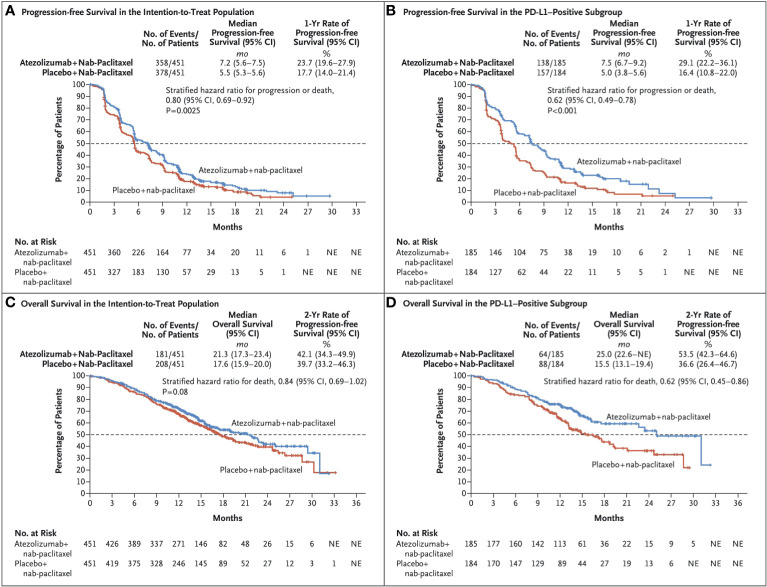
Kaplan–Meier Analysisof Progression-free Survival and Overall Survival. Shown are Kaplan–Meier estimates of progression free-survival, according to the Response Evaluation Criteria in Solid Tumors, version 1.1, as assessed by the investigators, among patients in the intention-to treat population **(A)** and among patients whose tumors were positive for programmed death ligand 1(PD-L1) expression (≥1% PD-L1 expression on tumor infiltrating immune cells [PD-L1–positive subgroup]) **(B)**. Also shown are the Kaplan–Meier estimates of overall survival in the intention-to-treat population **(C)** and the PD-L1–positive subgroup **(D)**. Stratified hazard ratios for disease progression or death (in analyses of progression-free survival) or for death (in analyses of overall survival) are reported along with P values. Tick marks indicate censored data, and the dashed line indicates the median. Reprinted with permission from Schmid P, Adams S, Rugo HS, et al: Atezolizumab and Nab-Paclitaxel in Advanced Triple-Negative Breast Cancer. N Engl J Med 2018, 379(22):2108-2121 (57). Copyright ^©^ 2019 Massachusetts Medical Society.

Atezolizumab is the first humanized anti-PD-L1 monoclonal immunoglobulin G1 antibody approved by the U.S. Food and Drug Administration (FDA), which binds to PD-L1 expressed on tumor cells and tumor-infiltrating immune cells, blocked its interaction with the interaction of PD-1 and B7.1 receptor, restores T cell function, and relieves inhibition of the body’s immune system against tumor cells (56, 57). It is successively approved for the treatment of patients with metastatic non-small cell lung cancer and advanced urothelial cancer whose disease progressed despite platinum-containing chemotherapy (58). On March 8, 2019, the FDA accelerated approved atezolizumab in combination with paclitaxel protein-bound for the treatment of adult patients with unresectable locally advanced or metastatic triple-negative breast cancer. This is mainly based on the Impassion130 clinical trial (NCT02425891). it was a phase III, international, randomized, double-blind, placebo-controlled study, which aims to evaluate the efficacy of atezolizumab in combination with nab-paclitaxel l versus placebo with nab-paclitaxel as first-line treatment for unresectable patients with locally advanced or metastatic TNBC (59). Through intention-to-treat analysis, the investigators found that atezolizumab plus nab-paclitaxel notably prolonged median progression-free survival (PFS) in comparison with the placebo group [7.2 months versus 5.5 months; hazard ratio (HR) for progression or death, 0.80; 95% CI, 0.69-0.92; p = 0.002] in the intention-to-treat patients, especially in the PD-L1-positive subgroup (7.5 months versus 5.0 months; HR for progression or death, 0.62; 95% CI, 0.49-0.78; p < 0.001), the period is significantly extended (**Figure 3**). There was no significant difference in OS between the treatment groups in the ITT population, but in the exploratory overall survival analysis in patients with PD-L1 immune cell-positive tumors, median overall survival was 25·0 months (95% CI 19·6–30·7) with atezolizumab versus 18·0 months (13·6–20·1) with placebo (stratified HR 0·71, 0·54–0·94)). We should rationally interpret such research results. with regard to the selection of the test sequence, the study adopted the hierarchical testing design for the analysis of OS in the ITT and PD-L1(+) populations. This means the OS would be tested in the PD-L1–positive subgroup population only if the OS was significantly improved in the ITT populations, in the era of precision medicine, this design sequence is worth rethinking.

After the results of the Impassion130 study were announced, Impassion131e quickly revised the protocol. The main research endpoint was changed from the original PFS of the ITT population to the PFS of the PD-L1+ population first, the sample size has also increased from 495 to 651patients. Impassion131 is a phase III randomized study designed to compare the efficacy and safety of atezolizumab+paclitaxel versus placebo+paclitaxel in 651unresectable locally advanced/metastatic triple-negative breast cancer patients. The results of the study showed that atezolizumab-paclitaxell did not significantly reduce the risk of cancer progression and death in the PD-L1 positive population. In addition, whether in the PD-L1 positive population or in the intention-to-treat (ITT) population, the interim OS results support the combination of paclitaxel-placebo (28.3 months)rather than paclitaxel combined with atilizumab(22.1 months). Such results surprised breast experts, who tried to explain the reasons for the different results. Most experts believe that the different taxanes between the two studies and the imbalance of invisible differences between the study arms of Impassion131 are the main reason. In a recent article, Van Wambeke S et al. (60) put forward a relatively novel point of view. They emphasized that we should pay more attention to whether Impassion130 is a false positive or not, they are more concerned about the signal of harm seen in Impassion131, Similarly, advocacy organizations for patients with breast cancer also came to the conclusion “the only results that we have in the setting of breast cancer from appropriately powered analyses show no overall survival benefit with atezolizumab, or a negative trend in OS, and either no or a 2.6-month benefit of PFS in patients with TNBC, regardless of their PD-L1 expression” (61).

Because of the results of IMPassion131, On September 8, 2020, the FDA issued an alert about the efficacy and potential safety concerns with atezolizumab plus paclitaxel, At present, the results of such an exploratory study(IMPassion130)are a bit convincing. In the future, the results of IMPassion130 should be confirmed by follow-up study.

### Avelumab

**Figure 4:**
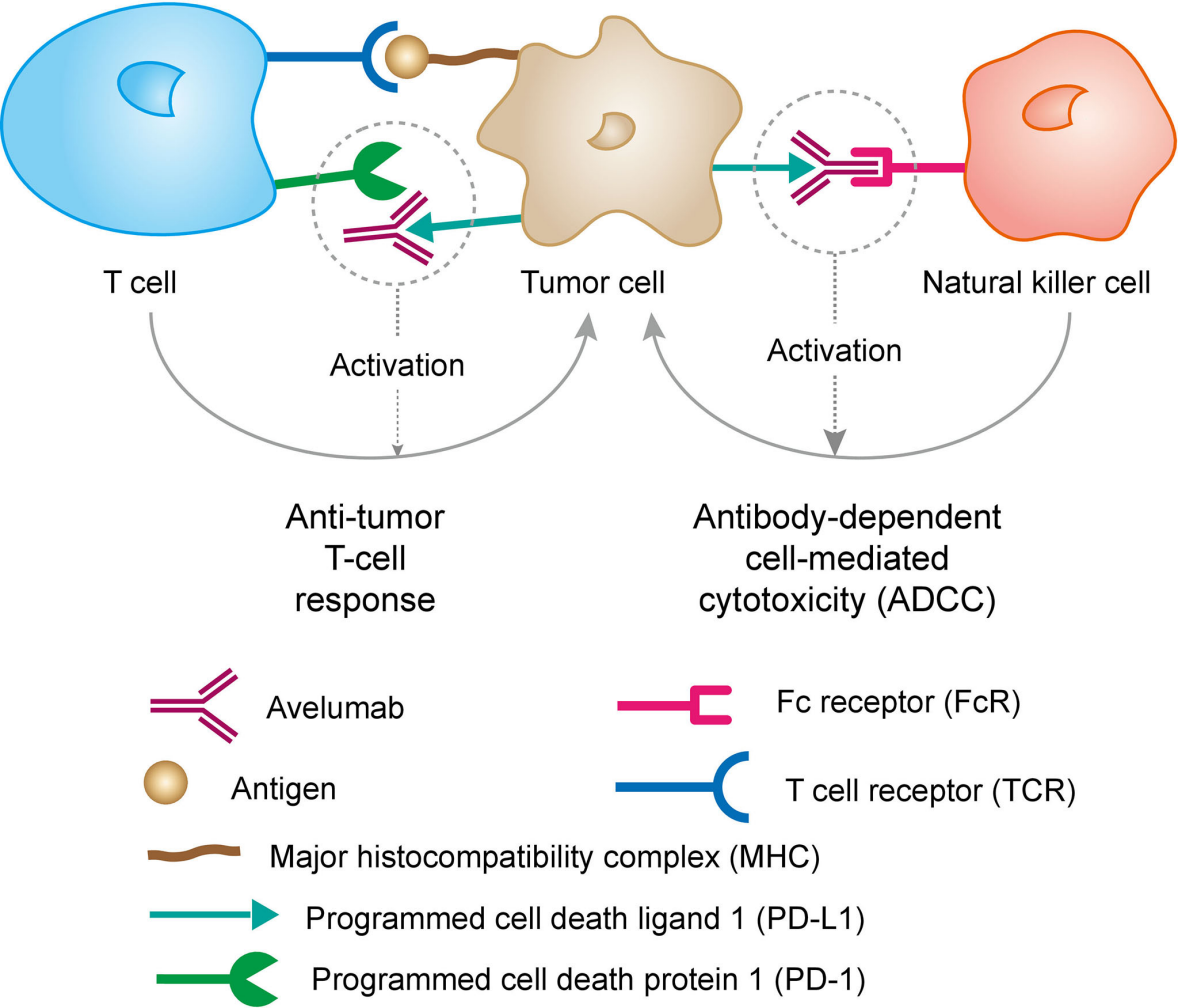
Mechanism of action of Avelumab. PD-L1 may be expressed on tumor cells and tumor-infiltrating immune cells and can contribute to the inhibition of the anti-tumor immune response in the tumor microenvironment. Binding of PD-L1 to the receptors PD-1 and B7.1 found on T cells and antigen presenting cells suppresses cytotoxic T-cell activity, T-cell proliferation and cytokine production. Avelumab binds PD-L1 through the FG loops and blocks the interaction between PD-L1 and its receptors PD-1 and B7.1. This interaction releases the inhibitory effects of PD-L1 on the immune response resulting in the restoration of immune responses, including anti-tumor immune responses. Antibody-dependent cell-mediated cytotoxicity (ADCC) response has also been found to induced by avelumab. Reprinted with permission from “Avelumab Overview - Creative Biolabs”. J Clin Invest 2015; 125: 3377-3383. Copyright ^©^ 2007 - 2019 Creative-Biolabs All Rights Reserved.

Avelumab, a human IgG1 anti-PD-L1 immune-checkpoint blocker, which can competitively blocks the binding between PD-1 and PD-L1 without affecting PD-1/PD-L2 interactions. This mechanism of action not only can be achieved by promoting tumor infiltration and T lymphocytes to produce cytotoxic molecules (IL2 and IFNγ), but also potentially mediate Ab-dependent cell cytotoxicity (ADCC) against tumor cells. The mechanism of action of Avelumab is shown in **Figure 4**. To assess the activity of Avelumab in patients with metastatic breast cancer, Dirix et al. finished a phase 1 trial (JAVELIN Solid Tumor; NCT01772004) recently (62).

The study included 168 patients with MBC, including 58 patients with TNBC who underwent Avelumab for 2-50 weeks for 6-15 months. For patients with metastatic or locally advanced breast cancer, the investigators received a series of pretreatments. 13.7% of patients experienced ≥3 treatment-related side effects, including 2 treatment-related deaths. Overall, the objective response rate (ORR) was approximately 3.0% (1 of which was “complete response” and the other 4 were “partially reflected”), and the ORR for TNBC patients was 5.2%. In the overall population, PD-L1-positive patients had higher levels of tumor-associated immune cells (16.7% vs. 1.6%) compared with PD-L1-negative patients; in the TNBC subgroup, PD-L1-positive patients had higher ORR than in patients with negative PD-L1 (22.2% versus 2.6%). Therefore, Avelumab exhibits acceptable safety and clinical activity in a subset of patients with metastatic breast cancer. The expression of PD-L1 in cancer-associated immune cells may be associated with a higher probability of clinical response to Avelumab in metastatic breast cancer (62). In addition, La Rocca E et al. (63) reported the initial experience of a TNBC patient who received avirumumab combined with radiotherapy, and then found that there was no subacute pulmonary toxicity 12 weeks after the end of radiotherapy. Although this result is encouraging, the follow-up time is too short and the patient is still at risk of developing pulmonary fibrosis, and further large-scale studies are needed. Recently published studies on the applications of Avelumab in other types of cancers are shown in **Table 3** (64–70).

**Table 3:** Recent published studies on the application of Avelumab in various cancers.

Author	Year	Institution	Country	Journal	Cancer Type	Research Type	Rearch Purpose	Key Conclusions
Doi et al. (64)	2018	National Cancer Center Hospital East	Japan	Gastric Cancer	Advanced gastric cancer/gastroesophageal junction cancer.	Phase I	To assess safety in Japanese patients with advanced solid tumors and clinical activity in patients with advanced gastric cancer/gastroesophageal junction cancer and disease progression after chemotherapy.	Avelumab showed acceptable safety in Japanese patients with advanced solid tumors and clinical activity in patients with advanced gastric cancer/gastroesophageal junction cancer and disease progression after chemotherapy.
Yu Y et al. (65)	2018	Memorial Sloan Kettering Cancer Center	USA	Future Oncol	Locoregionally advanced squamous cell carcinomas of the head and neck (HNSCC)	Multinational, randomized Phase III,	Assessing the efficacy of avelumab, a PD-L1 inhibitor, in combination with CRT compared with placebo in combination with CRT for high-risk HNSCC.	Avelumab: 1) Fully human monoclonal IgG1 antibody against PD-L1; 2) Fc component intact, capable of inducing ADCC; 3) The US FDA approved for Merkel cell carcinoma and urothelial carcinoma; 4) Well tolerated across many solid tumor types.
Barlesi et al. (66)	2018	Aix Marseille University	France	Lancet Oncol	Non-small-cell lung cancer (NSCLC)	Multicentre, open-label, randomised, phase III trial	To investigate the efficacy and safety of avelumab, an anti-PD-L1 antibody, in patients with NSCLC who had already received platinum-based therapy.	Compared with docetaxel, avelumab did not improve overall survival in patients with platinum-treated PD-L1-positive NSCLC, but had a favourable safety profile.
Merlano et al. (67)	2018	Medical Oncology A.O. S.Croce e Carle	Italy	Clin Transl Radiat Oncol	Platinum-resistant relapsed/metastatic (R/M) head and neck cancer (HNC)	Open label, multi-center, single-arm, Phase Ib/II,	To test the hypothesis that attacking the tumor microenvironment at multiple levels can increase immunogenicity of R/M-HNC without worsening the safety profile of immune checkpoint inhibitors.	1) Two ongoing trials (NCT02318771, NCT02684253) in R/M-HNC combine RT at doses of 8 Gy, 4 Gy in five fractions, or 27 Gy in three fractions with anti-PD-1 ICIs. 2) The results of ongoing trials will better clarify the potential of immunotherapy in R/M-HNC patients. 3) Complementary multimodal immunotherapy has a strong rationale to counterbalance the established immunosuppression of R/M-HNC, and theoretically, the proposed combined treatment could improve the activity of avelumab without increasing its toxic profile.
Bang et al. (68)	2018	Seoul National University College of Medicine	South Korea	Ann Oncol	Advanced gastric cancer/gastro-oesophageal junction cancer (GC/GEJC)	Randomised, phase III	To compare avelumab versus physician's choice of chemotherapy as third-line therapy in patients with advanced GC/GEJC.	Treatment of patients with GC/GEJC with single-agent avelumab in the third-line setting did not result in an improvement in OS or PFS compared with chemotherapy. Avelumab showed a more manageable safety profile than chemotherapy.
Pujade-Lauraine et al. (69)	2018	Hôpital Hôtel-Dieu	France	Future Oncol	Ovarian cancer	Randomized Phase III trial	To evaluate the role of checkpoint inhibition in women with ovarian cancer.	The JAVELIN Ovarian 200 trial will demonstrate whether avelumab as monotherapy or in combination with chemotherapy can improve PFS or OS in patients with platinum-refractory/resistant disease compared with standard chemotherapy. It is hoped that this Phase III trial, along with others in progress, will lead to the availability of new treatment options that can improve outcomes for patients with EOC.
Gulley et al. (70)	2017	Genitourinary Malignancies Branch	USA	Lancet Oncol	Advanced, platinum-treated non-small-cell lung cancer (NSCLC)	Multicentre, open-label, phase I	To assess avelumab treatment in a cohort of patients with advanced, platinum-treated non-small-cell lung cancer (NSCLC).	Avelumab showed an acceptable safety profile and antitumour activity in patients with progressive or treatment-resistant NSCLC, providing a rationale for further studies of avelumab in this disease setting.

## Immunological Checkpoint Inhibitors Targeting LAG-3

Lymphocyte activation gene-3 (LAG-3, CD223), a CD4-like molecule, binds to major histocompatibility complex class II (MHC II) with higher affinity and is expressed by activated T cells, natural killers (NK) cells, B cells, and dendritic cells (DCs) (71). LAG-3 selectively upregulates CD4 on the surface of Treg, so LAG-3 antibody can reduce Treg activity *in vivo*, inhibition or knockout of LAG-3 will relieve the inhibitory function of Treg on T cells. In addition, in the absence of CD4+ T cells, LAG-3 antibodies can increase the function of CD8+ T cells, it also been found to be expressed in Treg cells to promote the production of inhibitory cytokines such as IL-10. the above description is the main mechanism of LAG-3 molecule suppressing immunity. Indeed, LAG-3 and PD-1 are commonly co-expressed on exhausted or dysfunctional T cells in models of chronic infections and cancers (72), the coordinated inhibition of LAG-3 and PD-1 can enhance the immune response, Therefore, most of the current clinical trials on LAG-3 antibodies are combined with PD-1 to observe the effect (73–75).

In an earlier study, Triebel et al. (76) collected 246 patient’s sera from patients diagnosed with breast cancer for the first time for a cohort study and found that disease-free and overall survival rates were greater in patients with estrogen or progesterone receptor positive tumor cells who had detectable levels of sLAG-3 at diagnosis versus patients with undetectable sLAG-3 levels. In additional, in a recent study by Burugu et al, immunohistochemistry on tissue microarrays from 4,322 breast cancer resection specimens demonstrated that the LAG-3+ iTILs was found to be associated with poor prognosis and its expression is correlated with PD-1/PD-L1 expression (77). Bottai et al. (78) studied 363 cases of triple-negative breast cancer and found that the number of iTILs is an independent factor influencing the prognosis of TNBC. they reported that PD-1 and LAG-3 are co-expressed in 15% of patients, and there is a positive correlation with the number of TILs, However, in contrast with the current study by Buruga et al, they did not observe a significant improvement in survival for LAG-3 positive cases. The difference between the results of each study may be the study of the small numbers of LAG-3/PD-1 positive cases, secondly, the study method may be different.

Although there are many clinical studies confirming that the benefits of anti-PD-1/anti-PD-L1 therapies have a certain clinical effect on advanced breast cancer, we can still see that a considerable proportion of patients have shown little clinical efficacy. LAG3 acts as the third immune checkpoint after PD-L1 and CTLA-4 and shown obvious advantages. In vitro and in animal models, we have seen that blocking PD-1/PD-L1 and LAG3 pathways at the same time is better than single blocking, which provides new space for immunotherapy for breast cancer.

## Immunological Checkpoint Inhibitors Targeting TIM-3

T cell immunoglobulin domain and mucin domain-3 (TIM-3), a member of the Tim family, which contains an immunoglobulin and a mucin-like domain and is expressed on the surface of T cells, Treg cells, and innate immune cells (dendritic cells, natural killer cells, monocytes) and characterized as a negative regulator for immune responses (78, 79). So far, at least four ligands for Tim-3 such as phosphatidylserine, galectin-9, HMGB1 and CEACAM-1 have been described (80). Unlike other immune checkpoint molecules, TIM-3 is only up-regulated in CD4+ helper T cell 1 (Th1) and CD8+ cytotoxic T cells and participates in synergistic inhibition. After being activated by its ligand galectin-9, TIM-3 inhibits the activity of effector T cells and leads to the reduction of effector cytokines and the apoptosis of effector T cells.

Many studies have shown the presence of Tim-3 in tumor-infiltrating lymphocytes (TIL) (81, 82). In breast cancer, only limited studies have reported the expression of TIM-3 in breast cancer (83–85). The level of Tim-3 is closely related to tumor-infiltrating CD8+ T cells, CD4+ T cells and DCs, especially the expression of Tim-3 on CD8+ T cells is higher than that in normal tissues (86). Heon EK et al. found that both interleukin-2 (IL-2) and interleukin-15 (IL-15) can be used as effective costimulatory signals to enhance breast cancer CD8+ T cell proliferation and interferon-γ (IFN -γ) Production. It proves that the Tim-3 signaling pathway is related to the apoptosis of CD8+T cells infiltrating tumors in breast cancer and the production of IFN-γ, and participates in the co-stimulation of IL-2 and IL-15 (87). At the same time, the immune effect of Tim-3 on non-T cells is also being studied. The expression of Tim-3 can promote the polarization of M2 macrophages and increase the secretion of IL-6. This experiment uses RAW264.7 cells to prove that STAT1 is the signal translator of Tim-3 in macrophages, and Tim-3 controls the polarization of macrophages by inhibiting the STAT1-miR-155 signal axis (88). In addition, at least in preclinical studies, TIM-3+ TILs can co-express PD-1, When the blocking effect of PD-1 antibody alone is not ideal, the combination with Tim-3 antibody to block the abnormally expressed PD-1 and Tim-3 on the surface of T cells can significantly increase the reversal of T-cell exhaustion (89–91). Drugs-based therapeutics targeting the inhibitory receptors PD-1, PD-L1, or CTLA-4 have shown remarkable clinical progress on several cancers, however, side effects and drug resistance have also appeared. Therefore, finding other targeted drugs for combined use is an urgent problem to be solved. At present, there is no approved marketed drug targeting the Tim-3 target at home and abroad, and its preclinical research and clinical trials are in progress. The combination strategy with PD-1/L1 drugs will also follow the clinical trials and Basic research is continuously optimized to better treat patients with drug resistance.

## Immunological Checkpoint Inhibitors Targeting TIGIT

T cell immunoglobulin and ITIM domain(TIGIT) containing Ig and ITIM domains (also known as VSIG9, VSTM3, and WUCAM) is a member of the CD28 family-like receptor of the immunoglobulin poliovirus receptor family (92). It is mainly expressed on subgroups of T cells(conventional αβ T cells, memory T cells, regulatory T cells, and NKT cells) and NK cells (93, 94). But under normal circumstances, its expression level is at a low level, its protein level will be up-regulated when these cells are activated. For example, in the tumor microenvironment, TIGIT in tumor-infiltrating lymphocytes is often at a high expression level (95). TIGIT can bind to the receptors CD155 (poliovirus receptor-PVR), CD112 (PVRL2, nectin-2), and CD113 (Nectin-3) in immune cells, non-immune cells, and tumor cells, resulting in T cell activation and suppression of cytotoxicity. CD155, a type I transmembrane glycoprotein, is a member of the immunoglobulin superfamily of cell adhesion molecules. CD155 and CD112 are mainly expressed on APCs, T cells, and tumor cells, etc. TIGIT competes with CD226 (DNAM-1) and CD96 (TACTILE) for corresponding ligands, and plays different roles: CD226 delivers a positive co-stimulatory signal, while CD96 and TIGIT deliver inhibitory signals (96). This group of proteins (TIGIT/CD155/CD226) interacts in a way similar to the CTLA-4/B7/CD28 costimulatory axis to regulate immune cell function. High expression of PVR(CD155 and CD112) could be associated with a poor survival in several cancer (97). In many preclinical models, the anti-tumor activity is studied by blocking the TIGIT/PVR axis. Therefore. the clinical evaluation of blocking TIGIT was initiated (NCT02794571, NCT03119428, NCT02913313). Randomized, double-blind and phase II CITYSCAPE trial (NCT03563716), which evaluated the efficacy and safety of the anti-TIGIT monoclonal antibody tiragolumab plus the anti-PD-L1 atezolizumab compared with atezolizumab alone as a first-line treatment for patients with PD-L1-positive NSCLC, showed a clinical benefit on the overall response rate (37% versus placebo 21%) and progression-free survival (5.5 months versus placebo 3.88 months) (98).

## Medical Predictor of Response to Checkpoint Inhibitors

Although immunotherapy brings new hope to patients with triple-negative breast cancer (TNBC) and advanced breast cancer. However, the overall response of programmed cell death protein-1 (PD-L1)/PD-ligand 1(PD-1) monoclonal antibody in breast cancer patients is not satisfied. Therefore, finding and screening the beneficial population among breast cancer patients through effective biomarkers is the key to a breakthrough in breast cancer immunotherapy. Currently, predictors for response to CPI are unclear, Biomarkers such as PD-L1 expression, tumor-infiltrating lymphocytes, tumor mutational burden (TMB) are potentially under investigation, although each of those has limitations itself. Herein, we will focus on PD-L1 and TIL.

### PD-L1 Expression

Tumor cells can inhibit the anti-tumor immunity of cytotoxic T cells through the expression of PD-L1, so PD-L1 expression is the first biomarker considered to predict the clinical efficacy of PD-1/PD-L1 inhibitors (99). Overexpression of PD-L1 is significantly related to the efficacy of PD-1/PD-L1 inhibitors in many tumor types, such as melanoma (44, 100), NSCLC (100), renal cell carcinoma, ovarian cancer. However, the expression level of PD-L1 has a big difference in the prediction of the efficacy of ICI drugs in breast cancer. In clinical trials of ICI drugs for TNBC, the positive rate of PD-L1 expression status in tumor cells or immune cells ranged from 19.4% to 68%, and the overall response rate to ICI drugs was 5% to 42% (62, 101–106). And it was found that patients with negative PD-L1 expression may also respond to ICI drugs (100). The inconsistency of the above research results suggests that the applicability of PD-L1 expression as a biomarker for efficacy prediction still needs further discussion and analysis. To this end, we analyzed the difficulties and challenges of PD-L1 as a biomarker for efficacy prediction.

The spatial heterogeneity of PD-L1 expression exists both within the same tumor lesion, and between primary and different metastatic lesions in the same patient. Cimino-Mathews A et al. described a situation in which a patient with triple-negative breast cancer was negative for PD-L1 expression in the primary tumor, but was positive in lung metastases (107). Li, Ming et al. (108) analyzed PD-L1 expression in 101 TNBC patients using a cutoff value of PD-L1>=5%, the positive rate of PD-L1 expression in the primary tumor tissue was 38.6%, The positive rate of PD-L1 expression in the tissue of axillary lymph node metastasis is 59.4%. In a study including 245 primary and 40 metastatic (20 nodal, 20 distant) breast carcinomas using a cutoff value of PD-L1>=1%. Tumor PD-L1 staining was seen in 12% of all primaries including 32% of triple-negative cancers. Staining was common in ductal cancers with medullary (54%), apocrine (27%), and metaplastic features (40%). Tumor cell expression of PD-L1 was observed in 10% (2/20) of nodal and 10% (2/20) of distant metastases. These data demonstrate that PD-L1 expression has considerable intratumoral heterogeneity (109). At present, there is no further research on the heterogeneity mechanism of PD-L1 expression. However, considering the heterogeneity of PD-L1 expression, tumour sampling at one time point or at only one tumour site or a portion of one tumour may not accurately reflect the overall situation of PD-L1 expression.

In addition, inconsistencies in the antibody clones, cell types scored and positivity cutoffs are another main reason. Currently, the US Food and Drug Administration (FDA) has approved four unique PD-L1 antibody clones, including Dako 28-8, Dako 22C3, Ventana SP142, and Ventana SP263. The Blueprint project compared the above four PD-L1 assays and found that similar performance on tumor cells staining in three assays (22C3, 28-8, and SP263) and fewer tumor cells staining in the SP142 assay, with low concordance rates in the scoring of immune cells among the four assays (110). For example, the IMpassion130 study used Ventana SP142 assay to detect PD-L1 expression on tumor cells and immune cells, using the threshold cutoff of more than 1%, and found that the prevalence of PD-L1 positive tumors was 40% (59). However, in another study, using the same cut-off value for the SP142 assay, Reisenbichler, ES et al. found the PD-L1 positive rate was 58% (111). At the same time, they found that the prevalence of PD-L1 positive tumors can reach 60% by SP263 assay (111). which showed that the sensitivity of SP142 to detect PD-L1 protein is lower than other detection methods and substantially lower reproducibility. Nevertheless, PD-L1 expression >=1% on immune cells (by SP142 Ventana assay) has been approved by both FDA (Food and Drug Administration) and EMA (European Medicines Agency) based on the positive results of the Impassion130 randomized trial. With such an outcome, we express our concern that the use of this assay by different institutions in the clinic may expose patients with false positive results to expensive and potentially toxic treatments.

At present, The definition of PD-L1 positive lacks standardization, and prediction of response by IHC analysis is additionally limited by the subjective nature of the technique. the quantitative studies on IHC analysis of a variety of PD-L1 with different antibodies and cut points are few. Martinez-Morilla S et al. (112) used a standardized cell line tissue microarray for quantitative assessment of PD-L1, allowing the comparison of the PD-L1 assays across both time and institution, and found that differences in PD-L1 expression in tissue are independent of the antibody itself used and likely attributable to tumor heterogeneity, assay or platform-specific variables, or other factors. Simultaneously, they found that the sensitivity of the SP142 detection method was lower than the other three FDA-approved detection methods (112). Such results are consistent with previous studies (113). In general, there is currently no gold standard for PD-L1 quantitative assessment, and standardized and universally applicable detection methods still require more clinical data analysis and accumulation.

So far, a single biomarker has basically not been able to satisfy analytical validity, robustness, reproducibility and clinical utility at the same time. At the same time, it must also be affordable and used in academic and community hospital practice around the world. Pathologists can use it. Evaluation of composite biomarkers may be the best way to identify patients most likely to respond to ICI, such as a combination of TIL and PD-L1 (114). In addition, in the face of the dilemma of implementation of PD-L1 assays in clinical trials and daily practice requires, as guardians of patient samples, pathologists must cooperate with clinicians, industry and regulatory agencies to guide evidence-based biology in clinical trials and daily practice to ensure the best patient outcomes possible (115).

### Tumor-Infiltrating Lymphocytes

Tumor-infiltrating lymphocytes (TIL) is a heterogeneous group of lymphocytes that exist in tumor nests and interstitium. The predictive effect of TIL on breast cancer immunotherapy is related to the type of TIL, the molecular type of breast cancer and the stage of development. A meta-analysis that included 25 articles with 22964 patients on the expression of TIL in different molecular subtypes of breast cancer showed that tumor-infiltrating lymphocytes were not correlated with the disease-free survival and overall survival of the entire breast cancer population, but in triple-negative breast cancer, With the increase of tumor-infiltrating lymphocytes, disease-free survival and overall survival have been prolonged (116). At the same time, they also found that the CD8+ TILs was positively correlated with the prolongation of DFS, while the FoxP3+ TILs subgroup was negatively correlated with DFS (116). In addition, Denkert, Carsten et al. performed a pooled analysis of 3771 breast cancer patients and found that TILs are expressed higher in TNBC and HER2-positive breast cancer patients (30% and 19%) than other subtypes (117).

Recent studies have shown that TILs have shown a strong prognostic effect in breast cancer patients treated with neoadjuvant or adjuvant therapy(chemotherapy, Targeted therapy). High levels of iTILs before neoadjuvant chemotherapy (NAC) is associated with a high pCR rate (117, 118). even for patients who have not reached pCR, High TILs in residual disease (RD) have been shown to correlate with favorable prognosis (119, 120). In a FinHER trial, Loi S et al. (121) studied the predictive effect of TIL on trastuzumab sensitivity in HER2-positive early breast cancer patients and found that the risk of distant metastasis of tumors decreased with the increase of interstitial TIL. Furthermore, the N9831 trial conducted by Perez et al. showed that immune-rich tumors defined at the gene level, that is, tumors with high TIL infiltration, have better prognosis after receiving trastuzumab treatment. Although it has not been found that the use of trastuzumab in patients with HER2-positive breast cancer is affected by the infiltration of TIL, TIL may be related to the potential mechanism of trastuzumab (122).

Tumor infiltrating immune cells play an important role in predicting the sensitivity of breast cancer immunotherapy and have become a recent research hotspot. In a clinical trial of IMassion130 (59), sTIL+ patients had longer PFS and OS with atezolizumab + nab-paclitaxel. However, this benefit is only when the tumor is also PD-L1 IC+ at the same time, Improved PFS or OS outcomes were not observed in patients negative for both biomarkers (PD-L1 IC and sTILs) (123). In a phase II trial (KEYNOTE-086) (51), High stromal TIL levels were associated with improved ORR in patients with metastatic TNBC receiving pembrolizumab (PD-1 inhibitor).These findings are similar to those of the phase III KEYNOTE-119 trial (124), which may indicate TILs are emerging as potentially important biomarkers of prediction of response to immunotherapeutic agents in breast cancer.

Compared to PD-L1, TILs can be assessed on optical microscopes or simple hematoxylin and eosin(H&E) slides by using digital techniques. Moreover, the pathologist can use the same H&E slide for diagnosis, so that it does not take too much time and effort for the pathologist. Secondly, tumor-infiltrating lymphocyte assessment has reliable reproducibility among pathologists for core biopsies and complete sections. Although there is a detailed set of visual standard evaluation methods developed by the International TIL Working Group that uses histological methods to evaluate the percentage of TIL in primary tumor specimens (6), TILs are not a single type of cell. How to standardize the composition ratio of tumor-infiltrating lymphocytes with different degrees of infiltration and various cells within tumor-infiltrating lymphocytes is the future directions that needs further research and analysis.

Of course, in addition to PD-L1 and TIL, potential biomarkers such as tumor mutational burden (TMB), microsatellite instability (MSI) and immune-related genes, have also shown themselves in breast cancer immunotherapy. The combination of these markers will be able to more accurately and effectively screen the population who may benefit from immunotherapy.

## Side Effects of Checkpoint Inhibitors for Breast Cancer

Immune checkpoint inhibitors (ICI) targeting programmed cell death-1 (PD-1) or cytotoxic T lymphocyte antigen-4 (CTLA-4) pathways have made great progress in breast cancer treatment. However, the application of ICIs destroys the mechanism that might protect tissues from autoimmune response damage, enhances the activity of T cells and cause them to attack normal tissue cells, and increases the level of pre-existing autoantibodies and inflammatory factors, leading to various types of immune-related adverse events (irAEs) and treatment- related adverse events (TRAEs) (125). Here we focus on irAEs, which have inflammatory or autoimmune properties and can occur at any time during treatment, even after ICIs treatment is stopped (126). it may affect any number of organ systems including the gastrointestinal tract (colitis, diarrhea), the lung(pneumonitis), the endocrine system (hypothyroidism, adrenal insufficiency, hypophysitis and/or type 1 diabetes mellitus), the liver(hepatitis), the skin (rash, pruritus), and rare immunotherapy-related toxicity (nervous system toxicity, cardiotoxicity, ocular toxicity, nephrotoxicity) (127). Chang LS et al. found that most irAEs are reversible, and the effects of the endocrine system may be permanent (128).

In a systematic review, Michot et al. found that the incidence of IRAE in patients receiving anti-CTLA-4 antibody treatment was as high as 90% and 70% of patients treated with a PD-1/PD-L1 antibody, AEs with grade ≥ 3 were respectively 14% and 34%. Moreover, AEs with grade 1-2 mainly affected the skin and the gut, whereas AEs with grade 3-4 were mainly restricted to the digestive system (129). Among breast cancer, a systematic review and meta-analysis including nine studies with 4687 patients conducted by Balibegloo M reported that the overall frequency of irAEs was 35.7%. among which rash and infusion reaction were the two most frequent irAEs of any grade and grade 3-5(37.8%, 12.08%) (130). Similarly, D’Abreo N et al. (131) reviewed 8 published breast cancer ICI trials and found that the most common IRAEs were skin rash and itching (up to 18%), thyroid disease (up to 12%) and abnormal liver function (up to 10%).This immune-related cutaneous adverse events (irCAEs) are usually self-limiting. The treatment algorithm of it mainly revolves around early identification and use of corticosteroids or anti-tumor necrosis factor-a drugs (132). In additional, in a phase 1b clinical trial with atezolizumab plus nab- paclitaxel in the treatment of metastatic triple- negative breast cancer, Adams S et al. reported that the incidence of immune-related pneumonia is 9% (133), which is higher than other related studies (130, 134). The main reason we are concerned about pneumonia is that taxanes can cause overlapping pulmonary toxicity. Secondly, there have been fatal pulmonary toxicity in patients in previous studies (62, 135). Of course, in other studies, other adverse events were also observed, such as colitis or diarrhea (2%), insufficiency is infrequent (≤1%), hypothyroidism (12.22%) and hepatitis (94%) (37, 130). AlthoughrAEhese irae may lead to patient death, it is not common and occurs in <1.5% of cases with irAEs (136). However. fatal adverse events have been reported with ICIs (137), especially for cardiovascular and neurologic irAEstoxicities (138), need cautious monitorings, urgently investigating and careful managements. Different from the anti-tumor mechanism of traditional cytotoxic drugs, ICIs-related adverse events form a unique disease spectrum of irAEs. Overall, the incidence and severity of irAEs are lower than those of chemotherapy, secondly, most irAEs are controllable and reversible, and fatal irAEs are rare. Therefore, early identification and timely treatment of irAEs are essential to prevent serious and/or permanent sequelae.

## Conclusion

The application of immune checkpoint inhibitors has ushered in a new era of TNBC treatment. We have high hopes for this, but at the same time, we must be soberly aware that there are still many unanswered questions. How to explore and definition of a more standardized and comprehensive approach for the detection of PD-L1 on both tumor cells and immune cells, how to screen patients who may benefit greatly but have less immune-related adverse reactions, how to individualize and optimize treatment, etc. These are all problems that need to be solved in the future. A deeper understanding of the complex interactions between breast cancer and the immune system helps to explore more effective immunotherapy options for breast cancer, which will usher in a new breakthrough in breast cancer treatment.

## Author Contributions

WZ contributed to the study design, data acquisition, data analysis and interpretation, manuscript writing and revision. XK and BA contributed to the study data acquisition, data analysis and interpretation, manuscript revision. ZW, XW and NW contributed to the data acquisition and analysis. SZ contributed to the study data analysis, YF contributed to review and revise the manuscript. JW contributed to the study conception and design, review and revise the manuscript. All authors contributed to the article and approved the submitted version.

## Funding

This work was supported by the Natural Science Foundation of China (No. 81872160), the Natural Science Foundation of China (No. 82072940), the China National Key R&D (or Research and Development) Program (No. 2020AAA0105000 and 2020AAA0105004), the Beijing Municipal Natural Science Foundation (Key Project) (No. 7191009), the Beijing Municipal Natural Science Foundation (No. 7204293), the Special Research Fund for Central Universities, Peking Union Medical College (No. 3332019053), the Beijing Hope Run Special Fund of Cancer Foundation of China (No. LC2019B03), the Beijing Hope Run Special Fund of Cancer Foundation of China (No. LC2019L07), the Beijing Hope Run Special Fund of Cancer Foundation of China (No. LC2020L01), the Golden Bridge Project Seed Fund of Beijing Association for Science and Technology (No. ZZ20004), the 2021 Chaoyang District Social Development Science and Technology Plan Project (Medical and Health Field) (No. CYSF2115), the Chinese Young Breast Experts Research project (No. CYBER-2021-005), the XianSheng Clinical Research Special Fund of China International Medical Foundation (No. Z-2014-06-2103), and the Beijing Xisike Clinical Oncology Research Foundation (No. Y-Young2021-0017).

## Conflict of Interest

The authors declare that the research was conducted in the absence of any commercial or financial relationships that could be construed as a potential conflict of interest.

## Publisher’s Note

All claims expressed in this article are solely those of the authors and do not necessarily represent those of their affiliated organizations, or those of the publisher, the editors and the reviewers. Any product that may be evaluated in this article, or claim that may be made by its manufacturer, is not guaranteed or endorsed by the publisher.

## Supplementary Material

Supplementary Table 1 Some ongoing clinical trials of anti-CTLA-4 immunotherapeutic interventions of breast cancer.

Supplementary Table 2 Some ongoing clinical trials of anti-PD-1 immunotherapeutic interventions of malignancies including breast cancer.

Supplementary Table 3 Some ongoing clinical trials of anti-PD-L1 immunotherapeutic interventions of malignancies including breast cancer.
