# Supplementary material for: Research Progresses in Immunological Checkpoint Inhibitors for Breast Cancer Immunotherapy
Source: Front Oncol. 2021 Sep 23;11:582664. doi: 10.3389/fonc.2021.582664 (PMC8495193; doi:10.3389/fonc.2021.582664)
Supplement: Supplementary Table 1 — Some ongoing clinical trials of anti-CTLA-4 immunotherapeutic interventions of breast cancer. [file Table_1.doc]

| **Supplementary table 1,** **Some ongoing clinical trials of anti-CTLA-4 immunotherapeutic interventions of breast cancer** | | | | | | | | | | | | | |
| --- | --- | --- | --- | --- | --- | --- | --- | --- | --- | --- | --- | --- | --- |
| **Drug** | **NCT Number** | **Title** | **Status** | **Conditions** | **Interventions** | **Characteristics** | | | | **Population** | | | **Sponsor/**  **Collaborators** |
|  |  |  |  |  |  | **Study Type** | **Phase** | **Study Design** | **Outcome Measures** | **Enrollment** | **Age** | **Sex** |  |
| Ipilimumab | NCT03546686 | Peri-Operative Ipilimumab+Nivolumab and Cryoablation Versus Standard Care in Women With Triple-negative Breast Cancer | Not yet Recruiting | Breast Cancer | •Drug: Ipilimumab  •Drug: Nivolumab  •Procedure: Core Biopsy/Cryoablation  •Procedure: Breast Surgery | Interventional | Phase 2 | •Allocation: randomized  •Intervention Model: Parallel Assignment  •Masking: None (Open Label)  •Primary Purpose: Treatment | •Primary outcome measure:  •Distant Disease-Free Survival  •Invasive Disease-Free Survival  •Disease-Free Survival  •Overall Survival  •Overall Safety | 150 | 18 Years and older (Adult, Older Adult) | Female | •Cedars-Sinal Medical Center |
| Ipilimumab | NCT03409198 | Phase IIb Study Evaluating Immunogenic Chemotherapy Combined With Ipilimumab and Nivolumab in Breast Cancer (ICON) | Recruiting | •Breast Cancer  •Luminal B | •Drug: Ipilimumab  •Drug: Nivolumab  •Drug: Pegylated  liposomal  doxorubicin  •Drug:  Cyclophosphamide | Interventional | Phase 2 | Study Design:  •Allocation: Randomized  •Intervention Model: Parallel  Assignment  •Masking: None (Open  Label)  •Primary Purpose:  Treatment | •Toxicity: CTCAE v4.0  •Progression-free survival  (PFS)  •Duration of Response (DR)  •Overall Survival (OS)  •Duration of Response (DR) in cross-over arm  •Overall Suvival (OS) in  cross-over arm  •Toxicity, cross-over arm,  CTCAE v4.0  •Objective tumor Response  Rate (ORR)  •Durable tumor Response  Rate (DRR)  •Objective tumor Response  Rate (ORR) in cross-over  arm  •and 7 more | 75 | 18 Years and older (Adult, Older Adult) | All | •Oslo University Hospital  •Bristol-Myers  Squibb  •Helse Stavanger HF  •Helse Sor-Ost  •Sorlandet  Hospital HF |
| Ipilimumab | NCT03650894 | Nivolumab, Ipilimumab, and Bicalutamide in Human Epidermal Growth Factor (HER) 2 Negative Breast Cancer Patients | Not yet Recruiting | •Breast Neoplasm Female  •Breast Cancer  •Breast Carcinoma  •Breast Tumor | •Drug: Nivolumab  •Drug: Ipilimumab  •Drug: Bicalutamide | Interventional | Phase 2 | •Intervention Model: Single Group Assignment  •Masking: None (Open Label)  •Primary Purpose: Treatment | •iRECIST Clinical Benefit Rate (the number of patients with objective  response or ongoing stable disease at week 24 using iRECIST guidelines)  •RECIST Clinical Benefit Rate (the number of patients who have a response according to RECIST criteria at week 12) | 138 | 18 Years and older (Adult, Older Adult) | Female | •Providence Health &Services •Bristol-Myers Squibb •Memorial Sloan Kettering Cancer Center |
| Ipilimumab | NCT02892734 | Ipilimumab and Nivolumab in Treating Patients With Recurrent Stage IV HER2 Negative Inflammatory Breast Cancer | Recruiting | •HER2/Neu Negative  •Recurrent Inflammatory Breast Carcinoma  •Stage IV Breast Cancer  •Stage IV Inflammatory Breast Carcinoma | •Biological: Ipilimumab  •Other: Laboratory Biomarker Analysis  •Biological: Nivolumab | Interventional | Phase 2 | •Intervention Model: Single Group Assignment  •Masking: None (Open Label)  •Primary Purpose: Treatment | •Progression Free Survival (PFS)  •Overall Response Rate (ORR)  •Clinical Benefit Rate (CBR)  •Overall survival  •Incidence of Adverse Events | 29 | 18 Years and older (Adult, Older Adult) | Female | •Northwestern  University  •Bristol-Myers  Squibb  •National Cancer  Institute (NCI) |
| Ipilimumab | NCT03342417 | Combination of Nivolumab and Ipilimumab in Breast, Ovarian and Gastric Cancer Patients | Recruiting | •Breast Cancer Female  •Ovarian Cancer  •Gastric Cancer | •Biological:  Nivolumab  •Biological:  Ipilimumab | Interventional | Phase 2 | •Allocation: NonRandomized  •Intervention Model: Single Group Assignment  •Masking: None (Open Label)  •Primary Purpose: Treatment | •Number of participants with treatment-related adverse events as assessed by CTCAE v4.0  •Clinical Response  •Part 1 of the Study (Neoadjuvant Therapy of Breast Cancer) - BCT  •Part 1 of the Study (Neoadjuvant Therapy of Breast Cancer) – Possible predictive biomarkers  •Part 1 of the Study (Neoadjuvant Therapy of Breast Cancer) - Status of PD-L1, PDL-2, and PD-1 in tumor tissues before vs. after investigational neoadjuvant therapy  •Part 1 of the Study (Neoadjuvant Therapy of Breast Cancer) -Possible transition of Th2 to Th1 in  the tumor  •Part 1 of the Study (Neoadjuvant Therapy of Breast Cancer) - BOR and ORR  •Part 1 of the Study (Neoadjuvant Therapy of Breast Cancer) - PFS  •Part 2 of the Study (Therapy of Ovarian Cancer) and Part 3 of the  Study (Therapy of Gastric Cancer) - DOR  •Part 2 of the Study (Therapy of Ovarian Cancer) and Part 3 of the  Study (Therapy of Gastric Cancer) - OS  •Part 2 of the Study (Therapy of Ovarian | 60 | 18 Years and older (Adult, Older Adult) | All | •ExcellaBio LLC |
| Ipilimumab | NCT02453620 | Entinostat, Nivolumab, and Ipilimumab in Treating Patients With Solid Tumors That Are Metastatic or Cannot Be  Removed by Surgery or Locally Advanced or Metastatic HER2-Negative Breast Cancer | Recruiting | •Breast Adenocarcinoma  •HER2/Neu Negative  •Invasive Breast Carcinoma  •Metastatic Malignant Solid Neoplasm  •Stage III Breast Cancer AJCC v7  •Stage IIIA Breast Cancer AJCC v7  •Stage IIIB Breast Cancer AJCC v7  •Stage IIIC Breast Cancer AJCC v7  •Stage IV Breast Cancer AJCC v6  and v7  •Unresectable Solid  Neoplasm | •Drug: Entinostat  •Biological: Ipilimumab  •Other: Laboratory Biomarker Analysis  •Biological: Nivolumab  •Other: Pharmacogenomic  Study  •Other: Pharmacological Study | Interventional | Phase 1 | •Intervention Model: Single Group Assignment  •Masking: None (Open Label)  •Primary Purpose: Treatment | •Incidence of adverse events of entinostat and nivolumab in combination with ipilimumab per National Cancer Institute Common Terminology Criteria for Adverse Events (CTCAE) version (v)5.0  •Changes in ratio of effector T cell (Teff) to regulatory T cell (Treg) in tumor biopsies, measured by immunohistochemistry (IHC) staining of paraffin embedded tumor specimens  •Objective response rate, defined as the total number of patients with either complete response (CR) or partial response (PR) divided by the total number of patients in the population of interest (expansion cohort of  patients with advanced breast cancer)  •Disease control rate (expansion cohort of patients with advanced  breast cancer)  •Progression-free survival (PFS), defined as the proportion of patients remaining alive and free of disease progression (expansion cohort of patients with advanced  breast cancer)  •Duration of overall response (expansion cohort of patients with  advanced breast cancer)  •Duration of stable disease based on Response Evaluation Criteria in  (RECIST) version (v)1.1 and immune Solid Tumors (RECIST) version (v)1.1 and immune | 45 | 18 Years and older (Adult, Older Adult) | All | •National Cancer Institute (NCI) |
| Ipilimumab | NCT03328026 | Combination Study of SVBR-1-GM in Combination With  Pembrolizumab | Recruiting | •Breast Cancer Female  •Breast Neoplasm  Female | •Biological: SVBR-1-GM  •Biological: Pembrolizumab  •Drug: Low dose cyclophosphamide  •Biological: Interferon Inoculation | Interventional | •Phase 1  •Phase 2 | •Intervention Model: Single Group Assignment  •Masking: None (Open Label)  •Primary Purpose: Treatment | •Evaluate the Safety of SV-BR-1-GM (Adverse Events) when administered in combination with  ipilimumab (for patients with PD-L1/2- tumors) or pembrolizumab (for patients with PD-L1/2+ tumors) [Safety]  •Evaluate the Safety of SV-BR-1-GM (Laboratory Parameters)  when administered in combination with ipilimumab (for patients with PD-L1/2- tumors) or pembrolizumab (for patients with PD-L1/2+ tumors) [Safety]  •Evaluate the tumor response to SV-BR-1-GM (ORR) when administered in combination with  ipilimumab (for patients with PD-L1/2- tumors) or pembrolizumab (for patients with PD L1/2+ tumors)  •Evaluate the tumor response to SV-BR-1- GM (Non-progression) when administered in combination with ipilimumab (for patients with PD-L1/2- tumors) or pembrolizumab (for patients with PD L1/2+ tumors)  •Evaluate the tumor response to SV-BR-1-GM (Durability of response) when administered in combination with ipilimumab (for patients with PD-L1/2- tumors) or pembrolizumab (for patients with PD L1/2+ | 40 | 18 Years and older (Adult, Older Adult) | Female | •BriaCell Therapeutics Corporation  •Cancer Insight, LLC |
| Ipilimumab | NCT02983045 | A Dose Escalation and Cohort Expansion Study of CD122- Biased Cytokine (NKTR-214) in Combination With Anti-PD-1 Antibody (Nivolumab) or in Combination With Nivolumab and Anti-CTLA4 Antibody (Ipilimumab) in Patients With Select Advanced or Metastatic  Solid Tumors | Recruiting | •Melanoma  •Renal Cell Carcinoma  •Non Small Cell Lung Cancer  •Urothelial Carcinoma  •Triple Negative Breast Cancer | •Drug: Combination of NKTR-214 + nivolumab  •Drug: Combination of NKTR-214 + nivolumab + ipilimumab | Interventional | •Phase 1  •Phase 2 | •Allocation: NonRandomized  •Intervention Model: Parallel Assignment  •Masking: None (Open Label)  •Primary Purpose: Treatment | •Safety of NKTR-214 in combination with nivolumab as evaluated by incidence of drugrelated Adverse Events (AEs), Serious Adverse Events (SAEs), and adverse events leading to discontinuation, deaths, and clinical laboratory test abnormalities  •Safety of NKTR-214 in combination with nivolumab and ipilmumab as evaluated by incidence of drug-related AEs, SAEs, and adverse events leading to discontinuation, deaths, and clinical laboratory test abnormalities  •Tolerability of NKTR-214 in combination with nivolumab as evaluated by incidence of Dose Limiting Toxicities (DLTs), drug-related AEs, SAEs, adverse events leading to discontinuation, deaths and clinical laboratory test abnormalities  •Tolerability of NKTR-214 in combination with nivolumab and ipilmumab as evaluated by incidence of Dose Limiting Toxicities (DLTs), drug-related AEs, SAEs, adverse events leading to discontinuation, deaths and clinical laboratory test abnormalities  •Efficacy of NKTR-214 in combination with nivolumab as assessed by | 480 | 18 Years and older (Adult, Older Adult) | All | •Nektar Therapeutics  •Bristol-Myers  Squibb |
| Ipilimumab | NCT02834013 | Nivolumab and Ipilimumab in Treating Patients With Rare Tumors | Recruiting | •Acinar Cell Carcinoma  •Adenoid Cystic Carcinoma  •Adrenal Cortex Carcinoma  •Adrenal Gland Pheochromocytoma  •Anal Canal Neuroendocrine Carcinoma  •Anal Canal Undifferentiated Carcinoma  •Appendix Mucinous Adenocarcinoma  •Bartholin Gland Transitional Cell Carcinoma  •Bladder Adenocarcinoma  •Cervical Adenocarcinoma  •and 75 more | •Procedure: Biospecimen Collection  •Biological: Ipilimumab  •Biological: Nivolumab | Interventional | Phase 2 | •Intervention Model: Single Group Assignment  •Masking: None (Open Label)  •Primary Purpose: Treatment | •Overall response rate (ORR) defined as confirmed and unconfirmed complete and partial  response  •Incidence of adverse events graded by National Cancer Institute (NCI) Common Terminology Criteria for Adverse Events (CTCAE) version 4.0  •Best response calculated from the sequence of RECIST 1.1 and immunerelated response criteria (irRC) objectives  •Clinical benefit rate defined as complete response, partial response, or stable disease, estimated using both RECIST and irRC  •Overall survival (OS), estimated using both RECIST and irRC  •Progression free survival (PFS), estimated using both RECIST and irRC | 707 | 18 Years and older (Adult, Older Adult) | All | •National Cancer Institute (NCI) |
| Ipilimumab | NCT03126110 | Phase 1/2 Study Exploring the Safety, Tolerability, and Efficacy of INCAGN01876 Combined With Immune Therapies in Advanced or Metastatic  Malignancies | Recruiting | •Advanced Malignancies  •Metastatic Cancer | •Drug: INCAGN01876  •Drug: Nivolumab  •Drug: Ipilimumab | Interventional | •Phase 1  •Phase 2 | •Allocation: NonRandomized  •Intervention Model: Parallel Assignment  •Masking: None (Open Label)  •Primary Purpose: Treatment | •1. Phase 1: Safety and tolerability assessed by monitoring frequency, duration, and severity of adverse events (AEs)  •Phase 2: Objective response rate (ORR) based on Response Evaluation Criteria in Solid Tumors (RECIST) v1.1.  •Phase 1: ORR based on RECIST v1.1 and modified RECIST v1.1 (mRECIST v1.1)  •Phase 1 & Phase 2: Duration of response based on RECIST v1.1 and mRECIST v1.1  •Phase 1 & Phase 2: Duration of disease control based on RECIST v1.1 and mRECIST v1.1  •Phase 1 & Phase 2: Progression-free survival based on RECIST v1.1  and mRECIST v1.1  •Phase 1 & Phase 2: Overall survival  •Phase 2: Safety and tolerability assessed by monitoring frequency, duration, and severity of adverse events | 285 | 18 Years and older (Adult, Older Adult) | All | •Incyte Biosciences  International Sàrl  •Incyte Corporation |
| Tremelimumab | NCT03132467 | Evaluating Anti-PD-L1 Antibody (Durvalumab) Plus Anti-CTLA-4 Antibody (Tremelimumab) in HR+/HER2- Breast Cancer | Recruiting | •Breast Cancer  •Hormone Receptor Positive, HER Negative Breast Cancer | •Drug: Tremelimumab  •Drug: Durvalumab  •Procedure: Core Breast Tumor Biopsy | Interventional | Early Phase 1 | •Intervention Model: Single Group Assignment  •Masking: None (Open Label)  •Primary Purpose: Treatment | •Feasibility of Enrolling Participants with Hormone Receptor Positive (HR+)/Human Epidermal Growth Factor Receptor negative (HER2-) Breast Cancer to Trial with Durvalumab Plus Tremelimumab  •Safety of Tremelimumab plus Durvalumab in Participants with Early Stage HR+/HER2- Breast Cancer: Adverse events recorded according to CTCAE 4.03 | 15 | 18 Years and older (Adult, Older Adult) | Female | •M.D. Anderson Cancer Center  •AstraZeneca |
| Tremelimumab | NCT03608865 | Durvalumab (MEDI4736) and Tremelimumab in Hormone Receptor-positive, Hypermutated Metastatic Breast Cancer Identified by Whole Exome Sequencing | Recruiting | •Metastatic Breast Cancer | •Drug: Durvalumab with Tremelimumab | Interventional | Phase 2 | •Intervention Model: Single Group Assignment  •Masking: None (Open Label)  •Primary Purpose: Treatment | •Objective response rate (ORR) by RECIST 1.1  •Clinical benefit rate (CBR) by RECIST 1.1 defined by complete or partial response or stable disease  for at least 24 weeks  •Duration of response (DoR)  •Disease control rate (DCR) by RECIST 1.1  •Progression-free survival (PFS) by RECIST 1.1 | 30 | 19 Years and older (Adult, Older Adult) | Female | •Yonsei University |
| Tremelimumab | NCT02536794 | MEDI4736 and Tremelimumab in Treating Patients With Metastatic HER2 Negative Breast Cancer | Recruiting | •Estrogen Receptor  Negative  •Estrogen Receptor  Positive  •HER2/Neu Negative  •Recurrent Breast Carcinoma  •Stage IV Breast Cancer | •Biological: AntiB7H1 Monoclonal Antibody MEDI4736  •Other: Laboratory Biomarker Analysis  •Other: Pharmacological Study  •Biological: Tremelimumab | Interventional | Phase 2 | •Intervention Model: Single Group Assignment  •Masking: None (Open Label)  •Primary Purpose: Treatment | •Response rate of MEDI4736 in combination with Tremelimumab  •Toxicity of MEDI4736 in combination with Tremelimumab  •Overall Survival (OS)  •Progression Free Survival (PFS) | 15 | 18 Years and older (Adult, Older Adult) | Female | •M.D. Anderson Cancer Center  •AstraZeneca |
| Tremelimumab | NCT03430466 | Anti PD-L1 Antibody + Anti CTLA-4 Antibody in Combination With Hormone Therapy in Patients With Hormone  Receptor Positive HER2- negative Recurrent or Metastatic  Breast Cancer | Recruiting | •Breast Cancer | •Drug: Durvalumab,Tremeli | Interventional | Phase 2 | •Allocation: NonRandomized  •Intervention Model: Single Group Assignment  •Masking: Double (Participant, Care Provider)  •Primary Purpose: Treatment | Response rate based on RECIST1.1 | 33 | 20 Years and older (Adult, Older Adult) | Female | •Kyoto Breast  Cancer Research Network |
| Tremelimumab | NCT02489448 | Neoadjuvant MEDI4736 Concomitant With Weekly Nabpaclitaxel and Dose-dense AC for Stage I-III Triple Negative Breast Cancer | Recruiting | •Breast Neoplasms | •Drug: MEDI4736 | Interventional | •Phase 1  •Phase 2 | •Intervention Model: Single Group Assignment  •Masking: None (Open Label)  •Primary Purpose: Treatment | Pathologic Complete Response (pCR) | 61 | 18 Years and older (Adult, Older Adult) | All | •Yale University |
| Tremelimumab | NCT02997995 | Durvalumab and Endocrine Therapy in ER+/Her2- Breast Cancer After CD8+ Infiltration Effective Immune-Attractant Exposure | Recruiting | •Breast Cancer  •Estrogen Receptor  Positive Tumor  •Menopause  •Hormone Antagonist | •Drug: Immuneattractant  •Drug: Durvalumab  •Procedure: Biopsy | nterventional | Phase 2 | •Intervention Model: Single Group Assignment  •Masking: None (Open Label)  •Primary Purpose: Treatment | •pathological Complete Response  •Number of CD8+ T cell  •Clinical response  •Assessment of Ki67  •Toxicities  •Predictive value of Mutational load for efficacy of Durvalumab  •Predictive value of PDL1 expression for the efficacy of Durvalumab | 240 | 18 Years and older  (Adult, Older  Adult) | Female | •UNICANCER  •Breast  International  Group |
| Tremelimumab | NCT02639026 | Trial Of Hypofractionated Radiotherapy In Combination With MEDI4736 And Tremelimumab For Patients With Metastatic Melanoma And Lung, Breast And Pancreatic Cancers | Recruiting | •Metastatic  •Melanoma  •Non Small Cell Lung Cancer (NSCLC)  •Breast Cancer  •Pancreatic Cancer | •Radiation: Radiotherapy  •Drug: MEDI4736  •Drug: Tremelimumab | Interventional | Phase 1 | •Allocation: NonRandomized  •Intervention Model: Parallel Assignment  •Masking: None (Open Label)  •Primary Purpose: Treatment | Number of Adverse Events | 30 | 18 Years and older (Adult, Older Adult) | All | •Abramson  Cancer Center  of the University of Pennsylvania |
| Tremelimumab | NCT03518606 | Metronomic Oral Vinorelbine Plus Anti-PD-L1/Anti-CTLA4 ImmunothErapy in Patients With Advanced Solid Tumours | Recruiting | •Advanced Solid Tumours  •Breast Cancer  •Head and Neck Cancer  •Cervix Cancer  •Prostate Cancer | •Drug: Durvalumab + Tremelimumab + metronomic Vinorelbine | Interventional | •Phase 1  •Phase 2 | •Allocation: NonRandomized  •Intervention Model: Parallel Assignment  •Masking: None (Open Label)  •Primary Purpose: Treatment | •Maximum Tolerated Dose (MTD) and the phase II recommended dose (RP2D)  •CBR-24week | 150 | 18 Years and older (Adult, Older Adult) | All | •UNICANCER  •National Cancer Institute, France  •AstraZeneca  •Pierre Fabre  Laboratories |
| Tremelimumab | NCT02643303 | A Phase 1/2 Study of In Situ Vaccination With Tremelimumab and IV Durvalumab Plus PolyICLC in Subjects With Advanced, Measurable, Biopsyaccessible Cancers | Recruiting | •Head and Neck Squamous Cell Carcinoma  •Breast Cancer  •Sarcoma  •Merkel Cell Carcinoma  •Cutaneous T-Cell Lymphoma  •Melanoma  •Renal Cancer  •Bladder Cancer  •Prostate Cance | •Drug: Durvalumab  •Drug: Tremelimumab  •Drug: Poly ICLC | Interventional | •Phase 1  •Phase 2 | •Allocation: NonRandomized  •Intervention Model: Parallel Assignment  •Masking: None (Open Label)  •Primary Purpose: Treatment | •To determine the recommended combination doses of the dosing regimen, based on assessment of toxicity and tolerability.  •Clinical Efficacy will be determined by objective response rate (ORR), progression-free survival (PFS), and overall survival (OS) and will be assessed by irRECIST and RECIST1.1.  •Individual durvalumab concentrations  •Individual tremelimumab concentrations after IV and IT administration  •Number of subjects that develop changes in detectable antidrug antibodies to durvalumab  •Number of subjects that develop changes in detectable antidrug antibodies to tremelimumab  •Durvalumab and Tremelimumab Immunogenicity | 102 | 18 Years and older (Adult, Older Adult) | All | •Ludwig Institute for Cancer Research  •MedImmune LLC  •Cancer Research Institute, New York City |
| Tremelimumab | NCT02658214 | Durvalumab and Tremelimumab in Combination With First-Line Chemotherapy in Advanced Solid Tumors | Recruiting | •Small Cell Lung Carcinoma  •Carcinoma, Squamous Cell of Head and Neck  •Stomach Neoplasms  •Triple Negative Breast Neoplasms  •Ovarian Neoplasms  •Fallopian Tube Neoplasms  •Peritoneal Neoplasms  •Esophagogastric Junction Neoplasms •Carcinoma, Pancreatic Ductal  •Esophageal Squamous Cell Carcinoma | •Drug: paclitaxel + carboplatin  •Drug: carboplatin + etoposide  •Drug: gemcitabine + carboplatin  •Drug: nabpaclitaxel (paclitaxel-albumin) + carboplatin  •Drug: oxaliplatin + 5-fluorouracil (5FU) + leucovorin (calcium folinate/ folinic acid)  •Biological: durvalumab  •Biological: tremelimumab •Drug: nabpaclitaxel (paclitaxel-albumin) + gemcitabine  •Drug: cisplatin + 5- fluorouracil (5FU) | Interventional | Phase 1 | •Allocation: NonRandomized  •Intervention Model: Parallel Assignment  •Masking: None (Open Label)  •Primary Purpose: Treatment | •Laboratory findings (including: clinical chemistry, hematology, and urinalysis)  •Incidence of Adverse Events  •Tumor assessment based on RECIST 1.1 (for cohort 6 only) | 42 | 18 Years and older (Adult, Older Adult) | All | •AstraZeneca |
| Tremelimumab | NCT03674827 | A Study To Evaluate Escalating Doses of A Vaccine-Based Immunotherapy Regimen For NSCLC and TNBC | Not Yet Recruiting | •Non-Small Cell Lung Cancer  •Triple-negative Breast Cancer | •Biological: PF-06936308 | Interventional | Phase 1 | •Intervention Model: Single Group Assignment  •Masking: None (Open Label)  •Primary Purpose: Treatment | •Incidence and grade of treatment-emergent adverse events including DLTs  •To evaluate the immune response elicited by VBIR 2 to selected tumor antigens.  •To evaluate the antibody response to selected tumor-antigens  •Tremelimumab and anti PD-1 (PF-06801591) single dose PK parameter (Cmax)  •Tremelimumab and anti PD-1 (PF-06801591) single dose PK parameter (Tmax)  •Tremelimumab and anti PD-1 (PF-06801591) single dose PK parameter AUC  •Tremelimumab and anti PD-1 (PF-06801591) after multiple doses PK parameter (Ctrough)  •the anti drug antibody (ADA) response of tremelimumab and anti PD 1 (PF 06801591) after SC administration with the other components. | 97 | 18 Years and older (Adult, Older Adult) | All | •Pfizer |
