# Supplementary material for: Research Progresses in Immunological Checkpoint Inhibitors for Breast Cancer Immunotherapy
Source: Front Oncol. 2021 Sep 23;11:582664. doi: 10.3389/fonc.2021.582664 (PMC8495193; doi:10.3389/fonc.2021.582664)
Supplement: Supplementary Table 2 — Some ongoing clinical trials of anti-PD-1 immunotherapeutic interventions of malignancies including breast cancer. [file Table_2.doc]

| **Supplementary table 2, Some ongoing clinical trials of anti-PD-1 immunotherapeutic interventions of malignancies including breast cancer** | | | | | | | | | | | | | |
| --- | --- | --- | --- | --- | --- | --- | --- | --- | --- | --- | --- | --- | --- |
| **Drug** | **NCT Number** | **Title** | **Status** | **Conditions** | **Interventions** | **Characteristics** | | | | **Population** | | | **Sponsor/ Collaborators** |
|  |  |  |  |  |  | **Study Type** | **Phase** | **Study Design** | **Outcome Measures** | **Enrollment** | **Age** | **Sex** |  |
| Sintilimab | NCT03607539 | Efficacy and Safety Evaluation of Sintilimab in Patients With Advanced or Recurrent Nonsquamous NSCLC | Not Yet Recruiting | •Lung Neoplasms | •Drug: Sintilimab  •Drug: Pemetrexed  •Drug: Platinum  •Drug: Placebos | Interventional | Phase 3 | •Allocation: Randomized  •Intervention Model: Crossover Assignment  •Masking: Quadruple (Participant, Care Provider, Investigator, Outcomes Assessor)  •Primary Purpose: Treatment | •PFS (Progression Free Survival)  •OS (overall survival)  •ORR (overall response rate)  •DCR  •TTR  •DOR  •378 participants with treatment-related adverse events as assessed by CTCAE v4.03 | 378 | 18 Years and older (Adult, Older Adult) | All | •Innovent Biologics (Suzhou) Co. Ltd. |
| Sintilimab | NCT03545971 | A Study of IBI310 in Treatment of Patients With Advanced Solid Tumors | Recruiting | •Advanced Solid Tumors | •Drug: IBI310  •Drug: Sintilimab | Interventional | Phase 1 | •Allocation: Non-Randomized  •Intervention Model: Sequential Assignment  •Masking: None (Open Label)  •Primary Purpose: Treatment | •AEs •Pharmacokinetics#Cmax  •pharmacodynamics:lipid parameters  •ADA  •Pharmacokinetics#AUC | 74 | 18 Years to 70 Years (Adult, Older Adult) | All | •Innovent Biologics (Suzhou) Co. Ltd. |
| Sintilimab | NCT03700476 | Neoadjuvant and Concurrent PD-1 Blockade Combined With Definitive Chemoradiation in Nasopharyngeal Carcinoma | Not Yet Recruiting | •Nasopharyngeal Neoplasms | •Drug: Sintilimab  •Drug: Gemcitabine  •Drug: Cisplatin  •Radiation: intensitymodulated radiotherapy | Interventional | Phase 3 | •Allocation: Randomized  •Intervention Model: Parallel Assignment  •Masking: None (Open Label)  •Primary Purpose: Treatment | •Allocation: Randomized  •Intervention Model: Parallel Assignment  •Masking: None (Open Label)  •Primary Purpose: Treatment | 420 | 18 Years to 65 Years (Adult, Older Adult) | All | •Sun Yat-sen University  •Innovent Biologics (Suzhou) Co. Ltd. |
| Sintilimab | NCT03619824 | PD-1 Blockade Combined With Definitive Chemoradiation in Locoregionally-advanced Nasopharyngeal Carcinoma | Not Yet Recruiting | •Nasopharyngeal Neoplasms | •Drug: Sintilimab  •Drug: Gemcitabine  •Drug: Cisplatin  •Radiation: intensitymodulated radiotherapy | Interventional | Phase 2 | •Intervention Model: Single Group Assignment •Masking: None (Open Label)  •Primary Purpose: Treatment | •Immune-related adverse events (irAEs) and serious adverse events (irSAEs)  •All adverse events (AEs) and serious adverse events (SAEs)  •The proportion of patients who completed radiation within 8 weeks  •The proportion of patients who completed 6 cycles of sintilimab  •Failure-free survival  •Overall survival  •Distant failure-free survival  •Locoregional failure-free survival | 40 | 18 Years to 65 Years (Adult, Older Adult) | All | •Sun Yat-sen University  •Innovent Biologics (Suzhou) Co. Ltd |
| Sintilimab | NCT03629925 | Efficacy and Safety Evaluation of Sintilimab in Patients With Advanced or Recurrent Squamous NSCLC | Not Yet Recruiting | •Squamous NSCL | •Drug: Sintilimab  •Drug: Gemcitabine  •Drug: Placebo  •Drug: Platinum | Interventional | Phase 3 | •Allocation: Randomized  •Intervention Model: Crossover Assignment  •Masking: Quadruple (Participant, Care Provider, Investigator, Outcomes Assessor)  •Primary Purpose: Treatment | •PFS(progression free survival)  •OS (overall survival)  •ORR(objective response rate) | 348 | 18 Years to 75 Years (Adult, Older Adult) | All | •Innovent Biologics (Suzhou) Co. Ltd. |
| Spartalizumab | NCT03499899 | A Study of Efficacy and Safety of LAG525 in Combination With Spartalizumab, or With Spartalizumab and Carboplatin, or With Carboplatin, in Patients With Advanced Triple-negative Breast Cancer | Recruiting | •Triple-negative Breast Cancer | •Drug: LAG525  •Drug: spartalizumab  •Drug: carboplatin | Interventional | Phase 2 | •Allocation: Randomized  •Intervention Model: Parallel Assignment  •Masking: None (Open Label)  •Primary Purpose: Treatment | •Overall response rate (ORR) per RECIST v1.1 per investigators' assessment  •Duration of response (DOR)  •Overall Survival (OS)  •Pharmacokinetics (PK) parameter, Ctrough, of LAG525, spartalizumab and carboplatin  •Time to response (TTR)  •Progression free survival (PFS)  •Clinical Benefit Rate (CBR)  •PK parameter, Cmax of LAG525, spartalizumab and carboplatin  •PK parameter, AUC, of LAG525, spartalizumab and carboplatin  •Anti-drug antibodies (ADA) prevalence at baseline for LAG525 and spartalizumab  •Anti-drug antibodies (ADA) incidence on treatment for LAG525 and spartalizumab | 96 | 18 Years and older (Adult, Older Adult) | All | •Novartis Pharmaceuticals •Novartis |
| Spartalizumab | NCT03742349 | Study of Safety and Efficacy of Novel Immunotherapy Combinations in Patients With Triple Negative Breast Cancer (TNBC) | Not Yet Recruiting | •Triple Negative Breast Cancer (TNBC) | •Biological: spartalizumab  •Biological: LAG525  •Drug: NIR178  •Drug: capmatinib  •Biological: MCS110  •Biological: canakinumab | Interventional | Phase 1 | •Allocation: Non-Randomized  •Intervention Model: Parallel Assignment  •Masking: None (Open Label)  •Primary Purpose: Treatment | •Incidence of Adverse Events (AEs) and Serious Adverse Events (SAEs) as a measure of safety  •Severity of Adverse Events (AEs) and Serious Adverse Events (SAEs) as a measure of safety  •Incidence of dose limiting toxicities (DLTs) of treatment (Escalation only)  •Frequency of dose interuptions  •Frequency of dose reductions  •Dose intensities  •Best overall response (BOR)  •Progression free survival (PFS) per RECIST v1.1 and iRECIST  •Presence of antispartalizumab antibodies  •Presence of anti-LAG525 antibodies  •and 23 more | 220 | 18 Years and older (Adult, Older Adult) | All | •Novartis Pharmaceuticals •Novartis |
| Pembrolizumab | NCT03237572 | Focused Ultrasound and Pembrolizumab in Metastatic Breast Cancer | Recruiting | •Breast Cancer | •Drug: Pembrolizumab •Device: Highintensity focused ultrasound (HIFU) | Interventional | Phase 1 | •Allocation: Randomized  •Intervention Model: Parallel Assignment  •Masking: None (Open Label)  •Primary Purpose: Treatment | •Change in tumor infiltrating lymphocytes  •Adverse event profile of pembrolizumab and HIFU | 15 | 18 Years and older (Adult, Older Adult) | All | •Patrick Dillon, MD  •University of Virginia |
| Pembrolizumab | NCT03366844 | Breast Cancer Study of Preoperative Pembrolizumab + Radiation | Recruiting | •Breast Cancer | •Drug: Pembrolizumab •Radiation: RT Boost | Interventional | Early Phase 1 | •Intervention Model: Single Group Assignment •Masking: None (Open Label)  •Primary Purpose: Treatment | •Number of patients who do not necessitate a delay in standard of care treatment after receiving the investigational combination of preoperative Pembrolizumab and radiation  •Changes in Tumor Infiltrating Lymphocytes (TIL)  •Pembrolizumab-related adverse events  •Immune-related adverse events  •Invasive diseasefree survival after preoperative radiation and Pembrolizumab | 20 | 18 Years and older (Adult, Older Adult) | Female | •Stephen Shiao  •Cedars-Sinai Medical Center |
| Pembrolizumab | NCT03025035 | Pembrolizumab in Advanced BRCA-mutated Breast Cancer | Recruiting | •Breast Cancer | •Drug: Pembrolizumab | Interventional | Phase 2 | •Intervention Model: Single Group Assignment •Masking: None (Open Label)  •Primary Purpose: Treatment | •Overall response rate (ORR) per RECIST1.1  •Progression free survival (PFS), per RECIST 1.1  •Overall survival (OS)  •Clinical Benefit Rate (CBR = CR+PR+SD) per RECIST 1.1  •Duration of Response (DOR) for Complete Response (CR) and Partial Response (PR) per RECIST 1.1 | 20 | 18 Years and older (Adult, Older Adult) | All | •Monica Mita  •Merck Sharp & Dohme Corp. •Cedars-Sinai Medical Center |
| Pembrolizumab | NCT02999477 | A Study Of Changes In PD-L1 Expression During Preoperative Treatment With Nab-Paclitaxel And Pembrolizumab In Hormone Receptor-Positive Breast Cancer | Recruiting | • Breast Cancer | •Drug: Pembrolizumab •Drug: NabPaclitaxel  •Procedure: Biopsy | Interventional | Phase 1 | •Allocation: Randomized  •Intervention Model: Parallel Assignment  •Masking: None (Open Label)  •Primary Purpose: Treatment | •Change in PDL1 Expression By Immunohistochemistry From Baseline Biopsy to Biopsy After 2-Week Treatment (biopsy 2)  •The Absolute Change in PD-L1 Expression By Immunohistochemistry From Baseline Biopsy to Biopsy After Treatment with Nab-Paclitaxel or Pembrolizumab Monotherapy (biopsy 3)  •The Absolute Change in Expression of Core Immune Biomarkers (stromal TILs; PD-1; PDL2; CD8) from Baseline Biopsy to Biopsy After 2- Week Treatment (biopsy 2)  •The Absolute Change in Expression of Core Immune Biomarkers (stromal TILs; PD-1; PD-L2; CD8) from Baseline Biopsy to Biopsy After Treatment with Nab-Paclitaxel or Pembrolizumab Monotherapy (biopsy 3)  •Maximum Grade Of All Treatment-Related Adverse Events  •Pathologic Complete Response Rate  •Overall Response Rate  •Disease-Free Survival | 50 | 18 Years and older (Adult, Older Adult) | All | •Dana-Farber Cancer Institute  •Merck Sharp & Dohme Corp. |
| Pembrolizumab | NCT03393845 | Study of Pembrolizumab Plus Fulvestrant in Hormone Receptor Positive, HER-2 Negative Advanced/Metastatic Breast Cancer Patients | Recruiting | • Breast Cancer | •Drug: Pembrolizumab •Drug: Fulvestrant | Interventional | Phase 2 | •Intervention Model: Single Group Assignment •Masking: None (Open Label)  •Primary Purpose: Treatmen | •Overall Response Rate  •Safety profile of pembrolizumab plus fulvestrant | 47 | 18 Years and older (Adult, Older Adult) | All | •Nancy Chan, MD •Merck Sharp & Dohme Corp.  •Big Ten Cancer Research Consortium |
| Pembrolizumab | NCT03051659 | A Randomized Phase II Study Of Eribulin Mesylate With or Without Pembrolizumab For Metastatic Hormone Receptor Positive Breast Cancer | Recruiting | •Breast Cancer | •Drug: Eribulin Mesylate  •Drug: Pembrolizumab | Interventional | Phase 2 | •Allocation: Randomized  •Intervention Model: Parallel Assignment  •Masking: None (Open Label)  •Primary Purpose: Treatment | •Progression Free Survival  •Objective Response Rate  •Overall Survival Rate  •Immune Response Rate  •Duration of Response  •Clinical Benefit Rate  •Incidence Rate of each Toxicity (safety and tolerability). | 88 | 18 Years and older (Adult, Older Adult) | All | •Dana-Farber Cancer Institute  •Merck Sharp & Dohme Corp. |
| Pembrolizumab | NCT03362060 | PVX-410 Vaccine Plus Pembrolizumab in HLA-A2+ Metastatic Triple Negative Breast Cancer | Recruiting | •Triple Negative Breast Cancer  •Metastatic Breast Cancer | •Drug: Pembrolizumab •Biological: PVX-410 | Interventional | Phase 1 | •Intervention Model: Single Group Assignment •Masking: None (Open Label)  •Primary Purpose: Treatment | •Immune Response following treatment with PVX-410 in combination with pembrolizumab •Late Immune response after treatment with PVX-410 and pembrolizumab  •Incidence of treatment emergent adverse events (safety and tolerability) of PVX-410 in combination with pembrolizumab •Progression Free Survival  •Overall Survival  •Response rate  •Disease Control Rate  •Clinical Benefit Rate  •Duration of response | 20 | 18 Years and older (Adult, Older Adult) | All | •Massachusetts General Hospital •Merck Sharp & Dohme Corp. |
| Pembrolizumab | NCT03225547 | Study of Pembrolizumab and Mifepristone in Patients With Advanced HER2-negative Breast Cancer | Recruiting | •Advanced HER2- negative Breast Cancer | •Drug: Pembrolizumab •Drug: Mifepristone | Interventional | Phase 2 | •Allocation: Non-Randomized  •Intervention Model: Parallel Assignment  •Masking: None (Open Label)  •Primary Purpose: Treatment | •Rate of overall response based on RECIST 1.1  •Number of patients with adverse events  •Rate of overall response based on irRECIST | 74 | 18 Years and older (Adult, Older Adult) | All | •University of Chicago |
| Pembrolizumab | NCT03139851 | Evaluation of Pembrolizumab in Lymphopenic Metastatic Breast Cancer Patients Treated With Metronomic Cyclophosphamide | Recruiting | •Metastatic Breast Cancer | •Drug: Cyclophosphamide 50mg  •Drug: Pembrolizumab 100 MG in 4 ML Injectio | Interventional | Phase 2 | •Intervention Model: Single Group Assignment •Masking: None (Open Label)  •Primary Purpose: Treatment | •Severe Toxicities (ST) in Run-In Phase Part  •24-week Clinical Benefit Rate (CBR24w) in Phase II part  •ECI (Events of Clinical Interest)  •Overall response Rate (ORR) at 24 weeks  •Duration of response (DoR)  •Progression-Free Survival (PFS) •Overall Survival (OS)  •Adverse events reporting | 36 | 18 Years and older (Adult, Older Adult) | Female | •Centre Leon Berard  •Merck Sharp & Dohme Corp. |
| Pembrolizumab | NCT02513472 | Study to Evaluate the Efficacy and Safety of Eribulin Mesylate in Combination With Pembrolizumab in Participants With Metastatic Triple-Negative Breast Cancer (mTNBC) | Recruiting | • Breast Cancer | •Drug: Eribulin Mesylate  •Drug: Pembrolizumab | Interventional | •Phase 1  •Phase 2 | •Intervention Model: Single Group Assignment •Masking: None (Open Label)  •Primary Purpose: Treatment | •Objective Response Rate (ORR)  •Progression-Free Survival (PFS) •Overall Survival (OS)  •Duration of Response (DOR)  •Clinical Benefit Rate (CBR)  •ORR in the Programmed Death Receptor-Ligand 1 (PD-L1) positive set  •PFS in the PD-L1 positive set  •OS in the PD-L1 positive set  •DOR in the PD-L1 positive set  •CBR in the PD-L1 positive set | 147 | 18 Years and older (Adult, Older Adult) | All | •Eisai Inc.  •Merck Sharp & Dohme Corp. |
| Pembrolizumab | NCT03591276 | Phase 1b Study of Pegylated Liposomal Doxorubicin and Pembrolizumab in Endocrineresistant Breast Cancer | Not yet Recruiting | •Metastatic Breast Cancer | •Drug: Chemotherapy Drugs, Cancer | Interventional | •Phase 1  •Phase 2 | •Intervention Model: Single Group Assignment •Masking: None (Open Label)  •Primary Purpose: Treatment | •To establish a safe dose of PLD when delivered in combination with pembrolizumab  •To evaluate the Tumor Response Rate according to Response Evaluation Criteria in Solid Tumors (RECIST) after 9 weeks (3 cycles) of treatment in patients with measurable disease.  •To evaluate the safety and DLT of the PLD and pembrolizumab combination, as indicated by the number of study participants with treatmentrelated Adverse Events, and the class of Adverse Events as assessed by CTCAE v4.0.  •To characterize the PK of PLD when delivered in combination with pembrolizumab, by measuring and comparing the Area under the curve (AUC) for doxorubicin (liposomal) obtained during Cycles 1 and 3. | 15 | 18 Years and older (Adult, Older Adult) | Female | •Shaare Zedek Medical Center |
| Pembrolizumab | NCT03492918 | Pembrolizumab in Hormone Receptor-positive, hyperMUTATted Metastatic Breast Cancer Identified by Whole exOme sequeNcing ('MUTATION2') | Not yet Recruiting | •Metastatic Breast Cancer | •Drug: Pembrolizumab | Interventional | Phase 2 | •Intervention Model: Single Group Assignment •Masking: None (Open Label)  •Primary Purpose: Treatment | •Change of objective response rate(ORR) by RECIST 1.1  •Clinical benefit rate(CBR) by RECIST 1.1 | 30 | 19 Years and older (Adult, Older Adult) | Female | •Yonsei University |
| Pembrolizumab | NCT02977468 | Effects of MK-3475 (Pembrolizumab) on the Breast Tumor Microenvironment in Triple Negative Breast Cancer | Recruiting | •Metastatic Triple Negative Breast Cancer | •Drug: Merck 3475 Pembrolizumab  •Radiation: Intraoperative radiation therapy (IORT) | Interventional | Phase 1 | •Intervention Model: Single Group Assignment •Masking: None (Open Label)  •Primary Purpose: Treatment | Number of subjects with signifcant mean percent change in TILs | 15 | 21 Years to 80 Years (Adult, Older Adult) | Female | •Eileen Connolly •Merck Sharp & Dohme Corp.  •Columbia University |
| Pembrolizumab | NCT03632941 | A Study to Evaluate Concurrent VRP-HER2 Vaccination and Pembrolizumab for Patients With Breast Cancer | Not yet Recruiting | •Breast Cancer  •HER2+ Breast Cancer | •Biological: VRPHER2 •Biological: Pembrolizumab | Interventional | Phase 2 | •Allocation: Randomized  •Intervention Model: Parallel Assignment  •Masking: None (Open Label)  •Primary Purpose: Treatment | •Number of Tumor infiltrating Lymphocytes and HER2 specific antibodies  •Rate and severity of Adverse Events | 39 | 18 Years and older (Adult, Older Adult) | All | •Herbert Lyerly  •Merck Sharp & Dohme Corp.  •Duke University |
| Pembrolizumab | NCT02990845 | Pembrolizumab and Exemestane/ Leuprolide in Premenopausal HR+/ HER2- Locally Advanced or Metastatic Breast Cancer | Not yet Recruiting | •Premenopausal Breast Cancer | •Drug: Pembrolizumab/ Exemestane/ Leuprolide | Interventional | •Phase 1  •Phase 2 | •Intervention Model: Single Group Assignment •Masking: None (Open Label)  •Primary Purpose: Treatment | •The PFS rate at 8 months  •Number of participants with treatment-related adverse events as assessed by CTCAE v4.0  •The PFS based on RECIST 1.1  •The overall response rate (ORR) based on RECIST 1.1  •The clinical benefit rate (CBR) based on RECIST 1.1  •The duration of overall response (DOR) based on RECIST 1.1 | 25 | 20 Years and older (Adult, Older Adult) | All | •National Taiwan University Hospital  •Merck Sharp & Dohme Corp. |
| Pembrolizumab | NCT02411656 | MK-3475 for Metastatic Inflammatory Breast Cancer (MIBC) | Recruiting | • Breast Cancer | •Drug: MK-3475  •Behavioral: Follow Up/Phone Call | Interventional | Phase 2 | •Intervention Model: Single Group Assignment •Masking: None (Open Label)  •Primary Purpose: Treatment | Disease Control Rate | 35 | 18 Years and older (Adult, Older Adult) | All | •M.D. Anderson Cancer Center  •Merck Sharp & Dohme Corp. |
| Pembrolizumab | NCT03720431 | TTAC-0001 and Pembrolizumab Phase Ib Combination Trial in Metastatic Triple-negative Breast Cancer | Not yet Recruiting | •Triple Negative Breast Cancer | •Drug: TTAC-0001 and pembrolizumab combination | Interventional | Phase 1 | •Intervention Model: Sequential Assignment  •Masking: None (Open Label)  •Primary Purpose: Treatment | •Dose limiting toxicities  •Adverse events  •Immunogenicity  •Overall response rate  •Disease control rate  •Progression free survival  •Overall survival | 20 | 18 Years and older (Adult, Older Adult) | All | •PharmAbcine |
| Pembrolizumab | NCT03272334 | Her2-BATS and Pembrolizumab in Metastatic Breast Cancer | Recruiting | •Metastatic Breast Cancer | •Drug: HER2 BATs with Pembrolizumab | Interventional | •Phase 1  •Phase 2 | •Allocation: NonRandomized  •Intervention Model: Sequential Assignment  •Masking: None (Open Label)  •Primary Purpose: Treatment | •Dose Limiting Toxicities on each schedule/arm  •Dose Limiting Toxicities on the selected arm in the expansion cohort  •Immune response to treatment in blood  •Disease control rate  •Objective response rate  •Duration of response  •Survival | 33 | 18 Years and older (Adult, Older Adult) | Female | •University of Virginia  •Merck Sharp & Dohme Corp. |
| Pembrolizumab | NCT02734290 | Standard of Care Chemotherapy Plus Pembrolizumab for Breast Cancer | Recruiting | • Triple Negative Breast Cancer | •Drug: Pembrolizumab •Drug: Paclitaxel  •Drug: Capecitabine | Interventional | •Phase 1  •Phase 2 | •Allocation: NonRandomized  •Intervention Model: Parallel Assignment  •Masking: None (Open Label)  •Primary Purpose: Treatment | •Treatment-Associated Adverse Events  •Number of patients who complete chemotherapy without a dose delay of more than 21 days.  •Overall response rate | 88 | 18 Years and older (Adult, Older Adult) | All | •Providence Health & Services •Merck Sharp & Dohme Corp |
| Pembrolizumab | NCT03732391 | Phase 2 Study of Pembrolizumab+Carboplatin in Breast Related Cancer Antigens-related Metastatic Breast Cancer (PEMBRACA) | Not yet Recruiting | •Metastatic Breast Cancer | •Drug: Pembrolizumab 25 MG(milligram)/ML | Interventional | Phase 2 | •Intervention Model: Single Group Assignment •Masking: None (Open Label)  •Primary Purpose: Treatment | •The ORR  •TTP (time to tumor progression) | 53 | 18 Years and older (Adult, Older Adult) | All | •CORTESI LAURA •Azienda Ospedaliero-Universitaria di Modena |
| Pembrolizumab | NCT03121352 | Carboplatin, Nab-Paclitaxel and Pembrolizumab for Metastatic Triple-Negative Breast Cancer | Recruiting | •Metastatic Triple Negative Breast Cancer | •Drug: Carboplatin  •Drug: Nabpaclitaxel  •Drug: Pembrolizumab | Interventional | Phase 2 | •Intervention Model: Single Group Assignment •Masking: None (Open Label)  •Primary Purpose: Treatment | •Determine overall response rate (ORR) in patients treated with CNP  •Determine progressionfree survival (PFS) in patients treated with CNP  •Determine disease control rate (DCR) in patients treated with CNP •Determine duration of response in patients treated with CNP | 30 | 18 Years and older (Adult, Older Adult) | All | •Case Comprehensive Cancer Center |
| Pembrolizumab | NCT0372505 | Study of Pembrolizumab (MK-3475) Versus Placebo in Combination With Neoadjuvant Chemotherapy & Adjuvant Endocrine Therapy in the Treatment of Early-Stage Estrogen Receptor-Positive, Human Epidermal Growth Factor Receptor 2-Negative (ER+/HER2-) Breast Cancer (MK-3475-756/KEYNOTE-756) | Not yet Recruiting | • Breast Cancer | •Biological: Pembrolizumab (K)  •Drug: Placebo (P)  •Drug: Paclitaxel (X)  •Drug: Doxorubicin hydrochloride (A)  •Drug: Epirubicin (E)  •Drug: Cyclophosphamide (C) •Drug: Endocrine therapy  •Radiation: Radiation therapy  •Procedure: Surgery | Interventional | Phase 3 | •Allocation: Randomized  •Intervention Model: Parallel Assignment  •Masking: Triple (Participant, Investigator, Outcomes Assessor)  •Primary Purpose: Treatment | •Pathological Complete Response (pCR) Rate Using the Definition of ypT0/Tis ypN0  •Event-Free Survival (EFS)  •Overall Survival (OS)  •pCR Rate Using the Definition of ypT0ypN0  •pCR Rate Using the Definition of ypT0/Tis  •pCR Rate Using the Definitions of ypT0/Tis ypN0, ypT0/Tis, and ypT0 ypN0 in Participants With a Combined Positive Score [CPS] #1 •EFS in Participants With a CPS #1 •OS in Participants With a CPS #1 •Number of Participants Experiencing an Adverse Event (AE)  •Number of Participants Experiencing a Serious Adverse Event (SAE)  •and 4 more | 1140 | 18 Years and older (Adult, Older Adult) | All | •Merck Sharp & Dohme Corp. |
| Pembrolizumab | NCT03032107 | A Study Of Pembrolizumab In Combination With TrastuzumabDM1 | Recruiting | • Breast Cancer | •Drug: T-DM1  •Drug: Pembrolizumab | Interventional | Phase 1 | •Intervention Model: Single Group Assignment •Masking: None (Open Label)  •Primary Purpose: Treatment | •Incidence of TreatmentEmergent Adverse Events [Safety and Tolerability]  •Objective Response Rate  •Progression Free Survival  •Duration Of Response  •Disease Control Rate  •Overall Survival Rate | 27 | 18 Years and older (Adult, Older Adult) | All | •Dana-Farber Cancer Institute  •Merck Sharp & Dohme Corp |
| Pembrolizumab | NCT03515798 | Study of Immunotherapy in Combination With Chemotherapy in HER2- negative Inflammatory Breast Cancer | Not yet Recruiting | •Inflammatory Breast Cancer | •Drug: Pembrolizumab Injection  •Drug: neoadjuvant (F)EC-paclitaxel chemotherapy | Interventional | Phase 2 | •Allocation: Randomized  •Intervention Model: Parallel Assignment  •Masking: None (Open Label)  •Primary Purpose: Treatment | •Central evaluation of pathological complete response rate  •Dose Limiting Toxicity (DLT) rates  •occurrence of serious adverse events and adverse events starting grade 2 or grade 1 (run-in period) •Local evaluation of pathological complete response rate  •Invasive disease-free survival (IDFS)  •Event free survival (EFS)  •Overall survival (OS) | 81 | 18 Years and older (Adult, Older Adult) | All | •Institut PaoliCalmettes  •MSD France |
| Pembrolizumab | NCT03145961 | A Trial Using ctDNA Blood Tests to Detect Cancer Cells After Standard Treatment to Trigger Additional Treatment in Early Stage Triple Negative Breast Cancer Patients | Recruiting | • Triple Negative Breast Cancer | •Drug: Pembrolizumab | Interventional | Phase 2 | •Allocation: Randomized  •Intervention Model: Parallel Assignment  •Masking: Double (Participant, Care Provider)  •Primary Purpose: Treatment | •Positive ctDNA detection by 12 months  •Positive ctDNA detection by 24 months  •Absence of detectable ctDNA or disease recurrence 12 months after commencing pembrolizumab  •Time to ctDNA detection  •Detection of overt metastatic disease at time of first ctDNA detection in patients allocated to pembrolizumab  •Lead time between ctDNA detection and disease recurrence in the pembrolizumab treatment and observation groups  •Absence of detectable ctDNA or disease recurrence after 12 months in the observation group  •Safety and tolerability of pembrolizumab assessed using NCI CTCAE v4.0, and the proportion of patients reporting dose reductions or delays.  •Commencement of treatment in patients randomised to receive pembrolizumab | 200 | 16 Years and older (Child,Adult, Older Adult) | All | •Institute of Cancer Research, United Kingdom  •National Institute for Health Research Biomedical Research Centre at the Royal Marsden / Institute of Cancer Research UK  •Merck Sharp & Dohme Corp. |
| Pembrolizumab | NCT03051672 | Phase II PEMBROLIZUMAB + PALLIATIVE RADIOTHERAPY IN BC | Recruiting | •Metastatic Breast Cancer | •Drug: Pembrolizumab  •Radiation: Palliative radiotherapy | Interventional | Phase 2 | •Intervention Model: Single Group Assignment  •Masking: None (Open Label)  •Primary Purpose: Treatment | •Overall Response Rate  •Immune Response Rate  •Clinical Benefit Response Rate  •Progression Free Survival  •Absolute Risk Reduction | 27 | 18 Years and older (Adult, Older Adult) | All | •Dana-Farber Cancer Institute  •Merck Sharp & Dohme Corp. |
| Pembrolizumab | NCT03310957 | Safety and Efficacy of SGNLIV1A Plus Pembrolizumab for Patients With Locally-Advanced or Metastatic Triple-Negative Breast Cancer | Recruiting | •Breast Neoplasms | •Drug: SGN-LIV1A  •Drug: Pembrolizumab | Interventional | •Phase 1  •Phase 2 | •Intervention Model: Single Group Assignment  •Masking: None (Open Label)  •Primary Purpose: Treatment | •Confirmed objective response rate  •Incidence of adverse events  •Incidence of laboratory abnormalities  •Incidence of dose-limiting toxicities  •Duration of response  •Disease control rate  •Progression-free survival  •Overall survival | 72 | 18 Years and older (Adult, Older Adult) | All | •Seattle Genetics, Inc.  •Merck Sharp & Dohme Corp. |
| Pembrolizumab | NCT01676753 | Phase II PEMBROLIZUMAB + PALLIATIVE RADIOTHERAPY IN BC | Recruiting | •Advanced or Metastatic Breast Cancer  •Triple Negative Breast Cancer | •Drug: Dinaciclib  •Drug: Pembrolizumab | Interventional | Phase 1 | •Intervention Model: Single Group Assignment  •Masking: None (Open Label)  •Primary Purpose: Treatment | •Maximum tolerated dose (MTD)  •Define dose-limiting toxicities (DLTs)  •Anti-tumor activity in patients with advanced triple negative breast cancer | 32 | 18 Years to 85 Years (Adult, Older Adult) | All | •Jo Chien  •Merck Sharp & Dohme Corp.  •University of California, San Francisco |
| Pembrolizumab | NCT03197389 | Effect of Pembrolizumab (Keytruda®) on Biomarkers in Early ER/PR Negative Breast Cancer | Recruiting | •Breast Cancer  •Triple Negative Breast Cancer  •Hormone Receptor Negative Neoplasm | •Drug: Pembrolizumab | Interventional | Early Phase 1 | •Allocation: NonRandomized  •Intervention Model: Single Group Assignment  •Masking: None (Open Label)  •Primary Purpose: Basic Science | •PD-1 expression  •Increase in the amount of TILs  •PD-L1 expression | 34 | 18 Years to 100 Years (Adult, Older Adult) | All | •Universitaire Ziekenhuizen Leuven |
| Pembrolizumab | NCT03025880 | Trial to Evaluate Efficacy and Safety of Pembrolizumab and Gemcitabine in HER2-negative ABC | Recruiting | •Advanced Breast Cancer | •Drug: Pembrolizumab  •Drug: Gemcitabine | Interventional | Phase 2 | •Intervention Model: Single Group Assignment  •Masking: None (Open Label)  •Primary Purpose: Treatment | •Incidence rate of Dose Limiting Toxicity (DLT) within the first cycle  •Recommended Phase II Dose (RP2D) of gemcitabine in combination with pembrolizumab  •Objective Response Rate (ORR)  •Progression-Free Survival (PFS)  •Clinical Benefit Rate (CBR)  •Response Duration (RD)  •Overall Survival (OS).  •The Number of Participants Who Experienced Adverse Events (AE) | 65 | 18 Years and older (Adult, Older Adult) | Female | •Spanish Breast Cancer Research Group  •Merck Sharp & Dohme Corp |
| Pembrolizumab | NCT03639948 | Neoadjuvant Phase II Study of Pembrolizumab And Carboplatin Plus Docetaxel in Triple Negative Breast Cancer | Recruiting | •Triple-Negative Breast Cancer | •Drug: Carboplatin  •Drug: Docetaxel  •Drug: Pembrolizumab  •Drug: Pegfilgrastim | Interventional | Phase 2 | •Intervention Model: Single Group Assignment  •Masking: None (Open Label)  •Primary Purpose: Treatment | •Pathological complete response (pCR) rate  •Minimal residual disease (MRD) rate  •Recurrence-free survival (RFS) | 100 | 18 Years to 70 Years (Adult, Older Adult) | Female | •University of Kansas Medical Center  •Merck Sharp & Dohme Corp. |
| Pembrolizumab | NCT02981303 | Study of Imprime PGG and Pembrolizumab in Advanced Melanoma and Triple Negative Breast Cancer | Recruiting | •Advanced Melanoma  •Triple-Negative Breast Cancer | •Biological: Imprime PGG  •Drug: Pembrolizumab | Interventional | Phase 2 | •Allocation: NonRandomized  •Intervention Model: Parallel Assignment  •Masking: None (Open Label)  •Primary Purpose: Treatment | •Overall Response Rate (ORR) to Imprime PGG + pembrolizumab using RECIST v1.1 criteria  •Time to response (TTR) using RECIST v1.1 criteria  •Complete response rate (CRR) using RECIST v1.1 criteria  •Duration of overall response (DoR) using RECIST v1.1 criteria  •Progression-Free Survival (PFS) and PFS rate at 6 months and 1 year using RECIST v1.1 criteria  •Overall survival (OS) and OS rate at 1 year using RECIST v1.1 criteria  •Pharmacokinetic (PK) data of Imprime PGG in combination with Pembrolizumab | 95 | 18 Years and older (Adult, Older Adult) | All | •Biothera  •Merck Sharp & Dohme Corp. |
| Pembrolizumab | NCT03222856 | Ph II Study of Pembrolizumab & Eribulin in Patients With HR+/ HER2- MBC Previously Treated With Anthracyclines & Taxanes | Recruiting | • Breast Cancer | •Drug: Pembrolizumab  •Drug: Eribulin | Interventional | Phase 2 | •Intervention Model: Single Group Assignment  •Masking: None (Open Label)  •Primary Purpose: Treatment | •Efficacy of pembrolizumab in combination with eribulin.  •The CBR in subjects with programmed death ligand-1 (PD-L1) positive tumors.  •The progression-free survival (PFS)  •The PFS in subjects with PD-L1 positive tumors.  •The overall survival OS  •The OS in subjects with PD-L1 positive tumors.  •The overall response rate (ORR)  •The ORR in subjects with PD-L1 positive tumors.  •The duration of response (DoR)  •The DoR in subjects with PD-L1 positive tumors.  •Safety and tolerability of pembrolizumab in combination with eribulin | 44 | 18 Years and older (Adult, Older Adult) | Female | •MedSIR |
| Pembrolizumab | NCT03449238 | Pembrolizumab And Stereotactic Radiosurgery (Srs) Of Selected Brain Metastases In Breast Cancer Patients | Not yet Recruiting | •Metastatic Breast Cancer  •Brain Metastases | •Drug: Pembrolizumab | Interventional | •Phase 1  •Phase 2 | •Intervention Model: Single Group Assignment  •Masking: None (Open Label)  •Primary Purpose: Treatment | •Tumor response for nonirradiated brain lesions at 8 weeks according to RECIST1.1  •Correlation of abscopal responses with the radiation dose received  •Overall survival - assessed from the start of study drug until death in nonirradiation metastases in the rest of the body by routine imaging. | 41 | 19 Years to 90 Years (Adult, Older Adult) | Female | •Weill Medical College of Cornell University |
| Pembrolizumab | NCT03018080 | Pilot Study of Paclitaxel Plus Pembrolizumab in Metastatic HER2-Negative Breast Cancer | Recruiting | •Breast - Female  •Male Breast Cancer | •Drug: Pembrolizumab  •Drug: Paclitaxel | Interventional | Phase 2 | •Allocation: NonRandomized  •Intervention Model: Parallel Assignment  •Masking: None (Open Label)  •Primary Purpo | •Grade 3 or 4 treatmentrelated adverse event  •Objective response rate  •Progression-free survival  •Overall survival  •Disease control rate  •Duration of response | 40 | 18 Years and older (Adult, Older Adult) | All | •Antoinette Tan  •Merck Sharp & Dohme Corp.  •Carolinas Healthcare System |
| Pembrolizumab | NCT033280262 | Combination Study of SVBR-1-GM in Combination With Pembrolizumab | Recruiting | •Breast Cancer Female  •Breast Neoplasm Female | •Biological: SVBR-1-GM  •Biological: Pembrolizumab  •Drug: Low dose cyclophosphamide  •Biological: Interferon Inoculation | Interventional | •Phase 1  •Phase 2 | •Intervention Model: Single Group Assignment  •Masking: None (Open Label)  •Primary Purpose: Treatment | •Evaluate the Safety of SV-BR-1-GM (Adverse Events) when administered in combination with ipilimumab (for patients with PD-L1/2- tumors) or pembrolizumab (for patients with PD-L1/2+ tumors) [Safety]  •Evaluate the Safety of SV-BR-1-GM (Laboratory Parameters) when administered in combination with ipilimumab (for patients with PD-L1/2- tumors) or pembrolizumab (for patients with PD-L1/2+ tumors) [Safety]  •Evaluate the tumor response to SV-BR-1-GM (ORR) when administered in combination with ipilimumab (for patients with PD-L1/2- tumors) or pembrolizumab (for patients with PD L1/2+ tumors)  •Evaluate the tumor response to SV-BR-1- GM (Non-progression) when administered in combination with ipilimumab (for patients with PD-L1/2- tumors) or pembrolizumab (for patients with PD L1/2+ tumors)  •Evaluate the tumor response to SV-BR-1-GM (Durability of response) when administered in combination with ipilimumab (for patients with PD-L1/2- tumors) or pembrolizumab (for patients with PD L1/2+ | 40 | 18 Years and older (Adult, Older Adult) | Female | •BriaCell Therapeutics Corporation  •Cancer Insight, LLC |
| Pembrolizumab | NCT03095352 | A Randomized Phase II Study of Pembrolizumab, an Anti-PD (Programmed Cell Death)-1 Antibody, in Combination With Carboplatin Compared to Carboplatin Alone in Breast Cancer Patients With Chest Wall Disease | Recruiting | •Breast Cancer  •Chest Wall Disease | •Biological: Pembrolizumab  •Drug: Carboplatin  •Biological: Trastuzumab | Interventional | Phase 2 | •Allocation: Randomized  •Intervention Model: Crossover Assignment  •Masking: None (Open Label)  •Primary Purpose: Treatment | •Disease control rate  •Objective response rate (ORR) defined as CR/PR assessed by RECIST  •Progression free survival  •ORR defined as CR/PR assessed by immunerelated (ir)RECIST | 84 | 18 Years and older (Adult, Older Adult) | All | •University of California, San Francisco  •Mayo Clinic  •Johns Hopkins University  •Translational Breast Cancer Research Consortium  •Massachusetts General Hospita |
| Pembrolizumab | NCT03184558 | Bemcentinib (BGB324) in Combination With Pembrolizumab in Patients With TNBC | Recruiting | •Triple Negative Breast Cancer  •Inflammatory Breast Cancer Stage IV | •Drug: Bemcentinib; pembrolizumab | Interventional | Phase 2 | •Intervention Model: Single Group Assignment  •Masking: None (Open Label)  •Primary Purpose: Treatment | •Objective Response Rate  •Disease Control Rate  •Duration of Response  •Time to Progression  •Overall Survival  •Number of patients with Adverse Events (as assessed by CTCAE v4.03) | 56 | 18 Years and older (Adult, Older Adult) | All | •BerGenBio ASA  •Merck Sharp & Dohme Corp. |
| Pembrolizumab | NCT03644589 | Effect of Pembrolizumab and Cisplatin on Metastatic, Locally Recurrent or Inoperable TripleNegative Breast Cancer | Not Yet Recruiting | •Estrogen Receptor Negative  •HER2/Neu Negative  •Metastatic Breast Cancer  •Progesterone Receptor Negative  •Recurrent Breast Carcinoma  •Triple Negative Breast Cancer | •Biological: Pembrolizumab  •Drug: Cisplatin | Interventional | Phase 2 | •Intervention Model: Single Group Assignment  •Masking: None (Open Label)  •Primary Purpose: Treatment | •To evaluate the efficacy of combination pembrolizumab and cisplatin in participants with advanced TNBC  •Safety of pembrolizumab and cisplatin in participants with advanced TNBC  •Disease Control Rate  •Disease control rate (DCR)  •Duration of response (DoR)  •Progression-free survival  •Overall survival (OS) in participants with advanced TNBC treated with pembrolizumab and cisplatin | 60 | 18 Years and older (Adult, Older Adult) | All | •University of Washington  •Merck Sharp & Dohme Corp. |
| Pembrolizumab | NCT02971748 | A Study of Anti-PD-1 (Pembrolizumab) + Hormonal Therapy in HR-positive Localized IBC Patients With Non-pCR to Neoadjuvant Chemotherapy | Recruiting | •Inflammatory Breast Cancer  •Malignant Neoplasm of Breast | •Drug: Pembrolizumab | Interventional | Phase 2 | •Intervention Model: Single Group Assignment  •Masking: None (Open Label)  •Primary Purpose: Treatment | Disease-free survival (DFS) | 37 | 18 Years and older (Adult, Older Adult) | All | •M.D. Anderson Cancer Center  •Merck Sharp & Dohme Corp. |
| Pembrolizumab | NCT03567720 | Intratumoral Tavo and Pembro in Patients With Inoperable Locally Advanced or Metastatic TNBC | Recruiting | • Triple Negative Breast Cancer | •Biological: tavokinogene telseplasmid  •Biological: Pembrolizumab  •Device: Immunopulse | Interventional | Phase 2 | •Intervention Model: Single Group Assignment  •Masking: None (Open Label)  •Primary Purpose: Treatment | •Objective Response Rate (ORR)  •Duration of Response (DOR)  •Progression Free Survival (PFS)  •Overall Survival | 25 | 18 Years and older (Adult, Older Adult) | All | •OncoSec Medical Incorporated |
| Pembrolizumab | NCT02755272 | A Study of Pembrolizumab With Carboplatin and Gemcitabine in Patients With Metastatic Triple Negative Breast Cancer | Recruiting | •Carcinoma Breast Stage IV | •Drug: Pembrolizumab  •Drug: Carboplatin  •Drug: Gemcitabine | Interventional | Phase 2 | •Allocation: Randomized  •Intervention Model: Parallel Assignment  •Masking: None (Open Label)  •Primary Purpose: Treatment | •Objective Response Rate  •Incidence of TreatmentRelated Adverse Events  •Clinical Benefit Rate  •Progression Free Survival  •Overall Survival | 87 | 18 Years and older (Adult, Older Adult) | Female | •Fox Chase Cancer Center |
| Pembrolizumab | NCT03289819 | Neoadjuvant Pembrolizumab(Pbr)/NabPaclitaxel Followed by Pbr/ Epirubicin/Cyclophosphamide in TNBC | Recruiting | •Malignant Neoplasm of Breast | •Drug: Pembrolizumab  •Drug: nabpaclitaxel  •Drug: Epirubicin  •Drug: Cyclophosphamide | Interventional | Phase 2 | •Intervention Model: Single Group Assignment  •Masking: None (Open Label)  •Primary Purpose: Treatment | •Pathological Complete Response (pCR) rate  •Number of participants with treatment-related adverse events as assessed by CTCAE v4.0  •Clinical Response  •EORTC QLQ-BR23  •EORTC QLQ-C30 | 50 | 18 Years and older (Adult, Older Adult) | Female | •Institut fuer Frauengesundheit  •Merck Sharp & Dohme Corp.  •Celgene Corporation |
| Pembrolizumab | NCT02778685 | Pembrolizumab, Letrozole, and Palbociclib in Treating Postmenopausal Patients With Newly Diagnosed Metastatic Stage IV Estrogen Receptor Positive Breast Cancer | Recruiting | •Estrogen Receptor Positive  •HER2/Neu Negative  •Postmenopausal  •Recurrent Breast Carcinoma  •Stage IV Breast Cancer | •Other: Laboratory Biomarker Analysis  •Drug: Letrozole  •Drug: Palbociclib  •Biological: Pembrolizumab | Interventional | Phase 2 | •Intervention Model: Single Group Assignment  •Masking: None (Open Label)  •Primary Purpose: Treatment | •Response rate (CR or PR) assessed using RECIST version 1.1  •Complete response rate assessed using RECIST version 1.1  •Duration of Response assessed using RECIST version 1.1  •Incidence of adverse events assessed by NCI CTCAE, version 4  •Overall Survival assessed using RECIST version 1.1  •Progression Free Survival assessed using RECIST version 1.1  •Time to treatment failure assessed using irRECIST | 22 | 18 Years and older (Adult, Older Adult) | Female | •City of Hope Medical Center  •National Cancer Institute (NCI) |
| Pembrolizumab | NCT03106415 | Pembrolizumab and Binimetinib in Treating Patients With Locally Advanced or Metastatic Triple Negative Breast Cancer | Recruiting | •Breast Adenocarcinoma  •Estrogen Receptor Negative  •HER2/Neu Negative  •Progesterone Receptor Negative  •Stage III Breast Cancer  •Stage IIIA Breast Cancer  •Stage IIIB Breast Cancer  •Stage IIIC Breast Cancer  •Stage IV Breast Cancer  •Triple-Negative Breast Carcinoma | •Drug: Binimetinib  •Other: Laboratory Biomarker Analysis  •Biological: Pembrolizumab | Interventional | •Phase 1  •Phase 2 | •Intervention Model: Single Group Assignment  •Masking: None (Open Label)  •Primary Purpose: Treatment | •MTD of pembrolizumab in combination with binimetinib using the standard 3+3 design assessed by Common Terminology Criteria for Adverse Events version 4.0 (Phase I)  •ORR as assessed by RECIST (Phase II)  •ORR by irRECIST  •OS  •PFS | 38 | 18 Years and older (Adult, Older Adult) | All | •Mayo Clinic  •National Cancer Institute (NCI) |
| Pembrolizumab | NCT02648477 | Pembrolizumab and Doxorubicin Hydrochloride or Anti-Estrogen Therapy in Treating Patients With Triple-Negative or Hormone Receptor-Positive Metastatic Breast Cancer | Recruiting | •Estrogen Receptor Negative  •Estrogen Receptor Positive  •HER2/Neu Negative  •Progesterone Receptor Negative  •Progesterone Receptor Positive  •Stage IV Breast Cancer  •Triple-Negative Breast Carcinoma | •Drug: Anastrozole  •Drug: Doxorubicin Hydrochloride  •Drug: Exemestane  •Other: Laboratory Biomarker Analysis  •Drug: Letrozole  •Biological: Pembrolizumab | Interventional | Phase 2 | •Allocation: NonRandomized  •Intervention Model: Parallel Assignment  •Masking: None (Open Label)  •Primary Purpose: Treatment | •Incidence of toxicities, assessed by Common Terminology Criteria for Adverse Events, version 4.0 criteria  •Overall response rate in PD-L1 not enriched stage IV breast cancer when combining a previously untested combination of pembrolizumab and aromatase inhibitor (exemestane preferred) (Cohort 2)  •Overall response rate in PD-L1 not enriched stage IV breast cancer when combining a previously untested combination of pembrolizumab and doxorubicin hydrochloride (Cohort 1)  •Clinical benefit rate  •Duration of response, assessed using the RECIST 1.1  •Overall survival (OS), assessed using the RECIST 1.1  •Progression-free survival (PFS), assessed using the RECIST 1.1  •Response, assessed using the RECIST 1.1  •Time-to-treatment failure, assessed using the RECIST 1.1 | 56 | 18 Years and older (Adult, Older Adult) | All | •City of Hope Medical Center  •National Cancer Institute (NCI)  •Merck Sharp & Dohme Corp. |
| Pembrolizumab | NCT02971761 | Pembrolizumab and Enobosarm in Treating Patients With Androgen Receptor Positive Metastatic Triple Negative Breast Cancer | Recruiting | •Androgen Receptor Positive  •Estrogen Receptor Negative  •HER2/Neu Negative  •Progesterone Receptor Negative  •Recurrent Breast Carcinoma  •Stage III Breast Cancer  •Stage IIIA Breast Cancer  •Stage IIIB Breast Cancer  •Stage IIIC Breast Cancer  •Stage IV Breast Cancer  •Metastatic TripleNegative Breast Carcinoma | •Drug: Enobosarm  •Other: Laboratory Biomarker Analysis  •Biological: Pembrolizumab | Interventional | Phase 2 | •Intervention Model: Single Group Assignment  •Masking: None (Open Label)  •Primary Purpose: Treatment | •Dose limiting toxicities assessed by NCI CTCAE v4.0  •Incidence of adverse events (AEs) assessed by National Cancer Institute (NCI) Common Terminology Criteria for Adverse Events (CTCAE) v4.0  •Response rate (CR or PR) assessed using RECIST v1.1  •CBR assessed by irRECIST  •DOR defined as time from documentation of tumor response to disease progression or death in CR or PR patients assessed by irRECIST  •EFS defined as failure of treatment or death as a result of any cause assessed by RECIST v1.1  •OS assessed by RECIST v1.1  •PFS assessed using RECIST v1.1  •TTF defined as time to treatment termination for any reason assessed by RECIST v1.1 | 29 | 18 Years and older (Adult, Older Adult) | All | •City of Hope Medical Center  •National Cancer Institute (NCI) |
| Pembrolizumab | NCT03012230 | Pembrolizumab and Ruxolitinib Phosphate in Treating Patients With Metastatic Stage IV Triple Negative Breast Cancer | Recruiting | •Breast Carcinoma Metastatic in the Bone  •Estrogen Receptor Negative  •HER2/Neu Negative  •Progesterone Receptor Negative  •Recurrent Breast Carcinoma  •Stage IV Breast Cancer  •Triple-Negative Breast Carcinoma | •Other: Laboratory Biomarker Analysis  •Biological: Pembrolizumab  •Drug: Ruxolitinib Phosphate | Interventional | Phase 1 | •Intervention Model: Single Group Assignment  •Masking: None (Open Label)  •Primary Purpose: Treatment | •Incidence of adverse events assessed by NCI CTCAE version 4.0  •MTD defined as the dose level below the lowest dose that induces doselimiting toxicity in at least one-third of patients assessed by National Cancer Institute (NCI) Common Terminology Criteria for Adverse Events (CTCAE) version 4.0  •Best response defined as best objective status recorded from the start of the treatment until disease progression/recurrence assessed by modified RECIST criteria | 18 | 18 Years and older (Adult, Older Adult) | All | •Mayo Clinic  •National Cancer Institute (NCI) |
| Pembrolizumab | NCT03599453 | Chemokine Modulation Therapy and Pembrolizumab in Treating Participants With Metastatic Triple-Negative Breast Cancer | Not yet Recruiting | •Triple -Negative Breast Cancer  •Estrogen Receptor Negative  •HER2/Neu Negative  •Anatomic Stage IV Breast Cancer AJCC  •Progesterone Receptor Negative | •Procedure: Biopsy  •Procedure: Chemokine Modulation Therapy  •Drug: Celecoxib  •Biological: Recombinant Interferon Alfa-2b  •Drug: Rintatolimod  •Biological: Pembrolizumab | Interventional | Phase 2 | •Intervention Model: Single Group Assignment  •Masking: None (Open Label)  •Primary Purpose: Treatment | •Overall response rate (ORR) as measured by immune-related Response Evaluation Criteria in Solid Tumors (irRECIST) criteria 1.1  •Progression-free survival (PFS) as measured by irRECIST 1.1 criteria  •Overall survival (OS) as measured by irRECIST 1.1 criteria  •Disease control rate (DCR) as measured by irRECIST 1.1 criteria  •Incidence of adverse events graded according to Common Terminology Criteria for Adverse Events (CTCAE) version 5.0 | 37 | 18 Years and older (Adult, Older Adult) | Female | •Roswell Park Cancer Institute |
| Pembrolizumab | NCT02779751 | A Study of Abemaciclib (LY2835219) in Participants With Non-Small Cell Lung Cancer or Breast Cancer | Recruiting | •Non Small Cell Lung Cancer  •Breast Cancer | •Drug: Abemaciclib  •Drug: Pembrolizumab  •Drug: Anastrozole | Interventional | Phase 1 | •Allocation: NonRandomized  •Intervention Model: Parallel Assignment  •Masking: None (Open Label)  •Primary Purpose: Treatment | •Number of Participants with One or More Serious Adverse Event(s) (SAEs)  •Number of Participants with Non-Serious Adverse Event(s)  •Objective Response Rate (ORR) per RECIST v1.1: Percentage of Participants With a Complete or Partial Response  •Disease Control Rate (DCR) per RECIST v1.1: Percentage of Participants With a Best Overall Response of Complete Response, Partial Response, and Stable Disease  •Duration of Response (DoR) per RECIST v1.1  •Progression Free Survival (PFS) per RECIST v1.1  •Overall Survival (OS)  •Pharmacokinetics (PK): Mean Steady State Exposure of Abemaciclib  •PK: Mean Steady State Exposure of Pembrolizumab  •PK: Mean Steady State Exposure of Anastrozole | 100 | 18 Years and older (Adult, Older Adult) | All | •Eli Lilly and Company  •Merck Sharp & Dohme Corp. |
| Pembrolizumab | NCT02954874 | Pembrolizumab in Treating Patients With Triple-Negative Breast Cancer | Recruiting | •Estrogen Receptor Negative  •HER2/Neu Negative  •Invasive Breast Carcinoma  •Progesterone Receptor Negative  •Stage 0 Breast Cancer AJCC v6 and v7  •Stage I Breast Cancer AJCC v7  •Stage IA Breast Cancer AJCC v7  •Stage IB Breast Cancer AJCC v7  •Stage II Breast Cancer AJCC v6 and v7  •Stage IIA Breast Cancer AJCC v6 and v7  •and 6 more | •Other: Laboratory Biomarker Analysis  •Other: Patient Observation  •Biological: Pembrolizumab  •Other: Quality-ofLife Assessment  •Other: Questionnaire Administration  •Radiation: Radiation Therapy | Interventional | Phase 3 | •Allocation: Randomized  •Intervention Model: Parallel Assignment  •Masking: None (Open Label)  •Primary Purpose: Treatment | •Invasive disease-free survival (IDFS)  •Severity of fatigue measured by Patient Reported Outcomes Measurement Information System (PROMIS) fatigue scale  •Physical function reported by patients measured by Patient Reported Outcomes Measurement Information System (PROMIS) Global physical health scale  •Overall survival (OS)  •Distant recurrence-free survival (DRFS)  •Incidence of adverse events assessed by National Cancer Institute Common Terminology Criteria for Adverse Events version 4.0  •Severity and frequency of treatment-related symptoms (diarrhea, nausea, rash, cough, and shortness of breath, musculoskeletal pain) over time of patients receiving pembrolizumab  •Emotional function and disease-related symptoms in patients receiving pembrolizumab assessed by Patient Reported Outcomes Measurement Information System (PROMIS) global mental health scale score  •Impact of treatment and treatment-related symptoms on physical function in patients without | 1000 | 18 Years and older (Adult, Older Adult) | All | •National Cancer Institute (NCI |
| Pembrolizumab | NCT03004183 | SBRT and Oncolytic Virus Therapy Before Pembrolizumab for Metastatic TNBC and NSCLC | Recruiting | •Metastatic Nonsmall Cell Lung Cancer  •Metastatic Triplenegative Breast Cancer | •Biological: ADV/ HSV-tk  •Drug: Valacyclovir  •Radiation: SBRT  •Drug: Pembrolizumab | Interventional | Phase 2 | •Intervention Model: Single Group Assignment  •Masking: None (Open Label)  •Primary Purpose: Treatment | •Objective response rate  •Duration of response  •Overall survival rate  •Progression-free survival rate  •Number of participants with treatment-related adverse events  •Antitumor activity  •Clinical benefit rate | 57 | 18 Years and older (Adult, Older Adult) | All | •Jenny C. Chang, MD  •Merck Sharp & Dohme Corp.  •The Methodist Hospital System |
| Pembrolizumab | NCT02395627 | Reversing Therapy Resistance With Epigenetic-Immune Modification | Recruiting | •Breast Neoplasms | •Drug: Tamoxifen  •Drug: Vorinostat  •Drug: pembrolizumab | Interventional | Phase 2 | •Drug: Tamoxifen  •Drug: Vorinostat  •Drug: pembrolizumab | •Overall Response Rate  •Adverse Events  •Progression Free Survival  •Median Progression Free Survival  •Overall Survival  •Tumor Responses  •Response of PD-L1 expression to epigenetic immune priming | 87 | 18 Years and older (Adult, Older Adult) | All | •Pamela Munster  •University of California, San Francisco |
| Pembrolizumab | NCT02957968 | Neoadjuvant Pembrolizumab + Decitabine Followed by Std Neoadj Chemo for Locally Advanced HER2- Breast Ca | Recruiting | •  •Breast Adenocarcinoma  •Estrogen ReceptorNegative Breast Cancer  •Estrogen Receptorpositive Breast Cancer  •HER2/Neu Negative  •Invasive Breast Carcinoma  •Progesterone Receptor Negative  •Progesterone Receptor Positive Tumor  •Stage II Breast Cancer  •Stage IIA Breast Cancer  •Stage IIB Breast Cancer  •and 3 more | •Drug: Doxorubicin  •Drug: Cyclophosphamide  •Drug: Paclitaxel  •Drug: Carboplatin  •Drug: Decitabine  •Drug: Pembrolizumab | Interventional | Phase 2 | •Allocation: NonRandomized  •Intervention Model: Parallel Assignment  •Masking: None (Open Label)  •Primary Purpose: Treatment | •Percent of tumor and stroma with infiltrating lymphocytes from baseline pre-treatment biopsy to post-immunotherapy biopsy following administration of decitabine followed by pembrolizumab.  •All adverse events (AEs) reported during and after immune treatment (ie, decitabine and pembrolizumab)  •Percentage of patients meeting criteria for lymphocyte-predominant breast cancer (LPBC) following treatment with decitabine and pembrolizumab compared to the percentage before treatment.  •Proportion of patients with pathologic complete response (pCR) in the breast and post-therapy lymph nodes.  •Proportion of patients with no or minimal residual disease in the resected breast and axillary specimen.  •The proportion of patients with clinical complete response (cCR)  •Enumeration of T cells and immune cell subsets  •Evaluation of expression of PD-L1 within tumor, stroma, and infiltrating immune cells at baseline and following immunotherapy. | 50 | 18 Years and older (Adult, Older Adult) | All | •Virginia Commonwealth University  •Merck Sharp & Dohme Corp.  •National Cancer Institute (NCI) |
| Pembrolizumab | NCT03428802 | Pembrolizumab in Treating Participants With Metastatic, Recurrent or Locally Advanced Cancer and Genomic Instability | Recruiting | •BRCA1 Gene Mutation  •BRCA2 Gene Mutation  •Locally Advanced Solid Neoplasm  •Metastatic Malignant Solid Neoplasm  •POLD1 Gene Mutation  •POLE Gene Mutation  •Recurrent Malignant Solid Neoplasm  •Recurrent Ovarian Carcinoma  •Stage III Breast Cancer AJCC v7  •Stage III Ovarian Cancer AJCC v8  •and 10 more | •Other: Laboratory Biomarker Analysis  •Biological: Pembrolizumab | Interventional | Phase 2 | •Intervention Model: Single Group Assignment  •Masking: None (Open Label)  •Primary Purpose: Treatment | •Response rate of pembrolizuab  •Progression free survival | 40 | 18 Years and older (Adult, Older Adult) | All | •Rutgers, The State University of New Jersey  •National Cancer Institute (NCI) |
| Pembrolizumab | NCT01042379 | I-SPY 2 TRIAL: Neoadjuvant and Personalized Adaptive Novel Agents to Treat Breast Cancer | Recruiting | •Breast Neoplasms  •Breast Cancer  •Breast Tumors | •Drug: Standard Therapy  •Drug: AMG 386 with or without Trastuzumab  •Drug: AMG 479 (Ganitumab) plus Metformin  •Drug: MK-2206 with or without Trastuzumab  •Drug: AMG 386 and Trastuzumab  •Drug: T-DM1 and Pertuzumab  •Drug: Pertuzumab and Trastuzumab  •Drug: Ganetespib  •Drug: ABT-888  •Drug: Neratinib  •and 9 more | Interventional | Phase 2 | •Allocation: Randomized  •Intervention Model: Parallel Assignment  •Masking: None (Open Label)  •Primary Purpose: Treatment | •Determine whether adding experimental agents to standard neoadjuvant medications increases the probability of pathologic complete response (pCR) over standard neoadjuvant chemotherapy for each biomarker signature established at trial entry.  •Establishing predictive and prognostic indices based on qualification and exploratory markers to predict pCR and residual cancer burden (RCB).  •To determine three- and five-year relapse-free survival (RFS) and OS among the treatment arms.  •To determine incidence of adverse events (AEs), serious adverse events (SAEs), and laboratory abnormalities of each investigational agent tested.  •MRI Volume | 1920 | 18 Years and older (Adult, Older Adult) | Female | •QuantumLeap Healthcare Collaborative |
| Pembrolizumab | NCT03454451 | CPI-006 Alone and in Combination With CPI-444 and With Pembrolizumab for Patients With Advanced Cancers | Recruiting | •Non-Small Cell Lung Cancer  •Renal Cell Cancer  •Colorectal Cancer  •Triple Negative Breast Cancer  •Cervical Cancer  •Ovarian Cancer  •Pancreatic Cancer  •Endometrial Cancer  •Sarcoma  •Squamous Cell Carcinoma of the Head and Neck  •Bladder Cancer  •Metastatic Castration Resistant Prostate Cancer | •Drug: CPI-006  •Drug: CPI-006 + CPI-444  •Drug: CPI-006 + pembrolizumab | Interventional | Phase 1 | •Allocation: Randomized  •Intervention Model: Sequential Assignment  •Masking: None (Open Label)  •Primary Purpose: Treatment | •Incidence of doselimiting toxicities (DLTs) of CPI-006 as a single agent and in combination with CPI-444 and with pembrolizumab.  •Incidence of treatmentemergent adverse events as assessed by NCI CTCAE v.4.03, of CPI-006 as single agent and in combination with CPI-444 and with pembrolizumab.  •Identify the MDL(maximum dose level) of single agent CPI-006  •Area under the curve (AUC) of CPI-006  •Maximum serum concentration (Cmax) of CPI-006  •Objective response rate per RECIST v.1.1 criteria of CPI-006 as single agent and in combination with CPI-444 and with pembrolizumab. | 378 | 18 Years and older (Adult, Older Adult) | All | •Corvus Pharmaceuticals, Inc. |
| Pembrolizumab | NCT03432741 | Direct Tumor Microinjection and FDG-PET in Testing Drug Sensitivity in Patients With Relapsed or Refractory NonHodgkin Lymphoma, Hodgkin Lymphoma, or Stage IV Breast Cancer | Recruiting | •Breast Adenocarcinoma  •Recurrent Breast Carcinoma  •Recurrent Hodgkin Lymphoma  •Recurrent Mycosis Fungoides  •Recurrent Non-Hodgkin Lymphoma  •Recurrent Primary Cutaneous TCell Non-Hodgkin Lymphoma  •Refractory Hodgkin Lymphoma  •Refractory Mycosis Fungoides  •Refractory Nodal Marginal Zone Lymphoma  •Refractory Non-Hodgkin Lymphoma  •Refractory Primary Cutaneous TCell Non-Hodgkin Lymphoma  •Stage IV Breast Cancer AJCC v6 and v7 | •Drug: Belinostat  •Drug: Carfilzomib  •Biological: Daratumumab  •Drug: Fludeoxyglucose F-18  •Drug: Gemcitabine Hydrochloride  •Other: Laboratory Biomarker Analysis  •Biological: Nivolumab  •Biological: Obinutuzumab  •Biological: Pembrolizumab  •Procedure: Positron Emission Tomography  •and 4 more | Interventional | Phase 1 | •Intervention Model: Single Group Assignment  •Masking: None (Open Label)  •Primary Purpose: Treatment | •Incidence of drug sensitivity as measured by injection site skin reaction  •Cutaneous response rate based upon the modified Severity Weighted Assessment Tool score  •Feasibility defined as at least 70% of the enrolled patients complete the injection and response evaluation  •Incidence of adverse events  •Nodal disease response rate | 26 | 18 Years and older (Adult, Older Adult) | All | •Mayo Clinic  •National Cancer Institute (NCI) |
| Pembrolizumab | NCT03435952 | Pembrolizumab With Intratumoral Injection of Clostridium Novyi-NT | Recruiting | •Malignant Neoplasm of Breast  •Malignant Neoplasms of Digestive Organs  •Malignant Neoplasms of Eye Brain and Other Parts of Central Nervous System  •Malignant Neoplasms of Female Genital Organs  •Malignant Neoplasms of Illdefined Secondary and Unspecified Sites  •Malignant Neoplasms of Independent (Primary) Multiple Sites  •Malignant Neoplasms of Lip Oral Cavity and Pharynx  •Malignant Neoplasms of Male Genital Organs  •Malignant Neoplasms of Mesothelial and Soft Tissue  •Malignant Neoplasms of Respiratory and Intrathoracic Organs  •Malignant Neoplasms of Thyroid and Other Endocrine Glands  •Malignant Neoplasms of Urinary Tract | •Drug: Pembrolizumab  •Biological: Clostridium NovyiNT  •Drug: Doxycycline | Interventional | Phase 1 | •Intervention Model: Single Group Assignment  •Masking: None (Open Label)  •Primary Purpose: Treatment | •Maximum Tolerated Dose (MTD) of Intratumoral Injection of Clostridium Novyi-NT with Pembrolizumab  •Preliminary Anti-Tumor Activity of Pembrolizumab in Combination with C. novyi-NT in the Injected Tumor and an Overall Response by RECIST 1.1 | 18 | 18 Years and older (Adult, Older Adult) | All | •M.D. Anderson Cancer Center  •BioMed Valley Discoveries, Inc  •Merck Sharp & Dohme Corp.  •Other  •Industry Study Start: July 10, 2018 Primary Completion: October 2020 Study Complet |
| Pembrolizumab | NCT02646748 | Pembrolizumab Combined With Itacitinib (INCB039110) and/or Pembrolizumab Combined With INCB050465 in Advanced Solid Tumors | Recruiting | •Colorectal Cancer (CRC)  •Endometrial Cancer  •Melanoma  •Head and Neck Cancer  •Lung Cancer  •MMR-deficient Tumors  •Breast Cancer  •Pancreatic Cancer  •Renal Cell Carcinoma (RCC)  •Solid Tumors  •UC (Urothelial Cancer) | •Drug: Pembrolizumab  •Drug: itacitinib  •Drug: INCB050465 | Interventional | Phase 1 | •Allocation: NonRandomized  •Intervention Model: Parallel Assignment  •Masking: None (Open Label)  •Primary Purpose: Treatment | •Evaluation of safety and tolerability as measured by the frequency, duration, and severity of adverse events  •Objective Response Rate (ORR) as determined by radiographic disease assessments per immunerelated Response Evaluation Criteria In Solid Tumors (irRECIST) v1.1 criteria  •Progression Free Survival (PFS) as measured by the duration from the date of first dose until the earliest date of disease progression or death as measured by immune-related Response Evaluation Criteria In Solid Tumors (irRECIST) v1.1  •Duration of response (DOR) as measured from the time of the earliest response complete response (CR) or partial response (PR) until disease progression per irRECIST v1.1 | 237 | 18 Years and older (Adult, Older Adult) | All | •Incyte Corporation |
| Pembrolizumab | NCT02952989 | A Safety Study of SGN-2FF for Patients With Advanced Solid Tumors | Recruiting | •Carcinoma, NonSmall-Cell Lung  •Carcinoma, Renal Cell  •Breast Neoplasms  •Urinary Bladder Neoplasm  •Carcinoma, Squamous Cell of Head and Neck  •Colorectal Neoplasms  •Gastric Adenocarcinoma  •Gastroesophageal Junction Adenocarcinoma | •Drug: SGN-2FF  •Drug: pembrolizumab | Interventional | Phase 1 | •Allocation: NonRandomized  •Intervention Model: Parallel Assignment  •Masking: None (Open Label)  •Primary Purpose: Treatment | •The number of participants with adverse events that are related to treatment  •The number of participants with laboratory abnormalities that are related to treatment  •Incidence of dose-limiting toxicities (DLTs)  •Pharmacokinetic assessments  •Markers of fucosylation status  •Objective response rate  •Disease control rate  •Duration of response  •Clinical benefit rate  •Progression-free survival  •Overall survival | 308 | 18 Years and older (Adult, Older Adult) | All | •Seattle Genetics, Inc. |
| Pembrolizumab | NCT01174121 | Immunotherapy Using Tumor Infiltrating Lymphocytes for Patients With Metastatic Cance | Recruiting | •Metastatic Colorectal Cancer  •Metastatic Gastric Cancer  •Metastatic Pancreatic Cancer  •Metastatic Hepatocellular Carcinoma  •Progressive Glioblastoma  •Metastatic Ovarian Cancer  •Metastatic Breast Cancer | •Biological: Young TIL  •Drug: Aldesleukin  •Drug: Cyclophosphamide  •Drug: Fludarabine  •Drug: Pembrolizumab | Interventional | Phase 2 | •Allocation: NonRandomized  •Intervention Model: Parallel Assignment  •Masking: None (Open Label)  •Primary Purpose: Treatment | •Response rate  •Frequency and severity of treatment-related to adverse events  •Safety and efficacy of pembrolizumab following TIL therapy | 332 | 18 Years to 70 Years (Adult, Older Adult) | All | •National Cancer Institute (NCI)  •National Institutes of Health Clinical Center (CC) |
| Pembrolizumab | NCT03674567 | Dose Escalation and Expansion Study of FLX475 Monotherapy and in Combination With Pembrolizumab | Recruiting | •Advanced Cance | •Drug: FLX475  •Drug: Pembrolizumab | Interventional | •Phase 1  •Phase 2 | •Allocation: NonRandomized  •Intervention Model: Sequential Assignment  •Masking: None (Open Label)  •Primary Purpose: Treatment | •Safety and tolerability of FLX475 as a single agent and in combination with pembrolizumab measured by the incidence of adverse events, including dose-limiting toxicities and maximum tolerated dose  •Overall response rate in subjects treated with FLX475 as a single agent and in combination with pembrolizumab | 375 | 18 Years and older (Adult, Older Adult) | All | •FLX Bio, Inc. |
| Pembrolizumab | NCT02872025 | Pembrolizumab in High-risk Ductal Carcinoma in Situ (DCIS) | Recruiting | •Drug: Pembrolizumab | •Drug: Carboplatin  •Drug: Nabpaclitaxel  •Drug: Pembrolizumab | Interventional | Early Phase 1 | •Allocation: Randomized  •Intervention Model: Parallel Assignment  •Masking: None (Open Label)  •Primary Purpose: Treatment | •Maximum tolerated dose (MTD)  •Dose-limiting toxicities (DLTs)  •Percentage of patients who demonstrate an increase (baseline vs. post intralesional injection) in intralesional CD8+ T cells (treated vs. untreated participants) as measured using multiplex immunofluorescence on FFPE tissue sections | 48 | 18 Years and older (Adult, Older Adult) | Female | •Laura Esserman  •Merck Sharp & Dohme Corp.  •University of California, San Francisco |
| Pembrolizumab | NCT02393248 | Open-Label, Dose-Escalation Study of INCB054828 in Subjects With Advanced Malignancies - (FIGHT-101) | Recruiting | •Lung Cancer  •Solid Tumor  •Gastric Cancer  •UC (Urothelial Cancer)  •Endometrial Cancer  •Multiple Myeloma  •MPN (Myeloproliferative Neoplasms)  •Breast Cancer  •Cholangiocarcinoma | •Drug: INCB054828  •Drug: Gemcitabine + Cisplatin  •Drug: Pembrolizumab  •Drug: Docetaxel  •Drug: Trastuzumab | Interventional | •Phase 1  •Phase 2 | •Intervention Model: Single Group Assignment  •Masking: None (Open Label)  •Primary Purpose: Treatment | •Determination of the maximum tolerated dose of INCB054828 as a monotherapy and in combination as measured by the number of participants with adverse events  •Assess the pharmacodynamics of INCB054828 as a monotherapy and in combination as indicated by serum phosphorus level  •Preliminary efficacy as assessed by Overall Response Rate (ORR) of INCB054828 as monotherapy and in combination in subjects with measurable disease  •Maximum observed plasma concentration (Cmax) during the dosing interval and Cmin of INCB054828 as monotherapy and in combination  •Minimum observed plasma concentration (Cmin) during the dosing interval of INCB054828 as monotherapy and in combination  •Time to maximum plasma concentration (Tmax) of INCB054828 as monotherapy and in combination  •Area under the single-dose plasma concentrationtime curve (AUC0- t) of INCB054828 as monotherapy and in combination  •Oral | 280 | 18 Years and older (Adult, Older Adult) | All | •Incyte Corporation |
| Pembrolizumab | NCT02890368 | Trial of Intratumoral Injections of TTI-621 in Subjects With Relapsed and Refractory Solid Tumors and Mycosis Fungoides | Recruiting | •Solid Tumors  •Mycosis Fungoides  •Melanoma  •Merkel-cell Carcinoma  •Squamous Cell Carcinoma  •Breast Carcinoma  •Human PapillomavirusRelated Malignant Neoplasm  •Soft Tissue Sarcoma | •Drug: TTI-621 Monotherapy  •Drug: TTI-621 + PD-1/PD-L1 Inhibitor  •Drug: TTI-621 + pegylated interferon-#2a  •Other: TTI-621 + TVec  •Other: TTI-621 + radiation | Interventional | Phase 1 | •Allocation: NonRandomized  •Intervention Model: Parallel Assignment  •Masking: None (Open Label)  •Primary Purpose: Treatment | •Optimal TTI-621 delivery regimen  •Frequency and severity of adverse events  •Preliminary evidence of anti-tumor activity of TTI-621 | 240 | 18 Years and older (Adult, Older Adult) | All | •Trillium Therapeutics Inc. |
| Nivolumab | NCT03414684 | Carboplatin +/- Nivolumab in Metastatic Triple Negative Breast Cancer | Recruiting | •Breast Cancer | •Drug: Carboplatin  •Drug: Nivolumab | Interventional | Phase 2 | •Allocation: Randomized  •Intervention Model: Parallel Assignment  •Masking: None (Open Label)  •Primary Purpose: Treatment | •Progression-free survival (PFS)  •Overall Response Rate  •Overall Survival  •Clinical Benefit Rate  •Duration of Response  •Time to Objective Response  •BRCA carriers  •Incidence Rate of each Toxicity (safety and tolerability) | 132 | 18 Years and older (Adult, Older Adult) | All | •Dana-Farber Cancer Institute  •Bristol-Myers Squibb |
| Nivolumab | NCT03316586 | A Phase II Study of Nivolumab in Combination With Cabozantinib for Metastatic Triple-negative Breast Cancer | Recruiting | • Breast Cancer | •Drug: Nivolumab  •Drug: Cabozantinib | Interventional | Phase 2 | •Intervention Model: Single Group Assignment  •Masking: None (Open Label)  •Primary Purpose: Treatment | •Overall Response Rate  •Number of participants with adverse events  •Clinical Benefit Rate  •Progression Free Survival Rate  •Overall Response Rate per Immune Criteria | 35 | 18 Years and older (Adult, Older Adult) | Female | •Dana-Farber Cancer Institute  •Bristol-Myers Squibb  •Exelixis |
| Nivolumab | NCT03546686 | Peri-Operative Ipilimumab +Nivolumab and Cryoablation Versus Standard Care in Women With Triple-negative Breast Cancer | Not yet Recruiting | • Breast Cancer | •Drug: Ipilimumab  •Drug: Nivolumab  •Procedure: Core Biopsy/ Cryoablation  •Procedure: Breast Surgery | Interventional | Phase 2 | •Allocation: Randomized  •Intervention Model: Parallel Assignment  •Masking: None (Open Label)  •Primary Purpose: Treatment | •Distant Disease-Free Survival  •Invasive Disease-Free Survival  •Disease-Free Survival  •Overall Survival  •Overall Safety | 150 | 18 Years and older (Adult, Older Adult) | Female | •Cedars-Sinai Medical Center |
| Nivolumab | NCT03742986 | Trial of Nivolumab With Chemotherapy as Neoadjuvant Treatment in Inflammatory Breast Cancer (IBC) | Not yet Recruiting | • Breast Cancer | •Drug: Nivolumab 360 mg+ paclitaxel 80 mg/m2  •Drug: Doxorubicin 60 mg/ m2+Cyclophosphamide 600 mg/m2  •Drug: Nivolumab 360 mg+ Docetaxel* 75 mg/ m2 +Trastuzumab 8 mg/kg +Pertuzumab 840 mg  •Drug: Doxorubicin 60 mg/m2 +Cyclophosphamide 600 mg/m2 | Interventional | Phase 2 | •Allocation: NonRandomized  •Intervention Model: Parallel Assignment  •Masking: None (Open Label)  •Primary Purpose: Treatment | Change in pathological complete response (pCR) | 52 | 18 Years and older (Adult, Older Adult) | All | •New York University School of Medicine |
| Nivolumab | NCT02393794 | Cisplatin Plus Romidepsin & Nivolumab in Locally Recurrent or Metastatic Triple Negative Breast Cancer (TNBC) | Recruiting | •Triple-Negative Breast Cancer  •Breast Cancer | •Drug: Romidepsin  •Drug: Cisplatin  •Drug: Nivolumab | Interventional | •Phase 1  •Phase 2 | •Allocation: NonRandomized  •Intervention Model: Single Group Assignment  •Masking: None (Open Label)  •Primary Purpose: Treatment | •Phase I: Recommended Phase II Dose of romidepsin in combination with cisplatin  •Phase II: Objective response rate of treated subjects according to RECIST v1.1 criteria  •Phase II: Clinical Benefit Rate at 16 weeks of study treatment for subjects treated at the recommended phase II dose of romidepsin plus cisplatin and nivolumab  •Pharmacokinetics - romidepsin plasma concentration vs time profile when given with cisplatin and nivolumab  •Pharmacokinetics - cisplatin plasma concentration vs time profile when given with romidepsin  •Median ProgressionFree Survival and Overall Survival | 54 | 18 Years and older (Adult, Older Adult) | Female | •Priyanka Sharma  •Celgene Corporation  •Bristol-Myers Squibb  •University of Kansas Medical Center |
| Nivolumab | NCT03409198 | Phase IIb Study Evaluating Immunogenic Chemotherapy Combined With Ipilimumab and Nivolumab in Breast Cancer | Recruiting | •Breast Cancer  •Luminal B | •Drug: Ipilimumab  •Drug: Nivolumab  •Drug: Pegylated liposomal doxorubicin  •Drug: Cyclophosphamide | Interventional | Phase 2 | •Allocation: Randomized  •Intervention Model: Parallel Assignment  •Masking: None (Open Label)  •Primary Purpose: Treatment | •Toxicity: CTCAE v4.0  •Progression-free survival (PFS)  •Duration of Response (DR)  •Overall Survival (OS)  •Duration of Response (DR) in cross-over arm  •Overall Suvival (OS) in cross-over arm  •Toxicity, cross-over arm, CTCAE v4.0  •Objective tumor Response Rate (ORR)  •Durable tumor Response Rate (DRR)  •Objective tumor Response Rate (ORR) in cross-over arm  •and 7 more | 75 | 18 Years and older (Adult, Older Adult) | All | •Oslo University Hospital  •Bristol-Myers Squibb  •Helse Stavanger HF  •Helse Sor-Ost  •Sorlandet Hospital HF |
| Nivolumab | NCT03650894 | Nivolumab, Ipilimumab, and Bicalutamide in Human Epidermal Growth Factor (HER) 2 Negative Breast Cancer Patients | Not yet Recruiting | •Breast Neoplasm Female  •Breast Cancer  •Breast Carcinoma  •Breast Tumor | •Drug: Nivolumab  •Drug: Ipilimumab  •Drug: Bicalutamide | Interventional | Phase 2 | •Intervention Model: Single Group Assignment  •Masking: None (Open Label)  •Primary Purpose: Treatment | •iRECIST Clinical Benefit Rate (the number of patients with objective response or ongoing stable disease at week 24 using iRECIST guidelines)  •RECIST Clinical Benefit Rate (the number of patients who have a response according to RECIST criteria at week 12) | 138 | 18 Years and older (Adult, Older Adult) | Female | •Providence Health & Services  •Bristol-Myers Squibb  •Memorial Sloan Kettering Cancer Center |
| Nivolumab | NCT02499367 | Nivolumab After Induction Treatment in Triple-negative Breast Cancer (TNBC) Patients | Recruiting | • Breast Cancer | •Drug: Nivolumab  •Radiation: Radiation therapy  •Drug: Low dose doxorubicin  •Drug: Cyclophosphamide  •Drug: Cisplatin | Interventional | Phase 2 | •Allocation: Randomized  •Intervention Model: Parallel Assignment  •Masking: None (Open Label)  •Primary Purpose: Treatment | •Progression free survival  •Overall response rate  •Clinical benefit rate  •Toxicity o | 84 | 18 Years and older (Adult, Older Adult) | All | •The Netherlands Cancer Institute  •Bristol-Myers Squibb |
| Nivolumab | NCT02892734 | Ipilimumab and Nivolumab in Treating Patients With Recurrent Stage IV HER2 Negative Inflammatory Breast Cancer | Recruiting | •HER2/Neu Negative  •Recurrent Inflammatory Breast Carcinoma  •Stage IV Breast Cancer  •Stage IV Inflammatory Breast Carcinoma | •Biological: Ipilimumab  •Other: Laboratory Biomarker Analysis  •Biological: Nivolumab | Interventional | Phase 2 | •Intervention Model: Single Group Assignment  •Masking: None (Open Label)  •Primary Purpose: Treatment | •Progression Free Survival (PFS)  •Overall Response Rate (ORR)  •Clinical Benefit Rate (CBR)  •Overall survival  •Incidence of Adverse Events | 29 | 18 Years and older (Adult, Older Adult) | Female | •Northwestern University  •Bristol-Myers Squibb  •National Cancer Institute (NCI) |
| Nivolumab | NCT02833233 | A Study of Pre-Operative Treatment With Cryoablation and Immune Therapy in Early Stage Breast Cancer | Active, not recruiting | • Breast Cancer | •Procedure: Cryoablation  •Drug: Ipilimumab  •Drug: Nivolumab | Interventional | Not Applicable | •Intervention Model: Single Group Assignment  •Masking: None (Open Label)  •Primary Purpose: Treatment | Number of adverse events | 5 | 18 Years and older (Adult, Older Adult) | Female | •Memorial Sloan Kettering Cancer Center  •Bristol-Myers Squibb |
| Nivolumab | NCT02309177 | Safety Study of Nivolumab With Nab-Paclitaxel Plus or Minus Gemcitabine in Pancreatic Cancer, Nab-Paclitaxel / Carboplatin in Stage IIIB/IV Non-Small Cell Lung Cancer or Nab-Paclitaxel in Recurrent Metastatic Breast Cancer | Completed | •Breast Neoplasms  •Pancreatic Neoplasms | •Drug: nabPaclitaxel  •Drug: Nivolumab  •Drug: Gemcitabine  •Drug: Carboplatin | Interventional | Phase 1 | •Allocation: Randomized  •Intervention Model: Parallel Assignment  •Masking: None (Open Label)  •Primary Purpose: Treatment | •Evaluate Dose Limiting Toxicity (DLT) of each combination regimen  •Evaluate the safety of the nab-paclitaxel/nivolumab combination regimens  •Grade 3 or 4 TEAE  •Treatment Emergent Adverse Events  •Progression-free survival  •Overall Survival  •Disease Control Rate  •Overall Response Rate  •Duration of Response | 114 | 18 Years and older (Adult, Older Adult) | All | •Celgene |
| Nivolumab | NCT03487666 | OXEL: Pilot Study of Immune Checkpoint or Capecitabine or Combination Therapy as Adjuvant Therapy for TNBC With Residual Disease | Recruiting | •Triple Negative Breast Cancer | •Drug: Nivolumab  •Drug: Capecitabine | Interventional | Phase 2 | •Allocation: Randomized  •Intervention Model: Parallel Assignment  •Masking: None (Open Label)  •Primary Purpose: Treatment | •Immune activation measured by changes in the peripheral immunoscore (PIS) at week 6  •Immune activation measured by changes of PIS at week 12  •Grade 3 and 4 toxicities according to the National Cancer Institute Common Toxicity Criteria for Adverse Events Version 4.0 [NCI CTCAE v4.03]  •Distant recurrence free survival (DRFS) and Overall Survival  •Immune activation in the tumor by IHC  •Immune activation in the tumor by flow cytometry  •Immune activation in the tumor by ELISA  •Intracellular cytokine staining and CD8+ T-cell clonal expansion  •Circulating tumor DNA | 45 | 18 Years and older (Adult, Older Adult) | All | •Georgetown University  •Bristol-Myers Squibb |
| Nivolumab | NCT03342417 | Combination of Nivolumab and Ipilimumab in Breast, Ovarian and Gastric Cancer Patients | Recruiting | •Breast Cancer Female  •Ovarian Cancer  •Gastric Cancer | •Biological: Nivolumab  •Biological: Ipilimumab | Interventional | Phase 2 | •Allocation: NonRandomized  •Intervention Model: Single Group Assignment  •Masking: None (Open Label)  •Primary Purpose: Treatment | •Number of participants with treatment-related adverse events as assessed by CTCAE v4.0  •Clinical Response  •Part 1 of the Study (Neoadjuvant Therapy of Breast Cancer) - BCT  •Part 1 of the Study (Neoadjuvant Therapy of Breast Cancer) - Possible predictive biomarkers  •Part 1 of the Study (Neoadjuvant Therapy of Breast Cancer) - Status of PD-L1, PDL-2, and PD-1 in tumor tissues before vs. after investigational neoadjuvant therapy  •Part 1 of the Study (Neoadjuvant Therapy of Breast Cancer) - Possible transition of Th2 to Th1 in the tumor  •Part 1 of the Study (Neoadjuvant Therapy of Breast Cancer) - BOR and ORR  •Part 1 of the Study (Neoadjuvant Therapy of Breast Cancer) - PFS  •Part 2 of the Study (Therapy of Ovarian Cancer) and Part 3 of the Study (Therapy of Gastric Cancer) - DOR  •Part 2 of the Study (Therapy of Ovarian Cancer) and Part 3 of the Study (Therapy of Gastric Cancer) - OS  •Part 2 of the Study (Therapy of Ovarian | 60 | 18 Years and older (Adult, Older Adult) | All | •ExcellaBio LLC |
| Nivolumab | NCT03098550 | A Study to Test the Safety and Effectiveness of Nivolumab Combined With Daratumumab in Patients With Pancreatic, Non-Small Cell Lung or Triple Negative Breast Cancers, That Have Advanced or Have Spread | Active, not recruiting | •Advanced Cancer | •Biological: Nivolumab  •Biological: Daratumumab | Interventional | •Phase 1  •Phase 2 | •Intervention Model: Single Group Assignment  •Masking: None (Open Label)  •Primary Purpose: Treatment | •Incidence of adverse events (AE)  •Incidence of serious adverse events (SAE)  •Grade of laboratory abnormalities.  •Objective Response rate (ORR)  •Progression Free Survival (PFS)  •Anti-Drug Antibodies (ADA) positivity  •Area under the concentration-time curve (AUC)  •Minimum observed concentration (Cmin) | 120 | 18 Years and older (Adult, Older Adult) | All | •Bristol-Myers Squibb  •Janssen Biotech, Inc |
| Nivolumab | NCT02453620 | Entinostat, Nivolumab, and Ipilimumab in Treating Patients With Solid Tumors That Are Metastatic or Cannot Be Removed by Surgery or Locally Advanced or Metastatic HER2- Negative Breast Cancer | Recruiting | •Breast Adenocarcinoma  •HER2/Neu Negative  •Invasive Breast Carcinoma  •Metastatic Malignant Solid Neoplasm  •Stage III Breast Cancer AJCC v7  •Stage IIIA Breast Cancer AJCC v7  •Stage IIIB Breast Cancer AJCC v7  •Stage IIIC Breast Cancer AJCC v7  •Stage IV Breast Cancer AJCC v6 and v7  •Unresectable Solid Neoplasm | •Drug: Entinostat  •Biological: Ipilimumab  •Other: Laboratory Biomarker Analysis  •Biological: Nivolumab  •Other: Pharmacogenomic Study  •Other: Pharmacological Study | Interventional | Phase 1 | •Intervention Model: Single Group Assignment  •Masking: None (Open Label)  •Primary Purpose: Treatment | •Incidence of adverse events of entinostat and nivolumab in combination with ipilimumab per National Cancer Institute Common Terminology Criteria for Adverse Events (CTCAE) version (v)5.0  •Changes in ratio of effector T cell (Teff) to regulatory T cell (Treg) in tumor biopsies, measured by immunohistochemistry (IHC) staining of paraffin embedded tumor specimens  •Objective response rate, defined as the total number of patients with either complete response (CR) or partial response (PR) divided by the total number of patients in the population of interest (expansion cohort of patients with advanced breast cancer)  •Disease control rate (expansion cohort of patients with advanced breast cancer)  •Progression-free survival (PFS), defined as the proportion of patients remaining alive and free of disease progression (expansion cohort of patients with advanced breast cancer)  •Duration of overall response (expansion cohort of patients with advanced breast cancer)  •Duration of stable disease based on Response Evaluation Criteria in Solid Tumors (RECIST) version | 45 | 18 Years and older (Adult, Older Adult) | All | •National Cancer Institute (NCI) |
| Nivolumab | NCT03523572 | Trastuzumab Deruxtecan (DS-8201a) With Nivolumab in Advanced Breast and Urothelial Cancer | Recruiting | •Breast Cancer  •Urothelial Carcinoma | •Drug: Trastuzumab deruxtecan (DS-8201a)  •Drug: Nivolumab | Interventional | Phase 1 | •Allocation: NonRandomized  •Intervention Model: Sequential Assignment  •Masking: None (Open Label)  •Primary Purpose: Treatment | •Number of participants with dose-limiting toxicity at each dose level  •Part 2: Dose expansion - Objective response rate (ORR)  •Duration of Response (DoR)  •Disease Control Rate (DCR)  •Progression Free Survival (PFS)  •Time to Response based on central review  •Overall Survival (OS)  •ORR | 99 | 18 Years and older (Adult, Older Adult) | All | •Daiichi Sankyo, Inc.  •Bristol-Myers Squibb |
| Nivolumab | NCT02983045 | A Dose Escalation and Cohort Expansion Study of CD122- Biased Cytokine (NKTR-214) in Combination With Anti-PD-1 Antibody (Nivolumab) or in Combination With Nivolumab and Anti-CTLA4 Antibody (Ipilimumab) in Patients With Select Advanced or Metastatic Solid Tumors | Recruiting | •Melanoma  •Renal Cell Carcinoma  •Non Small Cell Lung Cancer  •Urothelial Carcinoma  •Triple Negative Breast Cancer | •Drug: Combination of NKTR-214 + nivolumab  •Drug: Combination of NKTR-214 + nivolumab + ipilimumab | Interventional | •Phase 1  •Phase 2 | •Allocation: NonRandomized  •Intervention Model: Parallel Assignment  •Masking: None (Open Label)  •Primary Purpose: Treatment | •Safety of NKTR-214 in combination with nivolumab as evaluated by incidence of drugrelated Adverse Events (AEs), Serious Adverse Events (SAEs), and adverse events leading to discontinuation, deaths, and clinical laboratory test abnormalities  •Safety of NKTR-214 in combination with nivolumab and ipilmumab as evaluated by incidence of drug-related AEs, SAEs, and adverse events leading to discontinuation, deaths, and clinical laboratory test abnormalities  •Tolerability of NKTR-214 in combination with nivolumab as evaluated by incidence of Dose Limiting Toxicities (DLTs), drug-related AEs, SAEs, adverse events leading to discontinuation, deaths and clinical laboratory test abnormalities  •Tolerability of NKTR-214 in combination with nivolumab and ipilmumab as evaluated by incidence of Dose Limiting Toxicities (DLTs), drug-related AEs, SAEs, adverse events leading to discontinuation, deaths and clinical laboratory test abnormalities  •Efficacy of NKTR-214 in combination with nivolumab | 480 | 18 Years and older (Adult, Older Adult) | All | •Nektar Therapeutics  •Bristol-Myers Squibb |
| Nivolumab | NCT02834247 | A Study of TAK-659 in Combination With Nivolumab in Participants With Advanced Solid Tumors | Recruiting | •Triple-Negative Breast Neoplasms  •Carcinoma, NonSmall-Cell Lung  •Head and Neck Carcinoma, Squamous Cell  •Advanced Solid Tumors | •Drug: TAK-659  •Drug: Nivolumab | Interventional | Phase 1 | •Allocation: NonRandomized  •Intervention Model: Parallel Assignment  •Masking: None (Open Label)  •Primary Purpose: Treatment | •Part 1: Maximum Tolerated Dose (MTD)  •Part 1: RP2D  •Part 2: Overall Response Rate (ORR)  •Percentage of Participants Experiencing 1 or More Treatment-Emergent Adverse Events (TEAEs)  •Percentage of Participants with 1 or More Grade 3 and Grade 4 Adverse Events (AEs)  •Percentage of Participants Experiencing Serious Adverse Events (SAEs)  •Percentage of Participants With TEAEs Resulting in Study Drug Discontinuation  •Number of Participants With Clinically Significant Laboratory Values  •Number of Participants with Clinically Significant Vital Sign Measurements  •Part 2: Percentage of Participants With Disease Control  •and 7 more | 126 | 18 Years and older (Adult, Older Adult) | All | •Millennium Pharmaceuticals, Inc.  •Takeda |
| Nivolumab | NCT03430479 | Anti PD-1 Antibody With Radiation Therapy in Patients With HER2-negative Metastatic Breast Cancer | Recruiting | • Breast Cancer | •Drug: Cohort A | Interventional | •Phase 1  •Phase 2 | •Allocation: NonRandomized  •Intervention Model: Single Group Assignment  •Masking: Double (Participant, Care Provider)  •Primary Purpose: Treatment | Phase Ib : dose-limiting toxicity rate | 32 | 20 Years and older (Adult, Older Adult) | Female | •Kyoto Breast Cancer Research Network |
| Nivolumab | NCT03326258 | Glembatumumab Vedotin, Nivolumab, and Ipilimumab in Treating Patients With Advanced Metastatic Solid Tumors That Cannot Be Removed by Surgery | Withdraw | •Advanced Malignant Solid Neoplasm  •Estrogen Receptor Negative  •GPNMB Positive  •HER2/Neu Negative  •Metastatic Malignant Solid Neoplasm  •Metastatic Melanoma  •Progesterone Receptor Negative  •Stage III Breast Cancer AJCC v7  •Stage III Cutaneous Melanoma AJCC v7  •Stage III Uveal Melanoma AJCC v7  •and 8 more | •Drug: Glembatumumab Vedotin  •Biological: Ipilimumab  •Other: Laboratory Biomarker Analysis  •Biological: Nivolumab  •Other: Pharmacological Study | Interventional | •Phase 1  •Phase 2 | •Intervention Model: Single Group Assignment  •Masking: None (Open Label)  •Primary Purpose: Treatment | •Recommended phase 2 dose for the combination of glembatumumab vedotin and nivolumab (Phase Ib)  •Antitumor activity measured by ImmuneModified Response Evaluation Criteria in Solid Tumors (iRECIST)/ Response Evaluation Criteria in Solid Tumors (RECIST) 1.1 (Phase II)  •Incidence of adverse events  •Overall response rate  •Clinical benefit rate  •Progression free survival  •Overall survival  •Pharmacokinetic parameters  •Levels of plasma and tissue biomarkers | 0 | 18 Years and older (Adult, Older Adult) | All | •National Cancer Institute (NCI) |
| Nivolumab | NCT03435640 | A Study of NKTR-262 in Combination With NKTR-214 and With NKTR-214 Plus Nivolumab in Patients With Locally Advanced or Metastatic Solid Tumor Malignancies | Recruiting | •Melanoma  •Merkel Cell Carcinoma  •Triple Negative Breast Cancer  •Ovarian Cancer  •Renal Cell Carcinoma  •Colorectal Cancer  •Urothelial Carcinoma  •Sarcoma | •Drug: NKTR-262  •Drug: NKTR-214  •Drug: nivolumab | Interventional | •Phase 1  •Phase 2 | •Allocation: NonRandomized  •Intervention Model: Parallel Assignment  •Masking: None (Open Label)  •Primary Purpose: Treatment | •Safety of NKTR-262 in combination with NKTR-214 / nivolumab as evaluated by incidence of drug-related Adverse Events (AEs), Serious Adverse Events (SAEs), and AEs leading to discontinuation, deaths, and clinical laboratory abnormalities per CTCAE 4.03  •Tolerability of NKTR-262 in combination with NKTR-214 / nivolumab as evaluated by incidence of Dose Limiting Toxicities (DLTs), drug-related AEs, SAEs, AEs leading to discontinuation, deaths, clinical laboratory abnormalities per CTCAE 4.03  •Efficacy of NKTR-262 in combination with NKTR-214 / nivolumab as assessed by the Objective Response Rate (ORR) based on RECIST 1.1 | 393 | 18 Years and older (Adult, Older Adult) | All | •Nektar Therapeutics |
| Nivolumab | NCT02637531 | A Dose-Escalation Study to Evaluate the Safety, Tolerability, Pharmacokinetics, and Pharmacodynamics of IPI-549 | Recruiting | •Advanced Solid Tumors (Part A/B/ C/D)  •Non-small Cell Lung Cancer (Part E)  •Melanoma (Part E)  •Squamous Cell Cancer of the Head and Neck (Part E)  •Triple Negative Breast Cancer (Part F)  •Adrenocortical Carcinoma (Part G)  •Mesothelioma (Part G)  •High-circulating Myeloid-derived Suppressor Cells (Part H) | •Drug: IPI-549  •Drug: Nivolumab | Interventional | Phase 1 | •Allocation: NonRandomized  •Intervention Model: Single Group Assignment  •Masking: None (Open Label)  •Primary Purpose: Treatment | •Part A/B/C: Dose Limiting Toxicities (DLT)  •Part D/E: Adverse Events (AE) and safety laboratory values  •Part A/B: Adverse Events (AE) and safety laboratory values  •Part A/B: Plasma concentrations of IPI-549 (metabolites, as appropriate)  •Part A/B: Overall response rate (ORR), complete response/remission (CR) or partial response/ remission (PR)  •Part A/B: Duration of response (DoR)  •Part C: Adverse Events (AE) and safety laboratory values  •Part C: Plasma concentrations of IPI-549 (metabolites as appropriate)  •Part C: Overall Response Rate (ORR)  •Part C: Duration of Response (DoR)  •and 20 more | 220 | 18 Years and older (Adult, Older Adult) | All | •Infinity Pharmaceuticals, Inc. |
| Nivolumab | NCT03667716 | COM701 in Subjects With Advanced Solid Tumors | Recruiting | •Advanced Cancer  •Ovarian Cancer  •Breast Cancer  •Lung Cancer  •Endometrial Cancer  •Ovarian Neoplasm  •Triple Negative Breast Cancer  •Lung Neoplasm  •Neoplasm Malignant | •  •Drug: COM701  •Drug: COM701 with Opdivo (Nivolumab). | Interventional | Phase 1 | •Allocation: NonRandomized  •Intervention Model: Parallel Assignment  •Masking: None (Open Label)  •Primary Purpose: Treatment | •Incidence of subjects with Adverse Events (AEs) as per CTCAE v4.03 and Dose-Limiting Toxicities (DLTs).  •Determine the maximum tolerated dose (MTD) and/ or the recommended dose for expansion (RDFE) (COM701 monotherapy and in combination with a PD-1 inhibitor).  •Incidence of subjects with Anti-COM701 antibody.  •Overall Response Rate as per RECIST v1.1 | 140 | 18 Years and older (Adult, Older Adult) | All | •Compugen Ltd |
| Nivolumab | NCT03432741 | Direct Tumor Microinjection and FDG-PET in Testing Drug Sensitivity in Patients With Relapsed or Refractory NonHodgkin Lymphoma, Hodgkin Lymphoma, or Stage IV Breast Cancer | Recruiting | •Breast Adenocarcinoma  •Recurrent Breast Carcinoma  •Recurrent Hodgkin Lymphoma  •Recurrent Mycosis Fungoides  •Recurrent Non-Hodgkin Lymphoma  •Recurrent Primary Cutaneous TCell Non-Hodgkin Lymphoma  •Refractory Hodgkin Lymphoma  •Refractory Mycosis Fungoides  •Refractory Nodal Marginal Zone Lymphoma  •Refractory Non-Hodgkin Lymphoma  •Refractory Primary Cutaneous TCell Non-Hodgkin Lymphoma  •Stage IV Breast Cancer AJCC v6 and v7 | •Drug: Belinostat  •Drug: Carfilzomib  •Biological: Daratumumab  •Drug: Fludeoxyglucose F-18  •Drug: Gemcitabine Hydrochloride  •Other: Laboratory Biomarker Analysis  •Biological: Nivolumab  •Biological: Obinutuzumab  •Biological: Pembrolizumab  •Procedure: Positron Emission Tomography  •and 4 more | Interventional | Phase 1 | •Intervention Model: Single Group Assignment  •Masking: None (Open Label)  •Primary Purpose: Treatment | •Incidence of drug sensitivity as measured by injection site skin reaction  •Cutaneous response rate based upon the modified Severity Weighted Assessment Tool score  •Feasibility defined as at least 70% of the enrolled patients complete the injection and response evaluation  •Incidence of adverse events  •Nodal disease response rate | 26 | 18 Years and older (Adult, Older Adult) | All | •Mayo Clinic  •National Cancer Institute (NCI) |
| Nivolumab | NCT02834013 | Nivolumab and Ipilimumab in Treating Patients With Rare Tumors | Recruiting | •Acinar Cell Carcinoma  •Adenoid Cystic Carcinoma  •Adrenal Cortex Carcinoma  •Adrenal Gland Pheochromocytoma  •Anal Canal Neuroendocrine Carcinoma  •Anal Canal Undifferentiated Carcinoma  •Appendix Mucinous Adenocarcinoma  •Bartholin Gland Transitional Cell Carcinoma  •Bladder Adenocarcinoma  •Cervical Adenocarcinoma  •and 75 more | •Procedure: Biospecimen Collection  •Biological: Ipilimumab  •Biological: Nivolumab | Interventional | Phase 2 | •Intervention Model: Single Group Assignment  •Masking: None (Open Label)  •Primary Purpose: Treatment | •Overall response rate (ORR) defined as confirmed and unconfirmed complete and partial response  •Incidence of adverse events graded by National Cancer Institute (NCI) Common Terminology Criteria for Adverse Events (CTCAE) version 4.0  •Best response calculated from the sequence of RECIST 1.1 and immunerelated response criteria (irRC) objectives  •Clinical benefit rate defined as complete response, partial response, or stable disease, estimated using both RECIST and irRC  •Overall survival (OS), estimated using both RECIST and irRC  •Progression free survival (PFS), estimated using both RECIST and irRC | 707 | 18 Years and older (Adult, Older Adult) | All | •National Cancer Institute (NCI) |
| Nivolumab | NCT01149356 | RO4929097 And Exemestane in Treating Pre- and Postmenopausal Patients With Advanced or Metastatic Breast Cancer | Terminated | •Estrogen Receptor Positive  •HER2/Neu Negative  •Male Breast Carcinoma  •Recurrent Breast Carcinoma  •Stage IIIB Breast Cancer  •Stage IIIC Breast Cancer  •Stage IV Breast Cancer | •Drug: Exemestane  •Drug: GammaSecretase Inhibitor RO4929097  •Drug: Goserelin Acetate | Interventional | Phase 1 | •Allocation: Randomized  •Intervention Model: Parallel Assignment  •Masking: None (Open Label)  •Primary Purpose: Treatment | •Incidence of treatment emergent adverse events (TEAEs) based on CTCAE version 3 grade  •Time to relapse  •Overall survival | 15 | 18 Years and older (Adult, Older Adult) | All | •National Cancer Institute (NCI) |
| Nivolumab | NCT02922764 | A Study of RGX-104 With or Without Nivolumab in Patients With Advanced Solid Malignancies and Lymphoma | Recruiting | •Malignant Neoplasms | •Drug: RGX-104  •Drug: Nivolumab | Interventional | Phase 1 | •Allocation: NonRandomized  •Intervention Model: Parallel Assignment  •Masking: None (Open Label)  •Primary Purpose: Treatment | •Maximum tolerated dose (MTD), or the maximum tested dose at which multiple dose-limiting toxicities (DLTs) are not observed, of RGX-104 as a single agent, and separately, in combination with nivolumab.  •Overall response rate associated with RGX-104 treatment as a single agent, and separately, in combination with nivolumab.  •Progression-free survival associated with RGX-104 treatment as a single agent, and separately, in combination with nivolumab.  •Number of participants with treatment-emergent adverse events with severity as determined by CTCAE v4.03 associated with RGX-104 treatment as a single agent, and separately, in combination with nivolumab.  •Pharmacokinetics: Maximum Plasma Concentration (Cmax) of RGX-104.  •Pharmacokinetics: Area Under the Curve (AUC) of RGX-104. | 150 | 18 Years and older (Adult, Older Adult) | All | •Rgenix, Inc. |
| Nivolumab | NCT02465060 | Targeted Therapy Directed by Genetic Testing in Treating Patients With Advanced Refractory Solid Tumors, Lymphomas, or Multiple Myeloma (The MATCH Screening Trial) | Recruiting | •Advanced Malignant Solid Neoplasm  •Bladder Carcinoma  •Breast Carcinoma  •Cervical Carcinoma  •Colon Carcinoma  •Colorectal Carcinoma  •Endometrial Carcinoma  •Esophageal Carcinoma  •Gastric Carcinoma  •Glioma  •and 40 more | •Drug: Adavosertib  •Drug: Afatinib  •Drug: Binimetinib  •Drug: Capivasertib  •Drug: Crizotinib  •Other: Cytology Specimen Collection Procedure  •Drug: Dabrafenib  •Drug: Dasatinib  •Drug: Defactinib  •Drug: FGFR Inhibitor AZD4547  •and 14 more | Interventional | Phase 2 | •Allocation: NonRandomized  •Intervention Model: Parallel Assignment  •Masking: None (Open Label)  •Primary Purpose: Treatment | •Objective response rate, defined as the percentage of patients whose tumors have a complete or partial response to treatment  •Overall survival, evaluated specifically for each drug (or step)  •Progression free survival  •Time to progression | 6452 | 18 Years and older (Adult, Older Adult) | All | •National Cancer Institute (NCI) |
| Nivolumab | NCT03126110 | Phase 1/2 Study Exploring the Safety, Tolerability, and Efficacy of INCAGN01876 Combined With Immune Therapies in Advanced or Metastatic Malignancies | Recruiting | •Advanced Malignancies  •Metastatic Cancer | •Drug: INCAGN01876  •Drug: Nivolumab  •Drug: Ipilimumab | Interventional | •Phase 1  •Phase 2 | •Allocation: NonRandomized  •Intervention Model: Parallel Assignment  •Masking: None (Open Label)  •Primary Purpose: Treatment | •1. Phase 1: Safety and tolerability assessed by monitoring frequency, duration, and severity of adverse events (AEs)  •Phase 2: Objective response rate (ORR) based on Response Evaluation Criteria in Solid Tumors (RECIST) v1.1.  •Phase 1: ORR based on RECIST v1.1 and modified RECIST v1.1 (mRECIST v1.1)  •Phase 1 & Phase 2: Duration of response based on RECIST v1.1 and mRECIST v1.1  •Phase 1 & Phase 2: Duration of disease control based on RECIST v1.1 and mRECIST v1.1  •Phase 1 & Phase 2: Progression-free survival based on RECIST v1.1 and mRECIST v1.1  •Phase 1 & Phase 2: Overall survival  •Phase 2: Safety and tolerability assessed by monitoring frequency, duration, and severity of adverse events | 285 | 18 Years and older (Adult, Older Adult) | All | •Incyte Biosciences International Sàrl  •Incyte Corporation |
| Nivolumab | NCT02009449 | A Phase 1 Study of AM0010 in Patients With Advanced Solid Tumors | Active, not recruiting | •Melanoma  •Prostate Cancer  •Ovarian Cancer  •Renal Cell Carcinoma  •Colorectal Carcinoma  •Pancreatic Carcinoma  •Non-small Cell Lung Carcinoma  •Solid Tumors  •Breast Cancer | •Drug: AM0010  •Drug: Paclitaxel or Docetaxel and Carboplatin or Cisplatin  •Drug: FOLFOX (Oxaliplatin/ Leucovorin/5- Fluorouracil)  •Drug: gemcitabine/ nab-paclitaxel  •Drug: Capecitabine  •Drug: Pazopanib  •Drug: Pembrolizumab  •Drug: Paclitaxel  •Drug: nivolumab  •Drug: Gemcitabine/ carboplatin | Interventional | Phase 1 | •Allocation: NonRandomized  •Intervention Model: Single Group Assignment  •Masking: None (Open Label)  •Primary Purpose: Treatment | •Safety and tolerability as measured by incidence of adverse events  •Pharmacokinetic (PK) parameters  •Change in tumor burden measured by volumetric Computer Tomography (CT) or Magnetic Resonance Imaging (MRI) according to immunerelated response criteria (irRC)  •Progression in bone by bone scintigraphy according to Prostate Cancer Working Group 2 (PCWG2) for patients with metastatic castration resistant prostate cancer (CRPC)  •Anti-AM0010 antibody formation | 350 | 18 Years and older (Adult, Older Adult) | All | •ARMO BioSciences |
| Nivolumab | NCT0324117 | A Study Exploring the Safety and Efficacy of INCAGN01949 in Combination With Immune Therapies in Advanced or Metastatic Malignancies | Active, not recruiting | •Advanced Malignancies | •Drug: INCAGN01949  •Drug: Nivolumab  •Drug: Ipilimumab | Interventional | •Phase 1  •Phase 2 | •Allocation: NonRandomized  •Intervention Model: Parallel Assignment  •Masking: None (Open Label)  •Primary Purpose: Treatment | •Phase 1: Participants With Treatment-Emergent Adverse Events (TEAEs) [Safety and Tolerability]  •Phase 2: Objective response rate (ORR) based on Response Evaluation Criteria in Solid Tumors (RECIST) v1.1  •Phase 1 & Phase 2: ORR based on RECIST v1.1 and modified RECIST (mRECIST)  •Phase 1 & Phase 2: Duration of response based on RECIST v1.1 and mRECIST  •Phase 1 & Phase 2: Disease control rate based on RECIST v1.1 and mRECIST  •Phase 1 & Phase 2: Duration of disease control based on RECIST v1.1 and mRECIST  •Phase 1 & Phase 2: Progression-free survival based on RECIST v1.1 and mRECIST  •Phase 1 & Phase 2: Overall survival  •Phase 1 & Phase 2: Participants With Treatment-Emergent Adverse Events (TEAEs) [Safety and Tolerability] | 52 | 18 Years and older (Adult, Older Adult) | All | •Incyte Biosciences International Sàrl  •Incyte Corporation |
| Nivolumab | NCT02890368 | Trial of Intratumoral Injections of TTI-621 in Subjects With Relapsed and Refractory Solid Tumors and Mycosis Fungoides | Recruiting | •Solid Tumors  •Mycosis Fungoides  •Melanoma  •Merkel-cell Carcinoma  •Squamous Cell Carcinoma  •Breast Carcinoma  •Human PapillomavirusRelated Malignant Neoplasm  •Soft Tissue Sarcoma | •Drug: TTI-621 Monotherapy  •Drug: TTI-621 + PD-1/PD-L1 Inhibitor  •Drug: TTI-621 + pegylated interferon-#2a  •Other: TTI-621 + TVec  •Other: TTI-621 + radiation | Interventional | Phase 1 | •Allocation: NonRandomized  •Intervention Model: Parallel Assignment  •Masking: None (Open Label)  •Primary Purpose: Treatment | •Optimal TTI-621 delivery regimen  •Frequency and severity of adverse events  •Preliminary evidence of anti-tumor activity of TTI-621 | 240 | 18 Years and older (Adult, Older Adult) | All | •Trillium Therapeutics Inc. |
