# Supplementary material for: Research Progresses in Immunological Checkpoint Inhibitors for Breast Cancer Immunotherapy
Source: Front Oncol. 2021 Sep 23;11:582664. doi: 10.3389/fonc.2021.582664 (PMC8495193; doi:10.3389/fonc.2021.582664)
Supplement: Supplementary Table 3 — Some ongoing clinical trials of anti-PD-L1 immunotherapeutic interventions of malignancies including breast cancer. [file Table_3.doc]

| **Supplementary table 3,** **Some ongoing clinical trials of anti-PD-L1 immunotherapeutic interventions of** **malignancies including breast cancer** | | | | | | | | | | | | | |
| --- | --- | --- | --- | --- | --- | --- | --- | --- | --- | --- | --- | --- | --- |
| **Drug** | **NCT Number** | **Title** | **Status** | **Conditions** | **Interventions** | **Characteristics** | | | | **Population** | | | **Sponsor/ Collaborators** |
|  |  |  |  |  |  | **Study Type** | **Phase** | **Study Design** | **Outcome Measures** | **Enrollment** | **Age** | **Sex** |  |
| Avelumab | NCT03414658 | The AVIATOR Study: Trastuzumab and Vinorelbine With Avelumab OR Avelumab & Utomilumab in Advanced HER2+ Breast Cancer | Recruiting | •Breast Cancer | •Drug: Vinorelbine  •Drug: Trastuzumab  •Drug: Avelumab  •Drug: Utomilumab | Interventional | Phase 2 | •Allocation: Randomized  •Intervention Model: Crossover Assignment  •Masking: None (Open Label)  •Primary Purpose: Treatment | •Progression Free Survival  •Objective Response Rate  •Duration of Response  •Overall Survival  •Safety and Tolerability | 100 | 18 Years and older (Adult, Older Adult) | All | •Ian E. Krop, MD, PhD  •Pfizer  •Breast Cancer Research Foundation  •Johns Hopkins University  •Dana-Farber Cancer Institute |
| Avelumab | NCT03573648 | Neoadjuvant Tamoxifen, Palbociclib, Avelumab in Estrogen Receptor Positive Breast Cancer | Not yet Recruiting | •Breast Cancer | •Drug: Avelumab  •Drug: Tamoxifen  •Drug: Palbociclib | Interventional | Phase 2 | •Allocation: Randomized  •Intervention Model: Parallel Assignment  •Masking: None (Open Label)  •Primary Purpose: Treatment | •Clinical Complete Response  •Safety and Tolerability as determined by number of patients who experience Adverse Events | 40 | 18 Years and older (Adult, Older Adult) | All | •Sidney Kimmel Comprehensive Cancer Center at Johns Hopkins  •Pfize |
| Avelumab | NCT02926196 | Adjuvant Treatment for High-risk Triple Negative Breast Cancer Patients with the Anti-PD-l1 Antibody Avelumab | Recruiting | •Triple Negative Breast Neoplasms | •Drug: MSB0010718C | Interventional | Phase 3 | •Allocation: Randomized  •Intervention Model: Parallel Assignment  •Masking: None (Open Label)  •Primary Purpose: Treatment | •Disease free survival  •Disease free survival in PD-L1-positive patients  •Overall survival  •Safety profile | 335 | 18 Years and older (Adult, Older Adult) | All | •Istituto Oncologico Veneto IRCCS  •University of Padova  •Dipartimento di scienze chirurgiche, Oncologiche e Gastroenterologic |
| Avelumab | NCT03147287 | Palbociclib After CDK and Endocrine Therapy (PACE) | Recruiting | •Metastatic Breast Cancer | •Drug: Palbociclib  •Drug: Fulvestrant  •Drug: Avelumab | Interventional | Phase 2 | •Allocation: Randomized  •Intervention Model: Parallel Assignment  •Masking: None (Open Label)  •Primary Purpose: Treatment | •Progression Free Survival  •Overall Response Rate  •Incidence of TreatmentEmergent Adverse Events [Safety and Tolerability) | 220 | 18 Years and older (Adult, Older Adult) | All | •Dana-Farber Cancer Institute  •Pfizer |
| Avelumab | NCT03175666 | NANT Triple Negative Breast Cancer (TNBC) Vaccine: Combination Immunotherapy in Subjects With TNBC Who Have Progressed on or After Anthracycline-based Chemotherapy | Not yet Recruiting | •Triple Negative Breast Cancer | •Biological: avelumab  •Biological: bevacizumab  •Drug: capecitabine  •Drug: cisplatin  •Drug: cyclophosphamide  •Drug: 5-Fluorouracil  •Drug: Leucovorin  •Drug: nabpaclitaxel  •Drug: Lovaza  •Radiation: Stereotactic Body Radiation Therapy  •and 8 more | Interventional | •Phase 1  •Phase 2 | •Intervention Model: Single Group Assignment  •Masking: None (Open Label)  •Primary Purpose: Treatment | •Incidence of treatmentemergent adverse events (AEs) and serious AEs (SAEs), graded using the National Cancer Institute (NCI) Common Terminology Criteria for Adverse Events (CTCAE) Version 4.03.  •Objective response rate (ORR) by Response Evaluation Criteria in Solid Tumors (RECIST) Version 1.1  •ORR by Immune-related response criteria (irRC )  •ORR by RECIST Version 1.1  •ORR by irRC  •Progression-free survival (PFS) by RECIST Version 1.1  •PFS by irRC  •Overall survival (OS): time from the date of first treatment to the date of death (any cause)  •Duration of response (DR): time from the date of first response (partial response (PR) or complete response (CR)) to the date of disease progression or death (any cause) whichever occurs first  •Disease control rate (DCR): confirmed complete response, partial response, or stable disease lasting for at least 2 months  •Patient-reported outcomes (PRO) using the Functional Assessment of Cancer Therapy (FACT) Breast | 79 | 18 Years and older (Adult, Older Adult) | All | •NantKwest, Inc. |
| Avelumab | NCT03387085 | QUILT-3.067: NANT Triple Negative Breast Cancer (TNBC) Vaccine: Molecularly Informed Integrated Immunotherapy in Subjects with TNBC Who Have Progressed on or After Standard-of-care Therapy. | Recruiting | •Triple Negative Breast Cancer | •Drug: Aldoxorubicin HCl  •Biological: ALT-803  •Biological: ETBX-011  •Biological: ETBX-051  •Biological: ETBX-061  •Biological: GI-4000  •Biological: GI-6207  •Biological: GI-6301  •Biological: haNK for Infusion  •Biological: avelumab  •and 8 more | Interventional | •Phase 1  •Phase 2 | •Intervention Model: Single Group Assignment  •Masking: None (Open Label)  •Primary Purpose: Treatment | •Incidence of treatmentemergent Adverse Events (AEs) and Serious Adverse Events (SAEs), graded using the NCI CTCAE Version 4.03.  •Objective response rate by RECIST  •Objective response rate by irRC  •Progression-free survival by RECIST during Phase 1b  •PFSl by irRC during Phase 1b  •Overall survival  •Duration of response by RECIST and irRC  •Disease control rate (confirmed complete response, partial response, or stable disease lasting zfor at least 2 months) by RECIST and irRC.  •Patient-reported outcomes of pancreatic cancer symptoms  •PFS by RECIST during Phase 2  •Progression-free survival by irRC during Phase 2 | 79 | 18 Years and older (Adult, Older Adult) | All | •NantKwest, Inc. |
| Avelumab | NCT03554109 | QUILT-3.057: NANT Neoadjuvant Triple- Negative Breast Cancer (TNBC) Vaccine | Not yet Recruiting | •Triple Negative Breast Cancer (TNBC) | •Drug: Leucovorin  •Drug: 5-Fluorouracil  •Drug: Aldoxorubicin HCl  •Drug: nabPaclitaxel  •Biological: ETBX-011  •Biological: ETBX-051  •Biological: ETBX-061  •Biological: GI-4000  •Biological: GI-6207  •Biological: GI-6301  •and 6 more | Interventional | Phase 2 | •Allocation: Randomized  •Intervention Model: Parallel Assignment  •Masking: None (Open Label)  •Primary Purpose: Treatment | •Pathological Complete Response Rate  •Evaluation of safety as determined by incidence or treatment-emergent adverse events  •Evaluate additional measures of efficacy by event-free survival  •Overall survival  •Locoregional relapse  •Distant metastatic rates at 1 year | 376 | 18 Years and older (Adult, Older Adult) | All | •NantKwest, Inc. |
| Avelumab | NCT03409458 | A Dose Escalation and Confirmation Study of PT-112 in Advanced Solid Tumors in Combination With Avelumab | Recruiting | •Non Small Cell Lung Cancer (NSCLC)  •Urothelial Carcinoma (UC)  •Squamous Cell Carcinoma of the Head and Neck (SCCHN)  •Metastatic Breast Cancer (mBC)  •Castration-resistant Prostate Cancer (CRPC) | •Drug: PT-112  •Biological: avelumab | Interventional | •Phase 1  •Phase 2 | •Intervention Model: Single Group Assignment  •Masking: None (Open Label)  •Primary Purpose: Treatment | •Recommended dose (RD) of PT-112 to be used in combination with avelumab for further studies in patients with advanced solid tumors.  •Peak Plasma concentration (Cmax) for PT-112 Injection  •Area under the plasma concentration versus time curve (AUC) for PT-112 Injection  •Dose-limiting toxicities (DLTs)  •Number of patients with Adverse Events (AEs)  •Tumor burden Assessment  •Overall Response Rate (ORR)  •Disease Control Rate (DCR)  •Median Progression-free Survival (mPFS) | 52 | 18 Years and older (Adult, Older Adult) | All | •Phosplatin Therapeutics  •Pfizer  •EMD Serono |
| Avelumab | NCT03330405 | Javelin Parp Medley: Avelumab Plus Talazoparib In Locally Advanced Or Metastatic Solid Tumors | Recruiting | •Avelumab in Combination With Talazoparib Will be Investigated in Patients With Locally Advanced (Primary or Recurrent) or Metastatic Solid Tumors | •Drug: Avelumab Phase 1b  •Drug: Talazoparib Phase 1b  •Drug: Avelumab Phase 2  •Drug: Talazoparib Phase 2 | Interventional | Phase 2 | •Allocation: NonRandomized  •Intervention Model: Sequential Assignment  •Masking: None (Open Label)  •Primary Purpose: Treatment | •Dose Limiting Toxicity (DLT)  •Overall Response (OR)  •Serum concentrations of avelumab  •Anti drug antibody (ADA) levels of avelumab  •OR  •PSA Tumor Marker  •CA-125 Tumor Marker  •Biomarker PD-L1  •Genomic  •Plasma concentrations of talazoparib  •and 8 more | 316 | 18 Years and older (Adult, Older Adult) | All | •Pfizer |
| Avelumab | NCT03217747 | Study to Evaluate the Safety and Tolerability of Avelumab in Combination With Other AntiCancer Therapies in Patients With Advanced Malignancies | Recruiting | •Malignant Neoplasm of Breast  •Malignant Neoplasms of Bone and Articular Cartilage  •Malignant Neoplasms of Digestive Organs  •Malignant Neoplasms of Eye Brain and Other Parts of Central Nervous System  •Malignant Neoplasms of Female Genital Organs  •Malignant Neoplasms of Illdefined Secondary and Unspecified Sites  •Malignant Neoplasms of Independent (Primary) Multiple Sites  •Malignant Neoplasms of Lip Oral Cavity and Pharynx  •Malignant Neoplasms of Male Genital Organs  •Malignant Neoplasms of Mesothelial and Soft Tissue  •and 4 more | •Drug: Avelumab  •Drug: Utomilumab  •Drug: PF-04518600  •Radiation: Radiation  •Drug: Cisplatin | Interventional | •Phase 1  •Phase 2 | •Allocation: NonRandomized  •Intervention Model: Parallel Assignment  •Masking: None (Open Label)  •Primary Purpose: Treatment | •Adverse Events of Different Treatment Combinations of Avelumab When Administered in Combination with Checkpoint Agonist(s) with or without Radiation, or Radiation and Cisplatin  •Evaluation of CD8 Immune Biomarkers  •Objective Response Rate per RECIST v1.1  •Objective Response Rate per irRECIST  •Progression-Free Survival  •Duration of Response  •Overall Survival | 188 | 18 Years and older (Adult, Older Adult) | All | •M.D. Anderson Cancer Center  •Pfizer |
| Avelumab | NCT01772004 | Avelumab in Metastatic or Locally Advanced Solid Tumors (JAVELIN Solid Tumor) | Active, not recruiting | •Solid Tumors | •Drug: Avelumab | Interventional | Phase 1 | •Intervention Model: Single Group Assignment  •Masking: None (Open Label)  •Primary Purpose: Treatment | •Dose Limiting Toxicity  •Confirmed Best Overall Response (BOR) as per Response Evaluation Criteria in Solid Tumors (RECIST Version 1.1) for Efficacy Expansion Cohorts  •Number of subjects with Treatment-Emergent Adverse Events according to the National Cancer Institute Common Terminology Criteria for Adverse Events (NCICTCAE) Version 4.0  •Pharmacokinetic parameters: AUC (0-t), AUC (0-infinity), #z, Cmax, Tmax, T(1/2) of avelumab  •Immune-related Best Overall Response (irBOR) and Best Overall Response (BOR) according to modified Immune-related response criteria (irRC) and RECIST version 1.1, respectively  •Immune-related Progression-Free Survival (irPFS) time and Progression-Free Survival (PFS) Time according to modified irRC and RECIST version 1.1 , respectively  •Overall Survival Time  •Pharmacodynamic profile of avelumab to include serum levels of cytokines  •Number of subjects with anti-avelumab antibodies  •Level of PD-L1 tumor expression  •and 5 more | 1758 | 18 Years and older (Adult, Older Adult) | All | •EMD Serono  •Merck KGaA |
| Avelumab | NCT02554812 | A Study Of Avelumab In Combination With Other Cancer Immunotherapies In Advanced Malignancies (JAVELIN Medley) | Recruiting | •Advanced Cancer | •Drug: Avelumab  •Drug: Utomilumab  •Drug: PF-04518600  •Drug: PD 0360324 | Interventional | Phase 2 | •Allocation: Randomized  •Intervention Model: Parallel Assignment  •Masking: None (Open Label)  •Primary Purpose: Treatment | •Number of participants with Dose-Limiting Toxicities (DLT)  •Objective Response - Number of Participants With Objective Response  •Cmax of avelumab (MSB0010718C)  •Cmax of PF-05082566  •Ctrough of avelumab (MSB0010718C)  •Ctrough of PF-05082566  •Anti-Drug Antibody (ADA) levels of avelumab (MSB0010718C)  •Anti-Drug Antibody (ADA) levels of PF-05082566  •Time to Tumor Response (TTR)  •Duration of Response (DR)  •and 6 more | 560 | 18 Years and older (Adult, Older Adult) | All | •Pfizer |
| Avelumab | NCT02222922 | A Study Of PF-06647020 For Adult Patients With Advanced Solid Tumors | Recruiting | • Neoplasms | •Drug: PF-06647020 Q3W  •Drug: fluconazole  •Drug: PF-06647020 Q2W  •Drug: PF-06647020 combined with Avelumab | Interventional | Phase 1 | •Allocation: NonRandomized  •Masking: None (Open Label)  •Primary Purpose: Treatment | •Number of participants with Dose-limiting toxicities (DLT) {Part1}  •Number of Q2W participants with Doselimiting toxicities (DLT) {Part1}  •Area Under the Curve from Time Zero to end of dosing interval (AUCtau) for PF-06647020  •Maximum Observed Concentration (Cmax) for PF-06647020  •Systemic Clearance (CL) for PF-06647020  •Volume of distribution at steady state (Vss) for PF-06647020  •Terminal half-life (t1/2) for PF-06647020  •Accumulation ratio (Rac) for PF-06647020  •Time to Reach Maximum Observed Concentration (Tmax) for PF-06647020  •Area Under the Curve From Time Zero to Last Quantifiable Concentration [AUC (0-t)] for PF-06647020  •and 34 more | 190 | 18 Years and older (Adult, Older Adult) | All | •Pfizer |
| Avelumab | NCT0289036 | Trial of Intratumoral Injections of TTI-621 in Subjects With Relapsed and Refractory Solid Tumors and Mycosis Fungoides | Recruiting | •Solid Tumors  •Mycosis Fungoides  •Melanoma  •Merkel-cell Carcinoma  •Squamous Cell Carcinoma  •Breast Carcinoma  •Human PapillomavirusRelated Malignant Neoplasm  •Soft Tissue Sarcoma | •Drug: TTI-621 Monotherapy  •Drug: TTI-621 + PD-1/PD-L1 Inhibitor  •Drug: TTI-621 + pegylated interferon-#2a  •Other: TTI-621 + TVec  •Other: TTI-621 + radiation | Interventional | Phase 1 | •Allocation: NonRandomized  •Intervention Model: Parallel Assignment  •Masking: None (Open Label)  •Primary Purpose: Treatment | •Optimal TTI-621 delivery regimen  •Frequency and severity of adverse events  •Preliminary evidence of anti-tumor activity of TTI-621 | 240 | 18 Years and older (Adult, Older Adult) | All | •Trillium Therapeutics Inc. |
| Atezolizumab | NCT03483012 | Atezolizumab + Stereotactic Radiation in Triple-negative Breast Cancer and Brain Metastasis | Recruiting | Breast Cancer | •Drug: Atezolizumab  •Procedure: Stereotactic radiosurgery (SRS) | Interventional | Phase 2 | •Intervention Model: Single Group Assignment  •Masking: None (Open Label)  •Primary Purpose: Treatment | •Progression Free Survival  •Extracranial Objective Response Rate  •Progression free survival  •Clinical Benefit Rate  •Overall Survival  •Patient-Reported Outcome  •Development of radiation necrosis  •Investigator-Assessed Neurological Evaluation  •Dose Limiting Toxicity  •Abscopal response rate | 45 | 18 Years and older (Adult, Older Adult) | Female | •Dana-Farber Cancer Institut  •Genentech, I |
| Atezolizumab | NCT02530489 | Triple-Negative First-Line Study: Neoadjuvant Trial of NabPaclitaxel and MPDL3280A, a Pdl-1 Inhibitor in Patients With Triple Negative Breast Cancer | Recruiting | Breast Cancer | •Drug: MPDL3280A  •Drug: Nab-paclitaxel | Interventional | Phase 2 | •Intervention Model: Single Group Assignment  •Masking: None (Open Label)  •Primary Purpose: Treatment | •Pathologic Complete Response (pCR) of MPDL3280A in Combination with Nabpaclitaxel  •Progression Free Survival (PFS) | 37 | 18 Years and older (Adult, Older Adult) | All | •M.D. Anderson Cancer Center  •Genentech, Inc |
| Atezolizumab | NCT03147040 | AssessinG Efficacy of Carboplatin and ATezOlizumab in Metastatic Lobular Breast Cancer | Recruiting | Breast Cancer | •Drug: Carboplatin  •Drug: Atezolizumab | Interventional | Phase 2 | •Intervention Model: Single Group Assignment  •Masking: None (Open Label)  •Primary Purpose: Treatment | •Number of patients free of progression at 6 months  •Number of patients free of progression at 6 months in the IR profile subgroup  •Number of patients free of progression at 6 months in the non- IR profile subgroup  •Number of patients free of progression at 12 months  •Objective Response Rate  •Incidence of TreatmentEmergent Adverse Events (Safety and Tolerability)  •Overall Survival | 40 | 18 Years and older (Adult, Older Adult) | All | •The Netherlands Cancer Institute  •Roche Pharma AG |
| Atezolizumab | NCT02708680 | Randomized Phase 2 Study of Atezolizumab and Entinostat in Patients With aTN Breast Cancer With Phase 1b Lead In | Active, not recruiting | Breast Cancer | •Drug: entinostat  •Drug: atezolizumab  •Drug: Placebo | Interventional | Phase 1 Phase 2 | •Allocation: Randomized  •Intervention Model: Parallel Assignment  •Masking: Quadruple (Participant, Care Provider, Investigator, Outcomes Assessor)  •Primary Purpose: Treatment | •Determination of DLT and the MTD and/or RP2D  •Duration of Progressionfree survival using RECIST 1.1  •Progression-free survival based on immune response RECIST (irRECIST)  •Progression-free survival based on immune response RECIST (irRECIST)  •Overall response rate (ORR) based on RECIST 1.1 and irRECIST  •Clinical benefit rate (CBR) based on RECIST 1.1 and irRECIST  •Overall survival (OS)  •Duration of response (DOR)  •Time to response (TTR) | 88 | 18 Years and older (Adult, Older Adult) | Female | •Syndax Pharmaceuticals  •Roche Pharma AG |
| Atezolizumab | NCT03125902 | A Study of Atezolizumab and Paclitaxel Versus Placebo and Paclitaxel in Participants With Previously Untreated Locally Advanced or Metastatic Triple Negative Breast Cancer (TNBC) | Recruiting | •Triple-Negative Breast Cancer | •Drug: Atezolizumab (MPDL3280A), an engineered anti-PDL1 antibody  •Drug: Atezolizumab Placebo  •Drug: Paclitaxe | Interventional | Phase 3 | •Allocation: Randomized  •Intervention Model: Parallel Assignment  •Masking: Double (Participant, Investigator)  •Primary Purpose: Treatment | •Progression-Free Survival (PFS) Assessed Using Response Evaluation Criteria in Solid Tumors Version 1.1 (RECIST v1.1)  •Percentage of Participants Who are Alive Without Progression Event at Month 12 Assessed Using RECIST v1.1  •Overall Survival (OS)  •Percentage of Participants Who are Alive at 12 and 18 Months  •Time to deterioration (TTD) in Global Health Status/ Health Related Quality of Life (HRQoL)  •Percentage of Participants With Objective Response Assessed Using RECIST v1.1  •Duration of Objective Response (DOR) Assessed Using RECIST v1.1  •Percentage of Participants With Clinical Benefit Assessed Using RECIST v1.1  •Minimum Observed Serum Concentration (Cmin) of Atezolizumab  •Maximum Observed Serum Concentration (Cmax) of Atezolizumab  •and 5 more | 540 | 18 Years and older (Adult, Older Adult) | All | •Hoffmann-La Roche |
| Atezolizumab | NCT02924883 | A Study to Evaluate the Efficacy and Safety of Trastuzumab Emtansine in Combination With Atezolizumab or AtezolizumabPlacebo in Participants With Human Epidermal Growth Factor-2 (HER2) Positive Locally Advanced or Metastatic Breast Cancer (BC) Who Received Prior Trastuzumab and Taxane Based Therapy | Active, not recruiting | •Metastatic Breast Cancer | •Drug: Atezolizumab  •Drug: Trastuzumab emtansine  •Other: Placebo | Interventional | Phase 2 | •Allocation: Randomized  •Intervention Model: Parallel Assignment  •Masking: Double (Participant, Investigator)  •Primary Purpose: Treatment | •Progression-Free Survival (PFS) as Determined by Investigator's Tumor Assessment Using Response Evaluation Criteria in Solid Tumors (RECIST) Version 1.1 (v1.1)  •Percentage of Participants with Adverse Events  •Overall Survival (OS)  •Percentage of Participants With Objective Response (OR) as Determined by Investigator's Tumor Assessment Using RECIST v1.1  •Duration of OR as Determined by Investigator's Tumor Assessment Using RECIST v1.1  •Maximum Serum Concentration (Cmax) of Trastuzumab Emtansine  •Serum Concentration of Total Trastuzumab  •Maximu  •Cmax of Atezolizumab  •Percentage of Participants With Anti-therapeutic Antibodies (ATAs) to Atezolizumab  •Percentage of Participants With ATAs to Trastuzumab Emtansine | 202 | 18 Years and older (Adult, Older Adult) | All | •Hoffmann-La Roche |
| Atezolizumab | NCT02425891 | A Study of Atezolizumab in Combination With Nab-Paclitaxel Compared With Placebo With Nab-Paclitaxel for Participants With Previously Untreated Metastatic Triple Negative Breast Cancer (IMpassion130) | Active, not recruiting | •Triple Negative Breast Cancer | •Drug: Atezolizumab (MPDL3280A), an engineered anti-PDL1 antibody  •Drug: NabPaclitaxel  •Drug: Placebo | Interventional | Phase 3 | •Allocation: Randomized  •Intervention Model: Parallel Assignment  •Masking: Double (Participant, Investigator)  •Primary Purpose: Treatment | •Progression Free Survival (PFS) According to Response Evaluation Criteria in Solid Tumors (RECIST) Version 1.1 (v1.1) in all Randomized Participants  •PFS According to RECIST v1.1 in Participants with Detectable Programmed Death-Ligand 1 (PD-L1)  •Overall Survival (OS) in all Randomized Participants  •OS in Participants with Detectable PD-L1  •Percentage of Participants With an Objective Response of Complete Response (CR) or Partial Response (PR) According to RECIST v1.1 in all Randomized Participants  •Percentage of Participants With an Objective Response of CR or PR According to RECIST v1.1 in Participants with Detectable PD-L1  •Duration of Response (DOR) According to RECIST v1.1 in all Randomized Participants  •DOR Acccording to RECIST v1.1 in Participants with Detectable PD-L1  •Time to Deterioration (TTD) in Global Health Status/Health Related Quality of Life According to European Organisation for Research and Treatment of Cancer (EORTC) Quality-of-Life Questionnaire Co | 900 | 18 Years and older (Adult, Older Adult) | All | •Hoffmann-La Roche |
| Atezolizumab | NCT03281954 | Clinical Trial of Neoadjuvant Chemotherapy With Atezolizumab or Placebo in Patients With Triple-Negative Breast Cancer Followed After Surgery by Atezolizumab or Placebo | Recruiting | •Triple Negative Breast Cancer | •Drug: Placebo  •Drug: Atezolizumab | Interventional | Phase 3 | •Allocation: Randomized  •Intervention Model: Parallel Assignment  •Masking: Triple (Participant, Care Provider, Investigator)  •Primary Purpose: Treatment | •Pathologic complete response in the breast and lymph nodes (ypT0/Tis ypN0)  •Event-free survival (EFS)  •Pathologic complete response in the breast (ypT0/Tis)  •Pathologic complete response in the breast and lymph nodes (ypT0 ypN0)  •Positive nodal status conversion rate  •Overall survival (OS)  •Recurrence-free interval (RFI)  •Distant disease-free survival (DDFS)  •Brain metastases free survival  •Frequency of Adverse Events  •and 3 more | 1520 | 18 Years and older (Adult, Older Adult) | All | •NSABP Foundation Inc  •Genentech, Inc.  •Hoffmann-La Roche |
| Atezolizumab | NCT03464942 | Stereotactic Radiation and Immunotherapy in Patients With Advanced Triple Negative Breast Cancer | Not yet recruiting | •Breast Cancer | •Radiation: SABR  •Drug: Atezolizumab | Interventional | Phase 2 | •Allocation: Randomized  •Intervention Model: Parallel Assignment  •Masking: None (Open Label)  •Primary Purpose: Treatment | •Progression Free Survival  •Best Objective Response (BOR) between different SABR regimens + atezolizumab  •Incidence of treatment emergent adverse events (safety and tolerability)  •Progression Free Survival Comparison between different SABR regimens + atezolizumab  •Duration of Response (DOR) between different SABR regimens + atezolizumab  •Disease Control Rate (DCR) between different SABR regimens + atezolizumab  •Time to Treatment Failure between different SABR regimens + atezolizumab  •Overall Survival between different SABR regimens + atezolizumab | 52 | 18 Years and older (Adult, Older Adult) | All | •Peter MacCallum Cancer Centre, Australia  •Trans-Tasman Radiation Oncology Group (TROG) |
| Atezolizumab | NCT01898117 | Triple-B Study;Carboplatin-cyclophosphamide Versus Paclitaxel With or Without Atezolizumab as First-line Treatment in Advanced Triple Negative Breast Cancer | Recruiting | •Breast Cancer | •Drug: Carbo/cyclo  •Drug: Carbo/cyclo + atezolizumab  •Drug: Paclitaxel  •Drug: Paclitaxel + Atezolizumab | Interventional | Phase 2 | •Allocation: Randomized  •Intervention Model: Factorial Assignment  •Masking: None (Open Label)  •Primary Purpose: Treatment | •Validate the BRCA-like test  •Improvement of objective response by adding atezolizumab  •Define predictive biomarkers for objective response gain  •Define predictive biomarkers for PFS gain  •Determine PFS in BRCA like TNBC  •Determine PFS in non BRCA like TNBC  •Overall survival (OS)  •Toxicity of all study regimens  •Determine PFS in cross over  •Determine PFS in TNBC molecular subtypes | 304 | Child, Adult, Older Adult | All | •The Netherlands Cancer Institute  •Borstkanker Onderzoek Groep  •Roche Pharma AG |
| Atezolizumab | NCT02322814 | A Study of Cobimetinib Plus Paclitaxel, Cobimetinib Plus Atezolizumab Plus Paclitaxel, or Cobimetinib Plus Atezolizumab Plus Nab-Paclitaxel as Initial Treatment for Participants With Triple-Negative Breast Cancer That Has Spread | Active, not recruiting | •Breast Cancer | •Drug: Cobimetinib  •Drug: Paclitaxel  •Drug: Placebo  •Drug: Atezolizumab  •Drug: Nab-Paclitaxel | Interventional | Phase 2 | •Allocation: Randomized  •Intervention Model: Parallel Assignment  •Masking: None (Open Label)  •Primary Purpose: Treatment | •Cohort I: Progression-Free Survival, as Determined by Investigator Using Response Evaluation Criteria in Solid Tumors Version 1.1 (RECIST v1.1)  •Cohort II, III: Percentage of Participants With Confirmed Overall Response (OR) (Partial Response [PR] or Complete Response [CR]), as Determined by the Investigator Using RECIST v1.1  •Cohort I, II, III: Overall Survival (OS)  •Cohort I: Percentage of Participants With Confirmed OR (PR or CR), as Determined by the Investigator Using RECIST v1.1  •Cohort I, II, III: Duration of Response (DOR), as Determined by the Investigator Using RECIST v1.1  •Cohort I, II, III: Percentage of Participants With Unconfirmed Overall Response (OR_uc) (Unconfirmed PR or CR), as Determined by the Investigator Using RECIST v1.1  •Cohort II, III: ProgressionFree Survival, as Determined by Investigator Using RECIST v1.1  •Cohort I, II, III: Percentage of Participants With Adverse Events (AEs)  •Cohort I, II, III: Maximum Plasma C Determined by Investigator Using RECIST v1.1 | 169 | 18 Years and older (Adult, Older Adult) | All | •Hoffmann-La Roche |
| Atezolizumab | NCT03726879 | A Study To Evaluate the Efficacy and Safety Of Atezolizumab or Placebo in Combination With Neoadjuvant Doxorubicin + Cyclophosphamide Followed By Paclitaxel + Trastuzumab + Pertuzumab In Early Her2- Positive Breast Cancer | Not yet recruiting | •Breast Cancer | •Drug: Atezolizumab  •Drug: Placebo  •Drug: Doxorubicin  •Drug: Cyclophosphamide  •Drug: Paclitaxel  •Drug: Trastuzumab  •Drug: Pertuzumab | Interventional | Phase 3 | •Allocation: Randomized  •Intervention Model: Parallel Assignment  •Masking: Double (Participant, Care Provider)  •Primary Purpose: Treatment | •Percentage of Participants with Pathological Complete Response (pCR)  •Percentage of Participants with pCR Based on Hormone Receptor Status  •Percentage of Participants with pCR Based on PD-L1 Status  •Event-Free Survival (EFS)  •Disease-Free Survival (DFS)  •Overall Survival (OS)  •Mean Changes From Baseline in Function (Role, Physical)  •Mean Changes From Baseline in Global Health Status  •Percentage of Participants With Adverse Events  •Maximum Serum Concentration (Cmax) of Atezolizumab  •and 5 more | 224 | 18 Years and older (Adult, Older Adult) | All | •Hoffmann-La Roche  •Chugai Pharmaceutical |
| Atezolizumab | NCT03125928 | Clinical Trial of Atezolizumab With Paclitaxel, Trastuzumab, and Pertuzumab in Patients With Metastatic HER-2 Positive Breast Cancer | Recruiting | •HER2-positive Breast Cancer | •Drug: Atezolizumab  •Drug: Paclitaxel  •Drug: Trastuzumab  •Drug: Pertuzumab | Interventional | Phase 2 | •Intervention Model: Single Group Assignment  •Masking: None (Open Label)  •Primary Purpose: Treatment | •Number of participants with treatment-related adverse events  •Antitumor activity of atezolizumab plus the standard regimen of paclitaxel, trastuzumab, and pertuzumab  •Overall survival (OS)  •Time to tumor progression (TTP)  •Time to treatment failure (TTF)  •Progression free survival (PFS)  •Clinical benefit rate (CBR)  •Duration of response (DOR)  •Correlation of biomarkers related to PD-L1 blockade with objective response rate (ORR), CBR, PFS, OS, and DOR.  •Efficacy according to hormone receptor status (ER/PR)  •and 3 more | 50 | 18 Years and older (Adult, Older Adult) | Female | •Fox Chase Cancer Center  •Genentech, Inc. |
| Atezolizumab | NCT03498716 | A Study Comparing Atezolizumab (Anti PD-L1 Antibody) In Combination With Adjuvant Anthracycline/Taxane-Based Chemotherapy Versus Chemotherapy Alone In Patients With Operable Triple-Negative Breast Cancer | Recruiting | •Triple Negative Breast Cancer | •Drug: Atezolizumab  •Drug: Paclitaxel  •Drug: Dosedense Doxorubicin or dose-dense Epirubicin  •Drug: Cyclophosphamide | Interventional | Phase 3 | •Allocation: Randomized  •Intervention Model: Parallel Assignment  •Masking: None (Open Label)  •Primary Purpose: Treatment | •Invasive Disease-Free Survival (iDFS)  •Overall Survival (OS)  •Disease-Free Survival (DFS)  •Recurrence-Free Interval (RFI)  •Distant RFI  •Percentage of participants with adverse events  •Serum concentration of Atezolizumab  •Invasive Disease-Free Survival (iDFS) in PDL1- Selected Patients  •Invasive Disease-Free Survival (iDFS) in Node-Positive Disease  •Invasive Disease Free Survival (iDFS) including second primary non-breast invasive cancer  •and 3 more | 2300 | 18 Years and older (Adult, Older Adult) | All | •Hoffmann-La Roche  •Breast International Group  •Alliance Foundation Trials (AFT)  •Institut Jules Bordet/Clinical Trials Support Unit (IJB/CTSU)  •Frontier Science and Technology Research Foundation Inc (FS) |
| Atezolizumab | NCT03498716 | Study of Triple Combination of Atezolizumab + Cobimetinib + Eribulin (ACE) in Patients With Recurrent/Metastatic Inflammatory Breast Cancer | Recruiting | •Malignant Neoplasm of Breast | •Drug: Cobimetinib  •Drug: Atezolizumab  •Drug: Eribulin | Interventional | Phase 2 | •Intervention Model: Single Group Assignment  •Masking: None (Open Label)  •Primary Purpose: Treatment | •Maximum Tolerated Dose (MTD) of Cobimetinib Plus Atezolizumab and Eribulin (ACE) in Patients With Chemotherapy Resistant Metastatic Inflammatory Breast Cancer  •Overall Response Rate (ORR) of Cobimetinib Plus Atezolizumab and Eribulin (ACE) in Patients With Chemotherapy Resistant Metastatic Inflammatory Breast Cancer  •Clinical Benefit Rate (CBR) of Cobimetinib Plus Atezolizumab and Eribulin (ACE) in Patients With Chemotherapy Resistant Metastatic Inflammatory Breast Cancer  •Duration of Response (DOR) of Cobimetinib Plus Atezolizumab and Eribulin (ACE) in Patients With Chemotherapy Resistant Metastatic Inflammatory Breast Cancer  •Progression Free Survival (PFS) of Cobimetinib Plus Atezolizumab and Eribulin (ACE) in Patients With Chemotherapy Resistant Metastatic Inflammatory Breast Cancer  •Overall Survival (OS) of Cobimetinib Plus Atezolizumab and Eribulin (ACE) in Patients With Chemotherapy Resistant Metastatic Inflammatory Breast Cance | 33 | 18 Years and older (Adult, Older Adult) | Female | •M.D. Anderson Cancer Center  •Genentech, Inc |
| Atezolizumab | NCT03164993 | Atezolizumab Combined With Immunogenic Chemotherapy in Patients With Metastatic Triple-negative Breast Cancer | Recruiting | •Cancer, Breast  •Triple Negative Breast Cancer | •Drug: Atezolizumab  •Drug: Pegylated liposomal doxorubicin  •Drug: Cyclophosphamide  •Other: Placebo | Interventional | Phase 2 | •Allocation: Randomized  •Intervention Model: Parallel Assignment  •Masking: Double (Participant, Investigator)  •Primary Purpose: Treatment | •Assessment of toxicity of combined treatment with Atezolizumab, pegylated liposomal doxorubicin and cyclophosphamide  •Progression-free survival (PFS)  •Objective tumor response rate  •Overall survival  •Duration of response  •Durable tumor response rate (DRR; >6 months)  •Patient reported outcome | 75 | 18 Years and older (Adult, Older Adult) | All | •Oslo University Hospital  •Hoffmann-La Roche  •Norwegian Cancer Society  •St. Olavs Hospital  •Helse Stavanger HF  •University Hospital of North Norway |
| Atezolizumab | NCT03197935 | A Study to Investigate Atezolizumab and Chemotherapy Compared With Placebo and Chemotherapy in the Neoadjuvant Setting in Participants With Early Stage Triple Negative Breast Cancer | Recruiting | •Triple-negative Breast Cancer | •Drug: Atezolizumab (MPDL3280A), an engineered antiPDL1 antibody  •Drug: Placebo  •Drug: Nabpaclitaxel  •Drug: Doxorubicin  •Drug: Cyclophosphamide  •Drug: Filgrastim  •Drug: Pegfilgrastim | Interventional | Phase 3 | •Allocation: Randomized  •Intervention Model: Parallel Assignment  •Masking: Double (Participant, Investigator)  •Primary Purpose: Treatment | •Percentage of Participants with Pathologic Complete Response (pCR) Using American Joint Committee on Cancer (AJCC) Staging System  •Percentage of Participants with pCR in Subpopulation with PD-L1-Selected Tumor Status (tumor-infiltrating immune cell [IC]1/2/3) Using AJCC Staging System  •Event-Free Survival (EFS) Using AJCC Staging System  •Overall survival (OS)  •Changes From Baseline in European Organization for Research and Treatment of Cancer (EORTC) Quality of Life Questionnaire Core 30 (QLQ-C30) Score  •Percentage of Participants with Adverse Events (AEs)  •Serum Concentration of Atezolizumab  •Percentage of Participants with Anti-Drug Antibodies (ADAs) to Atezolizumab | 204 | 18 Years and older (Adult, Older Adult) | All | •Hoffmann-La Roche |
| Atezolizumab | NCT03256344 | A Safety Study of Talimogene Laherparepvec Combined With Atezolizumab for Triple Negative Breast Cancer and Colorectal Cancer With Liver Metastases | Recruiting | •Metastatic Triple Negative Breast Cancer  •Metastatic Colorectal Cancer | •Biological: Talimogene Laherparepvec  •Biological: Atezolizumab | Interventional | Phase 1 | •Intervention Model: Single Group Assignment  •Masking: None (Open Label)  •Primary Purpose: Other | •Subject's incidence of dose limiting toxicities (DLTs)  •Subject's incidence of treatment-emergent adverse events  •Number of subject with clinically relevant laboratory abnormalities  •Objective response rate (ORR)  •Best overall response (BOR)  •Duration of response (DOR)  •Disease control rate (DCR)  •Durable response rate (DRR)  •Progression-free survival (PFS)  •Overall survival (OS)  •Lesion level responses in injected and uninjected tumor lesions | 36 | 18 Years to 99 Years (Adult, Older Adult) | All | •Amgen  •RocheGenentech |
| Atezolizumab | NCT03371017 | A Study of the Efficacy and Safety of Atezolizumab Plus Chemotherapy for Patients With Early Relapsing Recurrent Triple-Negative Breast Cancer | Recruiting | •Triple Negative Breast Neoplasms | •Drug: Atezolizumab  •Drug: Placebo  •Drug: Gemcitabine  •Drug: Capecitabine  •Drug: Carboplatin | Interventional | Phase 3 | •Allocation: Randomized  •Intervention Model: Parallel Assignment  •Masking: Quadruple (Participant, Care Provider, Investigator, Outcomes Assessor)  •Primary Purpose: Treatment | •Overall survival (OS)  •Proportion of patients alive 12 months  •Proportion of patients alive 18 months  •Progression-free survival (PFS) as determined by the investigator according to Response Evaluation Criteria in Solid Tumors version 1.1 (RECIST 1.1)  •Objective response rate (ORR), defined as the proportion of patients with an objective response, defined as a complete response (CR) or a partial response (PR), as determined by the investigator accor  •Duration of objective response (DoR) as determined by the investigator according to RECIST 1.1  •Clinical benefit rate (CBR), defined as the proportion of patients with a CR or a PR or stable disease as determined by the investigator according to RECIST 1.1 | 350 | 18 Years and older (Adult, Older Adult) | All | •Hoffmann-La Roche |
| Atezolizumab | NCT03292172 | 20 NCT03292172 A Study to Evaluate the Safety, Pharmacokinetics and Clinical Activity of RO6870810 and Atezolizumab (PD-L1 Antibody) in Participants With Advanced Ovarian Cancer or Triple Negative Breast Cancer | Recruiting | •Advanced Ovarian Cancer  •Triple Negative Breast Cancer | •Drug: Atezolizumab  •Drug: RO6870810 | Interventional | Phase 1 | •Allocation: Non-Randomized  •Intervention Model: Sequential Assignment  •Masking: None (Open Label)  •Primary Purpose: Treatment | •Group 1: Percentage of Participants With Dose Limiting Toxicities (DLT)  •Groups 1 to 4: Percentage of Participants With Adverse Events (AEs)  •Groups 1 to 4: Percentage of Participants With Change in Vital Signs, Physical Findings, Electrocardiogram (ECG) and Laboratory Parameters  •Groups 3 and 4: Objective Response (OR) as per Response Evaluation Criteria in Solid Tumors (RECIST) v1.1.  •Groups 1 to 4: Maximum concentration (Cmax) of RO6870810 (RO) and Atezolizumab (Ate)  •Groups 1 to 4: Time of maximum concentration (tmax) of RO6870810 and Atezolizumab  •Groups 1 to 4: Clearance (CL) or Apparent Clearance (CL/F) of RO6870810 and Atezolizumab  •Groups 1 to 4: Volume of Distribution (Vd) or Apparent Volume of Distribution (Vd/F) of RO6870810 and Atezolizumab  •Groups 1 to 4: Area Under the Plasma ConcentrationTime Curve From Time Zero to End of t | 116 | 18 Years and older (Adult, Older Adult) | All | •Hoffmann-La Roche |
| Atezolizumab | NCT03595592 | Neoadjuvant Treatment of HER2 Positive Early High-risk and Locally Advanced Breast Cancer | Not yet recruiting | •Invasive Breast Cancer | •Drug: Trastuzumab  •Drug: Pertuzumab  •Drug: Carboplatin  •Drug: Paclitaxel  •Drug: Doxorubicin  •Drug: Cyclophosphamide  •Drug: Atezolizumab  •Procedure: Surgery | Interventional | Phase 3 | •Allocation: Randomized  •Intervention Model: Parallel Assignment  •Masking: None (Open Label)  •Primary Purpose: Other | •Event Free Survival (EFS)  •Pathological complete response (pCR)  •Clinical objective response  •Distant Event Free Survival (DEFS)  •Overall Survival (OS)  •Number of participants with adverse events as a measure of safety and tolerability | 650 | 18 Years and older (Adult, Older Adult) | Female | •Fondazione Michelangelo  •Hoffmann-La Roche |
| Atezolizumab | NCT03206203 | Carboplatin With or Without Atezolizumab in Treating Patients With Stage IV Triple Negative Breast Cancer | Recruiting | •Triple Negative Breast Cancer  •Stage IV Breast Cancer  •HER2 Negative  •Invasive Breast Cancer | •Drug: Atezolizumab  •Drug: Carboplatin  •Other: Laboratory Biomarker  •Other: Quality-of-Life Assessment | Interventional | Phase 2 | •Allocation: Randomized  •Intervention Model: Crossover Assignment  •Masking: None (Open Label)  •Primary Purpose: Treatment | •Progression free survival (PFS)  •Overall response rate (ORR)  •Clinical benefit rate (CBR)  •Duration of response (DOR)  •Overall survival (OS) | 185 | 18 Years and older (Adult, Older Adult) | All | •VanderbiltIngram Cancer Center  •National Cancer Institute (NCI)  •Genentech, Inc |
| Atezolizumab | NCT02605915 | Safety and Pharmacokinetics of Atezolizumab Combination Treatments in Participants With HER2-Positive and HER2- Negative Breast Cancer | Active, not recruiting | •HER2-Positive Metastatic Breast Cancer  •HER2-Negative Metastatic Breast Cancer  •Locally Advanced or Early Breast Cancer | •Drug: Atezolizumab  •Drug: Carboplatin  •Drug: Docetaxel  •Drug: Pertuzumab  •Drug: Trastuzumab  •Drug: Trastuzumab emtansine  •Drug: Doxorubicin  •Drug: Cyclophosphamide | Interventional | Phase 1 | •Allocation: Non-Randomized  •Intervention Model: Parallel Assignment  •Masking: None (Open Label)  •Primary Purpose: Treatment | •Percentage of Participants With Dose Limiting Toxicities (DLT) - Cohort 1A, 1B, 1C, 1D, 1F  •Percentage of Participants With DLT - Cohort 1E  •Percentage of Participants With Adverse Events (AEs) According to National Cancer Institute Common Terminology Criteria for AEs, Version 4.0 (NCI CTCAE V4.0)  •Maximum Serum Concentration (Cmax) of Atezolizumab  •Minimum Serum Concentration (Cmin) of Atezolizumab  •Cmin of Trastuzumab  •Cmin of Trastuzumab Emtansine  •Cmin of Pertuzumab  •Cmin of Doxorubicin  •Cmin of Cyclophosphamide  •and 10 more | 98 | 18 Years and older (Adult, Older Adult) | All | •Hoffmann-La Roche |
| Atezolizumab | NCT03417544 | Atezolizumab + Pertuzumab + Trastuzumab In CNS Mets In BC | Recruiting | •HER2-positive Metastatic Breast Cancer  •Central Nervous System Metastases | •Drug: ATEZOLIZUMAB  •Drug: PERTUZUMAB  •Drug: TRASTUZUMAB | Interventional | Phase 2 | •Intervention Model: Single Group Assignment  •Masking: None (Open Label)  •Primary Purpose: Treatment | •Overall Response Rate in CNS  •Progression Free Survival  •Objective non-CNS response rates  •Duration Response Rate  •Clinical Benefit Rate  •Overall Survival  •Dose Limiting Toxicity  •Patient Reported Outcomes by MDASI-BT  •Patient Reported Outcomes by EQ-5D  •Investigator-Assessed Neurological Evaluation | 33 | 18 Years and older (Adult, Older Adult) | All | •Nancy Lin, MD  •Genentech, Inc.  •Dana-Farber Cancer Institute |
| Atezolizumab | NCT03566485 | Atezolizumab and Cobimetinib or Idasanutlin in Participants With Stage IV or Unresectable Recurrent Estrogen Receptor Positive Breast Cancer | Recruiting | •Stage III Breast Cancer  •Stage IIIA Breast Cancer  •Stage IIIB Breast Cancer  •Stage IIIC Breast Cancer  •Stage IV Breast Cancer  •Estrogen Receptorpositive  •HER2/Neu Negative | •Drug: Atezolizumab  •Drug: Cobimetinib  •Drug: Idasanutlin | Interventional | •Phase 1  •Phase 2 | •Allocation: Randomized  •Intervention Model: Single Group Assignment  •Masking: None (Open Label)  •Primary Purpose: Treatment | •Dose limiting toxicity (DLT) (Phase I)  •Maximum tolerated dose (Phase I)  •Recommended phase II dose (phase I)  •Overall response rate (ORR; by Response Evaluation Criteria in Solid Tumors [RECIST] 1.1) (Phase II)  •Clinical benefit rate (CBR) (Phase II)  •Immune related response criteria (irRC) (Phase II)  •Progression-free survival (PFS) (Phase II)  •Overall survival (OS) (Phase II)  •Incidence of adverse events (Phase II) | 92 | 18 Years and older (Adult, Older Adult) | Female | •VanderbiltIngram Cancer Center  •Genentech, Inc.  •Stand Up To Cancer |
| Atezolizumab | NCT02849496 | Olaparib With or Without Atezolizumab in Treating Patients With Locally Advanced or Metastatic Non-HER2- Positive Breast Cancer | Recruiting | •BRCA1 Gene Mutation  •BRCA2 Gene Mutation  •Metastatic Breast Carcinoma  •Stage III Breast Cancer AJCC v7  •Stage IIIA Breast Cancer AJCC v7  •Stage IIIB Breast Cancer AJCC v7  •Stage IIIC Breast Cancer AJCC v7  •Stage IV Breast Cancer AJCC v6 and v7 | •Drug: Atezolizumab  •Other: Laboratory Biomarker Analysis  •Drug: Olaparib  •Other: Questionnaire Administration | Interventional | Phase 2 | •Allocation: Randomized  •Intervention Model: Crossover Assignment  •Masking: None (Open Label)  •Primary Purpose: Treatment | •Progression-free survival (PFS)  •Overall response rate (ORR) evaluated by immune response criteria and normal Response Evaluation Criteria in Solid Tumors criteria  •Duration of response (DoR) | 72 | 18 Years and older (Adult, Older Adult) | All | •National Cancer Institute (NCI) |
| Atezolizumab | NCT02883062 | Carboplatin and Paclitaxel With or Without Atezolizumab Before Surgery in Treating Patients With Newly Diagnosed, Stage II-III Triple-Negative Breast Cancer | Suspended | •Estrogen Receptor Negative  •HER2/Neu Negative  •Invasive Breast Carcinoma  •Progesterone Receptor Negative  •Stage II Breast Cancer AJCC v6 and v7 Stage IIA Breast Cancer AJCC v6 and v7  •Stage IIB Breast Cancer AJCC v6 and v7  •Stage III Breast Cancer AJCC v7  •Stage IIIA Breast Cancer AJCC v7  •Stage IIIB Breast Cancer AJCC v7  •Stage IIIC Breast Cancer AJCC v7  •Triple-Negative Breast Carcinoma | •Drug: Atezolizumab  •Drug: Carboplatin  •Other: Laboratory Biomarker Analysis  •Procedure: Lumpectomy  •Procedure: Mastectomy  •Drug: Paclitaxel | Interventional | Phase 2 | •Allocation: Randomized  •Intervention Model: Parallel Assignment  •Masking: None (Open Label)  •Primary Purpose: Treatment | •Increase in tumor infiltrating lymphocyte (TIL) percentage measured by histopathologic assay  •Increase in pathologic complete response (pCR) rate | 60 | 18 Years and older (Adult, Older Adult) | Female | •National Cancer Institute (NCI) |
| Atezolizumab | NCT02620280 | Neoadjuvant Therapy in TRIPle Negative Breast Cancer With antiPDL1 | Recruiting | •Invasive Ductal Breast Carcinoma | •Drug: Carboplatin  •Drug: Abraxane  •Drug: MPDL3280A  •Procedure: Surgery  •Drug: Anthra | Interventional | Phase 3 | •Allocation: Randomized  •Intervention Model: Parallel Assignment  •Masking: None (Open Label)  •Primary Purpose: Treatment | •Event Free Survival (EFS)  •Pathological complete response (pCR)  •Clinical objective response  •Distant Event Free Survival (DEFS)  •Number of participants with adverse events as a Measure of Safety and Tolerability | 272 | 18 Years and older (Adult, Older Adult) | Female | •Fondazione Michelangelo |
| Atezolizumab | NCT03101280 | A Combination Study of Rucaparib and Atezolizumab in Participants With Advanced Gynecologic Cancers and TripleNegative Breast Cancer | Recruiting | •Gynecologic Neoplasms | •Drug: Atezolizumab  •Drug: Rucaparib | Interventional | Phase 1 | •Allocation: Non-Randomized  •Intervention Model: Parallel Assignment  •Masking: None (Open Label)  •Primary Purpose: Treatment | •Percentage of Participants With Adverse Events  •Percentage of Participants With Dose-Limiting Toxicities (DLTs) [Part 1]  •Recommended Phase II Dose (RP2D) of Rucaparib for the Combination [Part 1]  •Number of Dose Modifications due to Adverse Events [Part 2]  •Percentage of Participants With Objective Response of Complete Response (CR) or Partial Response (PR) as Determined by Investigator Assessment Using Response Evaluation Criteria in Solid Tumors Version 1.1 (RECIST v1.1)  •Percentage of Participants With Objective Response of CR or PR as Determined by Investigator Assessment Using Immune-Modified RECIST Incorporating ImmuneResponse (Cancer Antigen 125 [CA125] Response) Considerations  •Duration of Response (DOR) as Determined by Investigator Assessment Using Immune-Modified RECIST Incorporating Immune-Response (CA125 Response) Considerations  •Progres | 48 | 18 Years and older (Adult, Older Adult) | Female | •Hoffmann-La Roche |
| Atezolizumab | NCT03199885 | Paclitaxel, Trastuzumab, and Pertuzumab With or Without Atezolizumab in Treating Patients With Metastatic Breast Cancer | Not yet recruiting | •Breast Adenocarcinoma  •HER2/Neu Positive  •Recurrent Breast Carcinoma  •Stage III Breast Cancer AJCC v7  •Stage IIIA Breast Cancer AJCC v7  •Stage IIIB Breast Cancer AJCC v7  •Stage IIIC Breast Cancer AJCC v6 and v7 | •Drug: Atezolizumab  •Other: Laboratory Biomarker Analysis  •Drug: Paclitaxel  •Biological: Pertuzumab  •Other: Placebo  •Other: Quality-of-Life Assessment  •Biological: Trastuzumab | Interventional | Phase 3 | •Allocation: Randomized  •Intervention Model: Parallel Assignment  •Masking: Triple (Participant, Care Provider, Investigator)  •Primary Purpose: Treatment | •Progression free survival  •Overall survival  •Overall objective response, defined as complete response or partial response  •Duration of objective Response  •Cumulative incidence of brain metastases  •Frequency of adverse events, including late immune-related toxicities | 600 | 18 Years and older (Adult, Older Adult) | All | •National Cancer Institute (NCI) |
| Atezolizumab | NCT02478099 | MPDL3280A-treatment-ISTUMCG | Recruiting | •Cancer of the Urinary Tract  •Non Small Cell Lung Cancer  •Breast Cancer | •Drug: MPDL3280A | Interventional | Phase 2 | •Intervention Model: Single Group Assignment  •Masking: None (Open Label)  •Primary Purpose: Treatment | •Evaluation of efficacy of MPDL3280A after investigational imaging as measured by objective response rate  •To evaluate progression free survival (PFS) according to standard RECIST v1.1 as assessed by the investigator.  •To evaluate duration of response (DOR) according to standard RECIST v1.1 as assessed by the investigator.  •To evaluate DOR according to modified RECIST as assessed by the investigator.  •To evaluate ORR according to modified RECIST as assessed by the investigator.  •To evaluate PFS according to modified RECIST as assessed by the investigator.  •To evaluate the safety/ tolerability (by analysis of adverse events) of MPDL3280A.  •To determine the number of patients with and patients without tumor tracer uptake with 89ZrMPDL3280A,89Zr-CD8 antibody study and 18FFB-IL2 (as assessed in the accessory imaging trials) who also demonstrate response to MPDL3280A therapy.  •To correlate PD-L1 biopsy results as assessed in the trials MPDL3280Aimg-042015, 89Zr-CD8 antibody | 79 | 18 Years and older (Adult, Older Adult) | All | •University Medical Center Groningen |
| Atezolizumab | NCT02453984 | MPDL3280A-imaging-ISTUMCG | Recruiting | •Breast Cancer  •Cancer of the Urinary Tract  •Non Small Cell Lung Cancer | •Other: 89ZrMPDL-3280A-PET scans | Interventional | Not Applicable | •Intervention Model: Single Group Assignment  •Masking: None (Open Label)  •Primary Purpose: Diagnostic | •Description of 89ZrMPDL3280A PK by measuring standardized uptake value (SUV) on the 89Zr-MPDL3280A-PET scans  •Tumor and immune cell PD-L1 expression analysis in a fresh pre-treatment biopsy, and if available archival tumor biopsy, will be correlated to 89ZrMPDL3280A tumor uptake, evaluated by measuring standardized uptake value (SUV) on the 89ZrMPDL3280A-PET.  •Safety assessment through summaries of adverse events, changes in laboratory test results, changes in vital signs, and exposure to 89ZrMPDL3280A. Adverse event data will be recorded and summarized according to NCI CTCAE v4.0.  •To assess the level of MPDL3280A target saturation during treatment with MPDL3280A | 39 | 18 Years and older (Adult, Older Adult) | All | •University Medical Center Groningen |
| Atezolizumab | NCT03280563 | A Study of Multiple Immunotherapy-Based Treatment Combinations in Hormone Receptor (HR)- Positive Human Epidermal Growth Factor Receptor 2 (HER2)-Negative Breast Cancer | Recruiting | •Breast Neoplasms | •Drug: Atezolizumab (MPDL3280A), an engineered antiprogrammed deathligand 1 (PD-L1) antibody  •Drug: Bevacizumab  •Drug: Enitinostat  •Drug: Exemestane  •Drug: Fulvestrant  •Drug: Ipatasertib  •Drug: Tamoxifen | Interventional | •Phase 1  •Phase 2 | •Allocation: Randomized  •Intervention Model: Sequential Assignment  •Masking: None (Open Label)  •Primary Purpose: Treatmen | •Percentage of Participants with Objective Response during Stage 1 According to Response Evaluation Criteria in Solid Tumors (RECIST) Version (v) 1.1  •Progression-Free Survival during Stage 1 According to RECIST v1.1  •Percentage of Participants with Clinical Benefit during Stage 1 According to RECIST v1.1  •Overall Survival during Stage 1  •Percentage of Participants Alive at 18 Months during Stage 1  •Duration of Response during Stage 1 According to RECIST v1.1  •Percentage of Participants with Adverse Events (AEs) during Stage 1  •Percentage of Participants with AEs during Stage 2  •Atezolizumab Serum Concentration during Stage 1  •Entinostat Serum and Plasma Concentration during Stage 1  •and 10 more | 111 | 18 Years and older (Adult, Older Adult) | Female | •Hoffmann-La Roche |
| Atezolizumab | NCT03395899 | Pre-operative Immunotherapy Combination Strategies in Breast Cancer | Recruiting | •Breast Cancer  •Estrogen Receptorpositive Breast Cance | •Drug: Atezolizumab  •Drug: Cobimetinib  •Drug: Ipatasertib  •Drug: Bevacizumab | Interventional | Phase 2 | •Allocation: Randomized  •Intervention Model: Parallel Assignment  •Masking: None (Open Label)  •Primary Purpose: Other | •2-fold Increase in GzmB+ CD8+ T cell levels  •Status and changes of the biomarkers listed below in pre- and endof treatment tumour and/or blood samples: Immunephenotyping, CD8, PD-L1, MHC-I  •Percentage change in Ki67 expression between preand end of study-treatment tumour biopsies  •Percentage change in caspase3 expression  •Incidence, nature and severity of adverse events with severity determined according to CTCAE v4.03. | 160 | 18 Years and older (Adult, Older Adult) | Female | •Queen Mary University of London  •Kliniken EssenMitte  •MedSIR  •Asan Medical Center  •Hoffmann-La Roche |
| Atezolizumab | NCT02914470 | Carboplatin-cyclophosphamide Combined With Atezolizumab | Active, not recruiting | •Breast Cancer  •Cervix Cancer  •Ovarian Cancer  •Endometrial Cancer | •Drug: carbplatin, cyclophophamide, atezolizumab | Interventional | Phase 1 | •Intervention Model: Single Group Assignment  •Masking: None (Open Label)  •Primary Purpose: Treatment | •Toxicity; Incidence of toxicity, graded according to National Cancer Institute Common Toxicity Criteria (NCI-CTC) version 4.03  •Overall Response Rate | 12 | 18 Years and older (Adult, Older Adult) | All | •The Netherlands Cancer Institute  •Roche Pharma AG |
| Atezolizumab | NCT03424005 | A Study Evaluating the Efficacy and Safety of Multiple Immunotherapy-Based Treatment Combinations in Patients With Metastatic Triple-Negative Breast Cancer (Morpheus-TNBC) | Recruiting | •Triple Negative Breast Cancer | •Drug: Capecitabine  •Drug: Atezolizumab  •Drug: Ipatasertib  •Drug: SGN-LIV1A  •Drug: Bevacizumab  •Drug: Cobimetinib  •Drug: Chemotherapy (Gemcitabine + Carboplatin or Eribulin) | Interventional | •Phase 1  •Phase 2 | •Allocation: Randomized  •Intervention Model: Parallel Assignment  •Masking: None (Open Label)  •Primary Purpose: Treatment | •Objective Response Rate (ORR)  •Progression Free Survival (PFS)  •Disease Control Rate (DCR)  •Overall Survival (OS)  •Overall Survival (at specific time-points)  •Duration of Response (DOR)  •Percentage of Participants with Adverse Events  •Serum Concentration of Atezolizumab  •Plasma Concentration of Ipatasertib  •Plasma or Serum Concentration of SNGLIV1A  •Plasma Concentration of Cobimetinib  •Plasma Concentration of Capecitabine | 260 | 18 Years and older (Adult, Older Adult) | All | •Hoffmann-La Roche  •Seattle Genetics, Inc. |
| Atezolizumab | NCT03430518 | Durvalumab and Eribulin in Her2-negative Metastatic Breast Cancer and Recurrent Ovarian Cancer | Recruiting | •HER2-Negative Metastatic Breast Cancer  •Recurrent Ovarian Cancer | •Drug: Durvalumab  •Drug: Eribulin | Interventional | Phase 1 | •Intervention Model: Single Group Assignment  •Masking: None (Open Label)  •Primary Purpose: Treatment | •The dose-limiting toxicity (DLT) rate  •Number of Adverse events  •Objective response rate (ORR)  •Progression free survival (PFS)  •Overall survival (OS)  •ECOG performance status | 12 | 18 Years and older (Adult, Older Adult) | All | •Amy Tiersten  •AstraZeneca  •Eisai Inc.  •Icahn School of Medicine at Mount Sinai |
| Atezolizumab | NCT03650348 | PRS-343 in Combination With Atezolizumab in HER2-Positive Solid Tumors | Recruiting | •HER2-positive Breast Cancer  •HER2-positive Gastric Cancer  •HER2-positive Bladder Cancer  •HER2-positive Solid Tumor | •Drug: PRS-343 in Combination with Atezolizumab | Interventional | Phase 1 | •Intervention Model: Single Group Assignment  •Masking: None (Open Label)  •Primary Purpose: Treatment | •Incidence of DLTs and recommended Phase 2 dose (RP2D) of PRS-343 administered in combination with atezolizumab  •Overall response rate (ORR) per RECIST v1.1  •Duration of response (DOR) per RECIST v1.1  •Rate of complete response (CR) per RECIST v1.1  •Incidence and severity of AEs graded according to the National Cancer Institute Common Terminology Criteria for Adverse Events (NCI CTCAE) version 4.03, and changes in clinical laboratory parameters  •Peak Plasma Concentration (Cmax)  •Area under the plasma concentration versus time curve (AUC)  •Time to maximum dose concentration (Tmax)  •Terminal half life (t1/2)  •Presence and/or concentration of antiPRS-343 and antiatezolizumab antibodies (anti-drug antibodies [ADAs]) | 70 | 18 Years and older (Adult, Older Adult) | All | Pieris Pharmaceuticals, Inc. |
| Atezolizumab | NCT02655822 | Phase 1/1b Study to Evaluate the Safety and Tolerability of CPI-444 Alone and in Combination With Atezolizumab in Advanced Cancers | Recruiting | •Non-Small Cell Lung Cancer  •Malignant Melanoma  •Renal Cell Cancer  •Triple Negative Breast Cancer  •Colorectal Cancer  •Bladder Cancer  •Metastatic Castration Resistant Prostate Cancer | •Drug: CPI-444  •Drug: CPI-444 + atezolizumab | Interventional | Phase 1 | •Allocation: Randomized  •Intervention Model: Parallel Assignment  •Masking: None (Open Label)  •Primary Purpose: Treatment | •Incidence of dose-limiting toxicities (DLTs) of CPI-444 as a single agent and in combination with atezolizumab  •Objective response rate per RECIST v1.1 criteria of CPI-444 as a single agent and in combination with atezolizumab  •Incidence of treatmentemergent adverse events, as assessed by NCI CTCAE v.4.03, of CPI-444 as a single agent and in combination with atezolizumab  •Mean and median Area under the curve (AUC) of CPI-444  •Mean and median Maximum concentration (Cmax) of CPI-444  •Identify the MDL (maximum dose level) of single agent CPI-444 | 323 | 18 Years and older (Adult, Older Adult) | All | •Corvus Pharmaceuticals, Inc.  •Genentech, Inc. |
| Atezolizumab | NCT02543645 | A Study of Varlilumab and Atezolizumab in Patients With Advanced Cancer | Terminated | •Carcinoma, Renal Cell  •Kidney Diseases  •Kidney Neoplasms  •Urogenital Neoplasms  •Urologic Diseases  •Urologic Neoplasms  •Neoplasms by Histologic Type  •Neoplasms  •Clear-cell Metastatic Renal Cell Carcinoma  •Melanoma  •and 4 more | •Drug: Combination of Varlilumab and Atezolizumab | Interventional | Phase 1 | •Intervention Model: Single Group Assignment  •Masking: None (Open Label)  •Primary Purpose: Treatment | Phase l: Safety and tolerability of varlilumab in combination with atezolizumab as measured by incidence of drug related adverse events (AEs), serious drug related AEs, dose-limiting toxicities and laboratory test abnormalities. | 18 | 18 Years and older (Adult, Older Adult) | All | •Celldex Therapeutics  •Genentech, Inc. |
| Atezolizumab | NCT03289962 | A Study of RO7198457 (Personalized Cancer Vaccine [PCV]) as a Single Agent and in Combination With Atezolizumab in Participants With Locally Advanced or Metastatic Tumors | Recruiting | •Melanoma  •Non-Small Cell Lung Cancer  •Bladder Cancer  •Colorectal Cancer  •Triple Negative Breast Cancer  •Renal Cancer  •Head and Neck Cancer  •Other Solid Cancers | •Drug: RO7198457  •Drug: Atezolizumab | Interventional | Phase 1 | •Allocation: NonRandomized  •Intervention Model: Sequential Assignment  •Masking: None (Open Label)  •Primary Purpose: Treatment | •Percentage of Participants with Dose-Limiting Toxicities (DLTs)  •MTD/Recommended Phase 2 Dose (RP2D) of RO7198457  •Percentage of Participants with Adverse Events (AEs) by Severity According to National Cancer Institute (NCI) Common Terminology Criteria for Adverse Events (CTCAE) Version 5.0  •Percentage of Participants with Immune-Mediated Adverse Events (imAEs) by Severity According to NCI CTCAE Version 5.0  •Percentage of Participants by Number of Treatment Cycles Received  •Dose Intensity of RO7198457  •Change from baseline in targeted vital signs  •Change from baseline in targeted clinical laboratory test results  •Change from baseline in ECGs  •Plasma Concentration of (R)-N,N,NTrimethyl-2,3-Dioleyloxy-1- Propanaminium Chloride (DOTMA)  •and 11 more | 567 | 18 Years and older (Adult, Older Adult) | All | •Genentech, Inc.  •Biontech RNA Pharmaceuticals GmbH |
| Atezolizumab | NCT03170960 | Study of Cabozantinib in Combination With Atezolizumab to Subjects With Locally Advanced or Metastatic Solid Tumors | Recruiting | •Urothelial Carcinoma  •Renal Cell Carcinoma  •Non-Small Cell Lung Cancer  •Castration-resistant Prostate Cancer  •Triple Negative Breast Cancer  •Ovarian Cancer  •Endometrial Cancer  •Hepatocellular Carcinoma  •Gastric Cancer  •Gastroesophageal Junction Adenocarcinoma  •and 3 more | •Drug: cabozantinib  •Drug: atezolizumab | Interventional | •Phase 1  •Phase 2 | •Allocation: NonRandomized  •Intervention Model: Sequential Assignment  •Masking: None (Open Label)  •Primary Purpose: Treatment | •Dose Escalation: MTD/ Recommended Dose  •Dose Expansion: ORR  •Incidence and severity of nonserious AEs and SAEs (Safety) | 1000 | 18 Years and older (Adult, Older Adult) | All | •Exelixis |
| Atezolizumab | NCT03579472 | M7824 and Eribulin Mesylate in Treating Participants With Metastatic Triple Negative Breast Cancer | Recruiting | •Anatomic Stage IV Breast Cancer AJCC v8  •Estrogen Receptor Negative  •HER2/Neu Negative  •Progesterone Receptor Negative  •Prognostic Stage IV Breast Cancer AJCC v8  •Triple-Negative Breast Carcinoma | •Drug: Anti-PD-L1/ TGFbetaRII Fusion Protein M7824  •Drug: Eribulin Mesylate | Interventional | Phase 1 | •Intervention Model: Single Group Assignment  •Masking: None (Open Label)  •Primary Purpose: Treatment | •Recommended phase II dose (RP2D) defined as highest dose with dose limiting toxicity (DLT) rate =< 30%  •Incidence of adverse events (AE) | 20 | 18 Years and older (Adult, Older Adult) | All | •M.D. Anderson Cancer Center  •EMD Serono |
| Atezolizumab | NCT02811497 | Study of Azacitidine and Durvalumab in Advanced Solid Tumors | Recruiting | •Microsatellite Stable Colorectal Carcinoma  •Platinum Resistant Epithelial Ovarian Cancer Type II  •Estrogen Receptor Positive and HER2 Negative Breast Cancer | •Drug: Azacitidine  •Drug: Durvalumab | Interventional | Phase 2 | •Intervention Model: Single Group Assignment  •Masking: None (Open Label)  •Primary Purpose: Treatment | •Overall response rate (ORR)  •Disease Control Rate (DCR)  •Progression-free survival (PFS)  •Overall survival (OS)  •Incidence of treatmentemergent adverse events (AEs) | 60 | 18 Years and older (Adult, Older Adult) | All | •University Health Network, Toronto |
| Atezolizumab | NCT02403271 | A Multi-Center Study of Ibrutinib in Combination With MEDI4736 in Subjects With Relapsed or Refractory Solid Tumors | Recruiting | •Non-Small Cell Lung Cancer  •Breast Cancer  •Pancreatic Cancer | •Drug: Ibrutinib  •Drug: Durvalumab (MEDI4736) | Interventional | •Phase 1  •Phase 2 | •Intervention Model: Single Group Assignment  •Masking: None (Open Label)  •Primary Purpose: Treatment | •Phase 1b: Number of participants with adverse events as a measure of safety and tolerability of ibrutinib and MEDI4736 and to find the recommended Phase II dose.  •Phase 2: Efficacy of ibrutinib in combination with MEDI4736 in subjects with relapsed or refractory solid tumors by assessing the ORR per RECIST 1.1.  •Phase 1b: Pharmacokinetics of ibrutinib.  •Phase 1b: Pharmacokinetics of MEDI4736  •Phase 1b: Pharmacodynamics  •Phase 2: Number of participants with adverse events as a measure of safety and tolerability of ibrutinib and MEDI4736  •Phase 2: Pharmacokinetics of ibrutinib  •Phase 2: Pharmacokinetics of MEDI4736  •Phase 2: Pharmacodynamics | 124 | 18 Years and older (Adult, Older Adult) | All | •Pharmacyclics LLC. |
| Atezolizumab | NCT02299999 | SAFIR02_Breast - Efficacy of Genome Analysis as a Therapeutic Decision Tool for Patients With Metastatic Breast Cancer | Recruiting | •Metastatic Breast Cancer | •Drug: AZD2014  •Drug: AZD4547  •Drug: AZD5363  •Drug: AZD8931  •Drug: Selumetinib  •Drug: Vandetanib  •Drug: Bicalutamide  •Drug: Olaparib  •Drug: Anthracyclines  •Drug: Taxanes  •and 9 more | Interventional | Phase 2 | •Allocation: Randomized  •Intervention Model: Parallel Assignment  •Masking: None (Open Label)  •Primary Purpose: Treatment | •Progression-free survival in the targeted drug arm compared to standard maintenance therapy arm  •progression-free survival in patients treated with anti-PDL1 antibody (MEDI4736) compared to standard maintenance therapy arm  •overall survival in each substudy  •overall response rates and changes in tumor size in each substudy  •evaluate safety, in each substudy  •efficacy (response rate, change in tumor size, progression-free survival, overall survival) and safety of each individual targeted agent (substudy 1)  •correlate molecular characteristics in patients with the efficacy endpoints (response rate, progression-free and overall survival) in each substudy | 1460 | 18 Years and older (Adult, Older Adult) | All | •UNICANCER  •Fondation ARC  •AstraZeneca |
| Atezolizumab | NCT02890368 | Trial of Intratumoral Injections of TTI-621 in Subjects With Relapsed and Refractory Solid Tumors and Mycosis Fungoides | Recruiting | •Solid Tumors  •Mycosis Fungoides  •Melanoma  •Merkel-cell Carcinoma  •Squamous Cell Carcinoma  •Breast Carcinoma  •Human PapillomavirusRelated Malignant Neoplasm  •Soft Tissue Sarcoma | •Drug: TTI-621 Monotherapy  •Drug: TTI-621 + PD-1/PD-L1 Inhibitor  •Drug: TTI-621 + pegylated interferon-#2a  •Other: TTI-621 + TVec  •Other: TTI-621 + radiation | Interventional | Phase 1 | •Allocation: NonRandomized  •Intervention Model: Parallel Assignment  •Masking: None (Open Label)  •Primary Purpose: Treatment | •Optimal TTI-621 delivery regimen  •Frequency and severity of adverse events  •Preliminary evidence of anti-tumor activity of TTI-621 | 240 | 18 Years and older (Adult, Older Adult) | All | •Trillium Therapeutics Inc. |
| Durvalumab | NCT02826434 | Adjuvant PVX-410 Vaccine and Durvalumab in Stage II/III Triple Negative Breast Cancer | Recruiting | •Breast Cancer | •Biological: PVX-410  •Biological: Durvalumab  •Drug: Hiltonol | Interventional | Phase 1 | •Intervention Model: Single Group Assignment  •Masking: None (Open Label)  •Primary Purpose: Treatment | •Dose Limiting Toxicity Rate of PVX-410 in Combination With Durvalumab  •Immune Response Rate of cluster designation 8 (CD8)+ Cytotoxic T Lymphocytes (CTLs) to Vaccine-Specific Peptides | 20 | 18 Years and older (Adult, Older Adult) | Female | •Massachusetts General Hospital  •OncoPep, Inc.  •AstraZeneca |
| Durvalumab | NCT02628132 | Study of Safety and Efficacy of Durvalumab in Combination With Paclitaxel in Metastatic Triple Negative Breast Cancer Patients | Recruiting | •Breast Cancer | •Drug: Paclitaxel  •Drug: Durvalumab | Interventional | •Phase 1  •Phase 2 | •Intervention Model: Single Group Assignment  •Masking: None (Open Label)  •Primary Purpose: Treatment | •Number of patients with adverse events (severe and non-severe)  •Objective response rate (ORR)  •Progression free survival | 34 | 18 Years to 65 Years (Adult, Older Adult) | Female | •King Faisal Specialist Hospital & Research Center  •AstraZeneca |
| Durvalumab | NCT03132467 | Evaluating Anti-PD-L1 Antibody (Durvalumab) Plus Anti-CTLA-4 Antibody (Tremelimumab) in HR +/HER2- Breast Cancer | Recruiting | •Breast Cancer  •Hormone Receptor Positive, HER2 Negative Breast Cancer | •Drug: Tremelimumab  •Drug: Durvalumab  •Procedure: Core Breast Tumor Biopsy | Interventional | Early Phase 1 | •Intervention Model: Single Group Assignment  •Masking: None (Open Label)  •Primary Purpose: Treatment | •Feasibility of Enrolling Participants with Hormone Receptor Positive (HR+)/ Human Epidermal Growth Factor Receptor negative (HER2-) Breast Cancer to Trial with Durvalumab Plus Tremelimumab  •Safety of Tremelimumab plus Durvalumab in Participants with Early Stage HR+/HER2- Breast Cancer: Adverse events recorded according to CTCAE 4.03 | 15 | 18 Years and older (Adult, Older Adult) | Female | •M.D. Anderson Cancer Center  •AstraZeneca |
| Durvalumab | NCT02802098 | Abrogation of Chronic Monoclonal Antibody Treatmentinduced T-cell Exhaustion With DURVALUMAB in Advanced HER-2 Negative Breast Cancer | Recruiting | •Metastatic Breast Cancer  •Bevacizumab-alone Maintenance Treatment Progression | •Drug: Durvalumab  •Drug: Bevacizumab | Interventional | Early Phase 1 | •Intervention Model: Single Group Assignment  •Masking: None (Open Label)  •Primary Purpose: Treatment | •Changes Immunodynamics factors: kynurenine, prostaglandin, tryptophan levels  •T-cell exhaustion parameters  •T-cell exhaustion reversion  •Number of participants with treatment-related adverse events as assessed by CTCAE v4.0 | 25 | 18 Years and older (Adult, Older Adult) | Female | •Centro Nacional de Investigaciones Oncologicas CARLOS III  •Fundación CRIS |
| Durvalumab5 | NCT03167619 | Phase II Multicenter Study of Durvalumab and Olaparib in Platinum tReated Advanced Triple Negative Breast Cancer (DORA) | Recruiting | •Triple Negative Breast Cancer | •Drug: Olaparib Oral Product  •Drug: Olaparib Oral Product in combination with Durvalumab | Interventional | Phase 2 | •Allocation: Randomized  •Intervention Model: Parallel Assignment  •Masking: None (Open Label)  •Primary Purpose: Treatment | •Efficacy as assessed by PFS  •Overall Survival Olaparib alone  •Overall Survival olaparib in combination with Durvalumab  •Determine safety and tolerability as determined by adverse events of maintenance olaparib alone.  •Determine safety and tolerability as determined by adverse events of maintenance olaparib plus durvalumab  •Olaparib alone overall response rate (ORR)  •Olaparib in combination with Durvalumab overall response rate (ORR) | 60 | 21 Years and older (Adult, Older Adult) | Female | •Duke University  •AstraZeneca |
| Durvalumab | NCT03608865 | Durvalumab (MEDI4736) and Tremelimumab in Hormone Receptor-positive, Hypermutated Metastatic Breast Cancer Identified by Whole Exome Sequencing | Recruiting | •Metastatic Breast Cancer | •Drug: Durvalumab with Tremelimumab | Interventional | Phase 2 | •Intervention Model: Single Group Assignment  •Masking: None (Open Label)  •Primary Purpose: Treatment | •Objective response rate (ORR) by RECIST 1.1  •Clinical benefit rate (CBR) by RECIST 1.1 defined by complete or partial response or stable disease for at least 24 weeks  •Duration of response (DoR)  •Disease control rate (DCR) by RECIST 1.1  •Progression-free survival (PFS) by RECIST 1.1 | 30 | 19 Years and older (Adult, Older Adult) | Female | •Yonsei University |
| Durvalumab | NCT03356860 | Safety and Efficacy of Durvalumab Combined to Neoadjuvant Chemotherapy in Localized Luminal B HER2(-) and Triple Negative Breast Cancer. | Recruiting | •Breast Cancer  •Triple Negative Breast Cancer  •Luminal B | •Drug: Paclitaxel  •Drug: Epirubicin  •Drug: Cyclophosphamide  •Drug: Durvalumab | Interventional | •Phase 1  •Phase 2 | •Allocation: Randomized  •Intervention Model: Parallel Assignment  •Masking: None (Open Label)  •Primary Purpose: Treatment | •Adverse events  •Pathological response | 57 | 18 Years and older (Adult, Older Adult) | All | •Grand Hôpital de Charleroi  •Cliniques universitaires Saint-LucUniversité Catholique de Louvain |
| Durvalumab | NCT03594396 | Window of Opportunity Trial of Neoadjuvant Olaparib and Durvalumab for Triple Negative or Low ER+ Breast Cancer | Recruiting | •Breast Neoplasms | •Drug: Olaparib  •Drug: Durvalumab | Interventional | •Phase 1  •Phase 2 | •Intervention Model: Single Group Assignment  •Masking: None (Open Label)  •Primary Purpose: Treatment | •The changes of tumor biology  •pathological complete response  •Response rate  •Treatment-related adverse events as assessed by CTCAE v4.0  •Adverse events of special interest and immunemediated adverse events | 25 | 19 Years and older (Adult, Older Adult) | All | •Seoul National University Hospital  •AstraZeneca |
| Durvalumab | NCT02489448 | Neoadjuvant MEDI4736 Concomitant With Weekly Nabpaclitaxel and Dose-dense AC for Stage I-III Triple Negative Breast Cancer | Recruiting | •Breast Neoplasms | •Drug: MEDI4736 | Interventional | •Phase 1  •Phase 2 | •Intervention Model: Single Group Assignment  •Masking: None (Open Label)  •Primary Purpose: Treatment | Pathologic Complete Response (pCR) | 61 | 18 Years and older (Adult, Older Adult) | All | •Yale University |
| Durvalumab | NCT03199040 | Neoantigen DNA Vaccine Alone vs. Neoantigen DNA Vaccine Plus Durvalumab in Triple Negative Breast Cancer Patients Following Standard of Care Therapy | Recruiting | •Triple Negative Breast Cancer  •Triple Negative Breast Neoplasms  •TNBC - TripleNegative Breast Cancer  •Triple-negative Breast Carcinoma | •Drug: Durvalumab  •Biological: Neoantigen DNA vaccine  •Device: TDS-IM system (Inchor Medical Systems)  •Procedure: Peripheral blood draw | Interventional | Phase 1 | •Allocation: Randomized  •Intervention Model: Parallel Assignment  •Masking: None (Open Label)  •Primary Purpose: Treatment | •Safety of neoantigen DNA vaccines given alone or in combination with durvalumab as measured by number of adverse events experienced by patient  •Immune response to neoantigen DNA vaccines given alone or in combination with durvalumab as measured by luminex assay  •Immune response to neoantigen DNA vaccines given alone or in combination with durvalumab as measured by ELISPOT  •Immune response to neoantigen DNA vaccines given alone or in combination with durvalumab as measured by multiparametric flow cytometry | 24 | Age: 18 Years and older (Adult, Older Adult) | Female | •Washington University School of Medicine  •MedImmune LLC |
| Durvalumab | NCT03616886 | Paclitaxel + Carboplatin + Durvalumab With or Without Oleclumab for Previously Untreated Locally Recurrent Inoperable or Metastatic TNBC | Not yet recruiting | •Triple Negative Breast Cancer | •Drug: Paclitaxel  •Drug: Carboplatin  •Drug: MEDI4736  •Drug: MEDI9447 | Interventional | •Phase 1  •Phase 2 | •Allocation: Randomized  •Intervention Model: Parallel Assignment  •Masking: None (Open Label)  •Primary Purpose: Treatment | •Phase I:The adverse events (AEs)  •Phase II: Clinical Benefit of oleclumab in combination with chemotherapy and durvalumab by the comparing the CB rate at 24 weeks from the 1st dose of study drug administration between patients treated with or without the anti-CD73 antibody oleclumab  •Phase I: Recommended phase II dose (RP2D) of oleclumab in combination with chemotherapy and durvalumab  •Phase II: Efficacy of oleclumab in combination with chemotherapy and durvalumab by comparing the OR rate (ORR; CR + PR) and the duration of response (DOR) between patients treated with or without the anti-CD73 antibody oleclumab.  •Phase II: the progressionfree survival (PFS)  •Phase II: overall survival (OS)  •Phase II: AEs assessment based on CTCAE 5.0.  •Phase II: Efficacy, clinical and survival benefits of oleclumab in combination with chemotherapy and durvalumab according to PD-L1 and CD73 expression. | 171 | 18 Years and older (Adult, Older Adult) | Female | •Jules Bordet Institute  •AstraZeneca |
| Durvalumab | NCT03430518 | Durvalumab and Eribulin in Her2-negative Metastatic Breast Cancer and Recurrent Ovarian Cancer | Recruiting | •HER2-Negative Metastatic Breast Cancer  •Recurrent Ovarian Cancer | •Drug: Durvalumab  •Drug: Eribulin | Interventional | Phase 1 | •Intervention Model: Single Group Assignment  •Masking: None (Open Label)  •Primary Purpose: Treatment | •The dose-limiting toxicity (DLT) rate  •Number of Adverse events  •Objective response rate (ORR)  •Progression free survival (PFS)  •Overall survival (OS)  •ECOG performance status | 12 | 18 Years and older (Adult, Older Adult) | All | •Amy Tiersten  •AstraZeneca  •Eisai Inc.  •Icahn School of Medicine at Mount Sinai |
| Durvalumab | NCT02997995 | Durvalumab and Endocrine Therapy in ER+/Her2- Breast Cancer After CD8+ Infiltration Effective Immune-Attractant Exposure | Recruiting | •Breast Cancer  •Estrogen Receptor Positive Tumor  •Menopause  •Hormone Antagonist | •Drug: Immuneattractant  •Drug: Durvalumab  •Procedure: Biopsy | Interventional | Phase 2 | •Intervention Model: Single Group Assignment  •Masking: None (Open Label)  •Primary Purpose: Treatment | •pathological Complete Response  •Number of CD8+ T cell  •Clinical response  •Assessment of Ki67  •Toxicities  •Predictive value of Mutational load for efficacy of Durvalumab  •Predictive value of PDL1 expression for the efficacy of Durvalumab | 240 | 18 Years and older (Adult, Older Adult) | Female | •UNICANCER  •Breast International Group |
| Durvalumab | NCT03544125 | Olaparib and Durvalumab in Treating Participants with Metastatic Triple Negative Breast Cancer | Recruiting | •Triple-Negative Breast Carcinoma  •Anatomic Stage IV Breast Cancer AJCC v8  •Estrogen Receptor Negative  •HER2/Neu Negative  •Progesterone Receptor Negative  •Prognostic Stage IV Breast Cancer AJCC v8 | •Drug: Olaparib  •Biological: Durvalumab | Interventional | Phase 1 | •Intervention Model: Single Group Assignment  •Masking: None (Open Label)  •Primary Purpose: Treatment | •Proportion of completion of Clinical Laboratory Improvement Amendments (CLIA) analytics on pretreatment biopsy before the planned for 4-week biopsy  •Incidence of >= grade 3 adverse events per Common Terminology Criteria for Adverse Events (CTCAE) version (v) 4.03  •Overall response rate (ORR) for olaparib in combination with durvalumab  •Clinical benefit rate (CBR) for olaparib in combination with durvalumab  •Duration of response (DOR) for olaparib in combination with durvalumab  •Progression-free survival (PFS) for olaparib in combination with durvalumab  •Overall survival (OS) olaparib in combination with durvalumab | 8 | 18 Years and older (Adult, Older Adult) | All | •OHSU Knight Cancer Institute |
| Durvalumab | NCT02536794 | MEDI4736 and Tremelimumab in Treating Patients With Metastatic HER2 Negative Breast Cancer | Recruiting | •Estrogen Receptor Negative  •Estrogen Receptor Positive  •HER2/Neu Negative  •Recurrent Breast Carcinoma  •Stage IV Breast Cancer | •Biological: AntiB7H1 Monoclonal Antibody MEDI4736  •Other: Laboratory Biomarker Analysis  •Other: Pharmacological Study  •Biological: Tremelimumab | Interventional | Phase 2 | •Intervention Model: Single Group Assignment  •Masking: None (Open Label)  •Primary Purpose: Treatment | •Response rate of MEDI4736 in combination with Tremelimumab  •Toxicity of MEDI4736 in combination with Tremelimumab  •Overall Survival (OS)  •Progression Free Survival (PFS) | 30 | 18 Years and older (Adult, Older Adult) | Female | •Northwestern University  •MedImmune LLC  •Avon Breast Cancer Foundation  •National Cancer Institute (NCI) |
| Durvalumab | NCT03606967 | Nab-Paclitaxel and Durvalumab With or Without Neoantigen Vaccine in Treating Patients With Metastatic Triple Negative Breast Cancer | Not yet recruiting | •Anatomic Stage IV Breast Cancer AJCC v8  •Estrogen Receptor Negative  •HER2/Neu Negative  •Metastatic TripleNegative Breast Carcinoma  •Progesterone Receptor Negative  •Prognostic Stage IV Breast Cancer AJCC v8 | •Drug: Carboplatin  •Biological: Durvalumab  •Drug: Gemcitabine Hydrochloride  •Drug: Nabpaclitaxel  •Biological: Personalized Synthetic Long Peptide Vaccine  •Drug: Poly ICLC | Interventional | Phase 2 | •Allocation: Randomized  •Intervention Model: Parallel Assignment  •Masking: None (Open Label)  •Primary Purpose: Treatment | •Progression-free survival (PFS)  •Incidence of adverse events assessed using the National Cancer Institute Common Terminology Criteria for Adverse Events (NCI-CTCAE) version 5  •Clinical response rate assessed by Response Evaluation Criteria in Solid Tumors (RECIST) 1.1  •Clinical benefit rate (complete response, partial response, stable disease) assessed by RECIST 1.1  •Overall survival (OS) | 70 | 18 Years and older (Adult, Older Adult) | All | •National Cancer Institute (NCI) |
| Durvalumab | NCT03742102 | A Study of Novel Anti-cancer Agents in Patients With Metastatic Triple Negative Breast Cancer | Not yet recruiting | •Triple Negative Breast Neoplasms | •Drug: Durvalumab  •Drug: Selumetinib  •Drug: Capivasertib  •Drug: Danvatirsen  •Drug: Oleclumab  •Drug: Paclitaxel | Interventional | •Phase 1  •Phase 2 | •Allocation: Randomized  •Intervention Model: Parallel Assignment  •Masking: None (Open Label)  •Primary Purpose: Treatment | •Incidence of adverse events  •Laboratory findings  •Objective response rate (ORR)  •Progression-free survival (PFS).  •Duration of response (DoR)  •Overall survival (OS)  •Blood concentration of durvalumab and novel oncology therapies  •Presence of anti-drug antibodies (ADAs) for durvalumab and applicable novel oncology therapies | 100 | 18 Years to 130 Years (Adult, Older Adult) | Female | •AstraZeneca |
| Durvalumab | NCT03740893 | PHOENIX DDR/Anti-PD-L1 Trial: A Pre-surgical Window of Opportunity and Post-surgical Adjuvant Biomarker Study of DNA Damage Response Inhibition and/or Anti-PD-L1 Immunotherapy in Patients With Neoadjuvant Chemotherapy Resistant Residual Triple Negative Breast Cancer | Not yet recruiting | •Breast Neoplasm | •Drug: AZD6738  •Drug: Olaparib  •Drug: Durvalumab | Interventional | Phase 2 | •Allocation: Randomized  •Intervention Model: Parallel Assignment  •Masking: None (Open Label)  •Primary Purpose: Other | •Cohorts B and C coprimary endpoint #1: Change in mean proliferation index (as measured by tumour cell Ki67 immunohistochemistry) post WOP intervention within post-treatment biopsy compared to pretreatment baseline biopsy.  •Cohorts B and C coprimary endpoint #2: Changes in the proliferation gene expression signature post WOP intervention within the post-treatment biopsy compared to pre-treatment baseline biopsy.  •Cohort D co-primary endpoint #1: Change in CD8+ TILs post antiPD-L1 immunotherapy within the post-treatment biopsy compared to pre-treatment baseline biopsy, assessed using immunohistochemistry.  •Cohort D co-primary endpoint #2: Changes in the interferon gammapositive (IFN#+) signature post WOP intervention within the post-treatment biopsy compared to pretreatment baseline biopsy.  •Incidence of adverse events during trial treatment  •Changes in phosphorylation of ATR and its downstream effectors  •Changes in biomarkers of DNA Damage Response (DDR) and adaptive and | 81 | 18 Years and older (Adult, Older Adult) | All | •Institute of Cancer Research, United Kingdom  •AstraZeneca |
| Durvalumab | NCT03430466 | Anti PD-L1 Antibody + Anti CTLA-4 Antibody in Combination With Hormone Therapy in Patients With Hormone Receptor Positive HER2- negative Recurrent or Metastatic Breast Cancer | Recruiting | •Breast Cancer | •Drug: Durvalumab,Tremeli | Interventional | Phase 2 | •Allocation: NonRandomized  •Intervention Model: Single Group Assignment  •Masking: Double (Participant, Care Provider)  •Primary Purpose: Treatment | Response rate based on RECIST1.1 | 33 | 20 Years and older (Adult, Older Adult) | Female | •Kyoto Breast Cancer Research Network |
| Durvalumab | NCT02484404 | Phase I/II Study of the AntiProgrammed Death Ligand-1 Antibody MEDI4736 in Combination With Olaparib and/or Cediranib for Advanced Solid Tumors and Advanced or Recurrent Ovarian, Triple Negative Breast, Lung, Prostate and Colorectal Cancers | Recruiting | •Lung Cancer  •Breast Cancer  •Ovarian Cancer  •Colorectal Cancer  •Prostate Cancer  •Triple Negative Breast Cancer | •Drug: Olaparib  •Drug: Cediranib  •Drug: MEDI4736 | Interventional | •Phase 1  •Phase 2 | •Allocation: NonRandomized  •Intervention Model: Parallel Assignment  •Masking: None (Open Label)  •Primary Purpose: Treatment | •Ph I Determine the recommended phase II dose (RP2D) and the safety of doublet therapies of MEDI4736/olaparib (MEDI-O) and MEDI4736/ cediranib (MEDI-C) in patients with advanced solid tumors  •Ph II Determine overall response rate of MED-O and MEDI-C in patients with recurrent ovarian cancer | 384 | 18 Years to 99 Years (Adult, Older Adult) | All | •National Cancer Institute (NCI)  •National Institutes of Health Clinical Center (CC) |
| Durvalumab | NCT02811497 | Study of Azacitidine and Durvalumab in Advanced Solid Tumors | Recruiting | •Microsatellite Stable Colorectal Carcinoma  •Platinum Resistant Epithelial Ovarian Cancer Type II  •Estrogen Receptor Positive and HER2 Negative Breast Cancer | •Drug: Azacitidine  •Drug: Durvalumab | Interventional | Phase 2 | •Intervention Model: Single Group Assignment  •Masking: None (Open Label)  •Primary Purpose: Treatment | •Overall response rate (ORR)  •Disease Control Rate (DCR)  •Progression-free survival (PFS)  •Overall survival (OS)  •Incidence of treatmentemergent adverse events (AEs) | 60 | 18 Years and older (Adult, Older Adult) | All | •University Health Network, Toronto |
| Durvalumab | NCT02264678 | Ascending Doses of AZD6738 in Combination With Chemotherapy and/or Novel Anti Cancer Agents | Recruiting | •Adv Solid Malig - H&N SCC, ATM Pro / Def NSCLC, Gastric & Breast Cancer | •Drug: Administration of AZD6738 in combination with carboplatin  •Drug: Administration of AZD6738  •Drug: Administration of AZD6738 in combination with olaparib  •Drug: Administation of AZD6738 in combination with MEDI4736 | Interventional | •Phase 1  •Phase 2 | •Allocation: NonRandomized  •Intervention Model: Parallel Assignment  •Masking: None (Open Label)  •Primary Purpose: Treatment | •Safety and tolerability in terms of AE and SAE (including death), as recorded in safety measures.  •Maximum Observed Plasma Concentration (Cmax) of AZD6738  •Time to observed Cmax (Tmax) for AZD6738  •Area under the plasma concentration-time curve (AUC) for AZD6738  •Maximum Observed Plasma Concentration (Cmax) of Carboplatin  •Time to observed Cmax (Tmax) for Carboplatin  •Area under the plasma concentration-time curve (AUC) for Carboplatin  •Maximum Observed Plasma Concentration (Cmax) of Olaparib  •Time to observed Cmax (Tmax) for Olaparib  •Area under the plasma concentration-time curve (AUC) for Olaparib  •and 10 more | 250 | 18 Years and older (Adult, Older Adult) | All | •AstraZeneca |
| Durvalumab | NCT02299999 | SAFIR02_Breast - Efficacy of Genome Analysis as a Therapeutic Decision Tool for Patients With Metastatic Breast Cancer | Recruiting | •Metastatic Breast Cancer | •Drug: AZD2014  •Drug: AZD4547  •Drug: AZD5363  •Drug: AZD8931  •Drug: Selumetinib  •Drug: Vandetanib  •Drug: Bicalutamide  •Drug: Olaparib  •Drug: Anthracyclines  •Drug: Taxanes  •and 9 more | Interventional | Phase 2 | •Allocation: Randomized  •Intervention Model: Parallel Assignment  •Masking: None (Open Label)  •Primary Purpose: Treatment | •Progression-free survival in the targeted drug arm compared to standard maintenance therapy arm  •progression-free survival in patients treated with anti-PDL1 antibody (MEDI4736) compared to standard maintenance therapy arm  •overall survival in each substudy  •overall response rates and changes in tumor size in each substudy  •evaluate safety, in each substudy  •efficacy (response rate, change in tumor size, progression-free survival, overall survival) and safety of each individual targeted agent (substudy 1)  •correlate molecular characteristics in patients with the efficacy endpoints (response rate, progression-free and overall survival) in each substudy | 1460 | 18 Years and older (Adult, Older Adult) | All | •UNICANCER  •Fondation ARC  •AstraZeneca |
| Durvalumab | NCT02639026 | Trial Of Hypofractionated Radiotherapy In Combination With MEDI4736 And Tremelimumab For Patients With Metastatic Melanoma And Lung, Breast And Pancreatic Cancers | Recruiting | •Metastatic  •Melanoma  •Non Small Cell Lung Cancer (NSCLC)  •Breast Cancer  •Pancreatic Cancer | •Radiation: Radiotherapy  •Drug: MEDI4736  •Drug: Tremelimumab | Interventional | Phase 1 | •Allocation: NonRandomized  •Intervention Model: Parallel Assignment  •Masking: None (Open Label)  •Primary Purpose: Treatment | Number of Adverse Events | 30 | 18 Years and older (Adult, Older Adult) | All | •Abramson Cancer Center of the University of Pennsylvania |
| Durvalumab | NCT02643303 | A Phase 1/2 Study of In Situ Vaccination With Tremelimumab and IV Durvalumab Plus PolyICLC in Subjects With Advanced, Measurable, Biopsyaccessible Cancers | Recruiting | •Head and Neck Squamous Cell Carcinoma  •Breast Cancer  •Sarcoma  •Merkel Cell Carcinoma  •Cutaneous T-Cell Lymphoma  •Melanoma  •Renal Cancer  •Bladder Cancer  •Prostate Cancer | •Drug: Durvalumab  •Drug: Tremelimumab  •Drug: Poly ICLC | Interventional | •Phase 1  •Phase 2 | •Allocation: NonRandomized  •Intervention Model: Parallel Assignment  •Masking: None (Open Label)  •Primary Purpose: Treatment | •To determine the recommended combination doses of the dosing regimen, based on assessment of toxicity and tolerability.  •Clinical Efficacy will be determined by objective response rate (ORR), progression-free survival (PFS), and overall survival (OS) and will be assessed by irRECIST and RECIST1.1.  •Individual durvalumab concentrations  •Individual tremelimumab concentrations after IV and IT administration  •Number of subjects that develop changes in detectable antidrug antibodies to durvalumab  •Number of subjects that develop changes in detectable antidrug antibodies to tremelimumab  •Durvalumab and Tremelimumab Immunogenicity | 102 | 18 Years and older (Adult, Older Adult) | All | •Ludwig Institute for Cancer Research  •MedImmune LLC  •Cancer Research Institute, New York City |
| Durvalumab | NCT01042379 | I-SPY 2 TRIAL: Neoadjuvant and Personalized Adaptive Novel Agents to Treat Breast Cancer | Recruiting | •Breast Neoplasms  •Breast Cancer  •Breast Tumors | •Drug: Standard Therapy  •Drug: AMG 386 with or without Trastuzumab  •Drug: AMG 479 (Ganitumab) plus Metformin  •Drug: MK-2206 with or without Trastuzumab  •Drug: AMG 386 and Trastuzumab  •Drug: T-DM1 and Pertuzumab  •Drug: Pertuzumab and Trastuzumab  •Drug: Ganetespib  •Drug: ABT-888  •Drug: Neratinib  •and 9 more | Interventional | Phase 2 | •Allocation: Randomized  •Intervention Model: Parallel Assignment  •Masking: None (Open Label)  •Primary Purpose: Treatment | •Determine whether adding experimental agents to standard neoadjuvant medications increases the probability of pathologic complete response (pCR) over standard neoadjuvant chemotherapy for each biomarker signature established at trial entry.  •Establishing predictive and prognostic indices based on qualification and exploratory markers to predict pCR and residual cancer burden (RCB).  •To determine three- and five-year relapse-free survival (RFS) and OS among the treatment arms.  •To determine incidence of adverse events (AEs), serious adverse events (SAEs), and laboratory abnormalities of each investigational agent tested.  •MRI Volume | 1920 | 18 Years and older (Adult, Older Adult) | Female | •QuantumLeap Healthcare Collaborative |
| Durvalumab | NCT03518606 | Metronomic Oral Vinorelbine Plus Anti-PD-L1/Anti-CTLA4 ImmunothErapy in Patients With Advanced Solid Tumours | Recruiting | •Advanced Solid Tumours  •Breast Cancer  •Head and Neck Cancer  •Cervix Cancer  •Prostate Cancer | •Drug: Durvalumab + Tremelimumab + metronomic Vinorelbine | Interventional | •Phase 1  •Phase 2 | •Allocation: NonRandomized  •Intervention Model: Parallel Assignment  •Masking: None (Open Label)  •Primary Purpose: Treatment | •Maximum Tolerated Dose (MTD) and the phase II recommended dose (RP2D)  •CBR-24week | 150 | 18 Years and older (Adult, Older Adult) | All | •UNICANCER  •National Cancer Institute, France  •AstraZeneca  •Pierre Fabre Laboratories |
| Durvalumab | NCT02658214 | Durvalumab and Tremelimumab in Combination With First-Line Chemotherapy in Advanced Solid Tumors | Recruiting | •Small Cell Lung Carcinoma  •Carcinoma, Squamous Cell of Head and Neck  •Stomach Neoplasms  •Triple Negative Breast Neoplasms  •Ovarian Neoplasms  •Fallopian Tube Neoplasms  •Peritoneal Neoplasms  •Esophagogastric Junction Neoplasms  •Carcinoma, Pancreatic Ductal  •Esophageal Squamous Cell Carcinoma | •Drug: paclitaxel + carboplatin  •Drug: carboplatin + etoposide  •Drug: gemcitabine + carboplatin  •Drug: nabpaclitaxel (paclitaxel-albumin) + carboplatin  •Drug: oxaliplatin + 5-fluorouracil (5FU) + leucovorin (calcium folinate/ folinic acid)  •Biological: durvalumab  •Biological: tremelimumab  •Drug: nabpaclitaxel (paclitaxel-albumin) + gemcitabine  •Drug: cisplatin + 5- fluorouracil (5FU) | Interventional | Phase 1 | •Allocation: NonRandomized  •Intervention Model: Parallel Assignment  •Masking: None (Open Label)  •Primary Purpose: Treatment | •Laboratory findings (including: clinical chemistry, hematology, and urinalysis)  •Incidence of Adverse Events  •Tumor assessment based on RECIST 1.1 (for cohort 6 only) | 42 | 18 Years to 99 Years (Adult, Older Adult) | All | •AstraZeneca |
| Durvalumab | NCT02734004 | A Phase I/II Study of MEDI4736 in Combination With Olaparib in Patients With Advanced Solid Tumors. | Recruiting | •Ovarian  •Breast  •SCLC  •Gastric Cancers | •Drug: Olaparib  •Drug: MEDI4736  •Drug: Bevacizumab | Interventional | •Phase 1  •Phase 2 | •Intervention Model: Single Group Assignment  •Masking: None (Open Label)  •Primary Purpose: Treatment | •Disease control rate (DCR) [Complete response + Partial response + Stable disease (CR+PR+SD)] based on modified RECIST 1.1  •The safety and tolerability of MEDI4736 in combination with olaparib (±bevacizumab) by assessment of the number and grade of adverse events  •The safety and tolerability of MEDI4736 in combination with olaparib (±bevacizumab) by assessment of physical examination  •The safety and tolerability of MEDI4736 in combination with olaparib (±bevacizumab) by assessment of vital signs  •The safety and tolerability of MEDI4736 in combination with olaparib (±bevacizumab) by assessment of blood samples  •ORR (CR + PR) based on modified RECIST 1.1, and as determined by investigator  •PD-L1 expression in serum samples  •Time to study treatment discontinuation (TDT)  •Overall Survival (OS)  •Percentage change from baseline in tumor size  •and 8 more | 288 | 18 Years to 130 Years (Adult, Older Adult) | All | •AstraZeneca  •IQVIA (formerly QuintilesIMS) |
| Durvalumab | NCT02890368 | Trial of Intratumoral Injections of TTI-621 in Subjects With Relapsed and Refractory Solid Tumors and Mycosis Fungoides | Recruiting | •Solid Tumors  •Mycosis Fungoides  •Melanoma  •Merkel-cell Carcinoma  •Squamous Cell Carcinoma  •Breast Carcinoma  •Human PapillomavirusRelated Malignant Neoplasm  •Soft Tissue Sarcoma | •Drug: TTI-621 Monotherapy  •Drug: TTI-621 + PD-1/PD-L1 Inhibitor  •Drug: TTI-621 + pegylated interferon-#2a  •Other: TTI-621 + TVec  •Other: TTI-621 + radiation | Interventional | Phase 1 | •Allocation: NonRandomized  •Intervention Model: Parallel Assignment  •Masking: None (Open Label)  •Primary Purpose: Treatment | •Optimal TTI-621 delivery regimen  •Frequency and severity of adverse events  •Preliminary evidence of anti-tumor activity of TTI-621 | 240 | 18 Years and older (Adult, Older Adult) | All | •Trillium Therapeutics Inc. |
